# Applications of Transition Metal-Catalyzed *ortho*-Fluorine-Directed C–H Functionalization of (Poly)fluoroarenes in Organic Synthesis

**DOI:** 10.1021/acs.chemrev.3c00793

**Published:** 2024-04-02

**Authors:** Yudha P. Budiman, Robin N. Perutz, Patrick G. Steel, Udo Radius, Todd B. Marder

**Affiliations:** † Department of Chemistry, Faculty of Mathematics and Natural Sciences, Universitas Padjadjaran, 45363 Sumedang, Indonesia; ‡ Department of Chemistry, University of York, York, YO10 5DD, U.K.; C Department of Chemistry, University of Durham, Science Laboratories, South Road, Durham, DH1 3LE, U.K.; ∥ Institute for Inorganic Chemistry, Julius-Maximilians-Universität Würzburg, Am Hubland, 97074 Würzburg Germany; ⊥ Institute for Sustainable Chemistry & Catalysis with Boron, Julius-Maximilians-Universität Würzburg, Am Hubland, 97074 Würzburg Germany

## Abstract

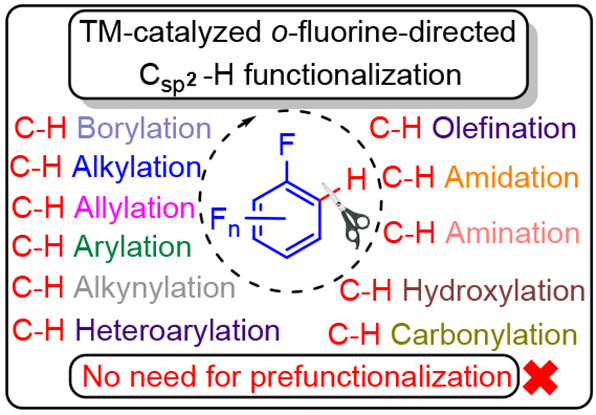

The synthesis of organic compounds efficiently via fewer steps but in higher yields is desirable as this reduces energy and reagent use, waste production, and thus environmental impact as well as cost. The reactivity of C–H bonds *ortho* to fluorine substituents in (poly)fluoroarenes with metal centers is enhanced relative to *meta* and *para* positions. Thus, direct C–H functionalization of (poly)fluoroarenes without prefunctionalization is becoming a significant area of research in organic chemistry. Novel and selective methodologies to functionalize (poly)fluorinated arenes by taking advantage of the reactivity of C–H bonds *ortho* to C–F bonds are continuously being developed. This review summarizes the reasons for the enhanced reactivity and the consequent developments in the synthesis of valuable (poly)fluoroarene-containing organic compounds.

## 1 Introduction

**Figure 1:**
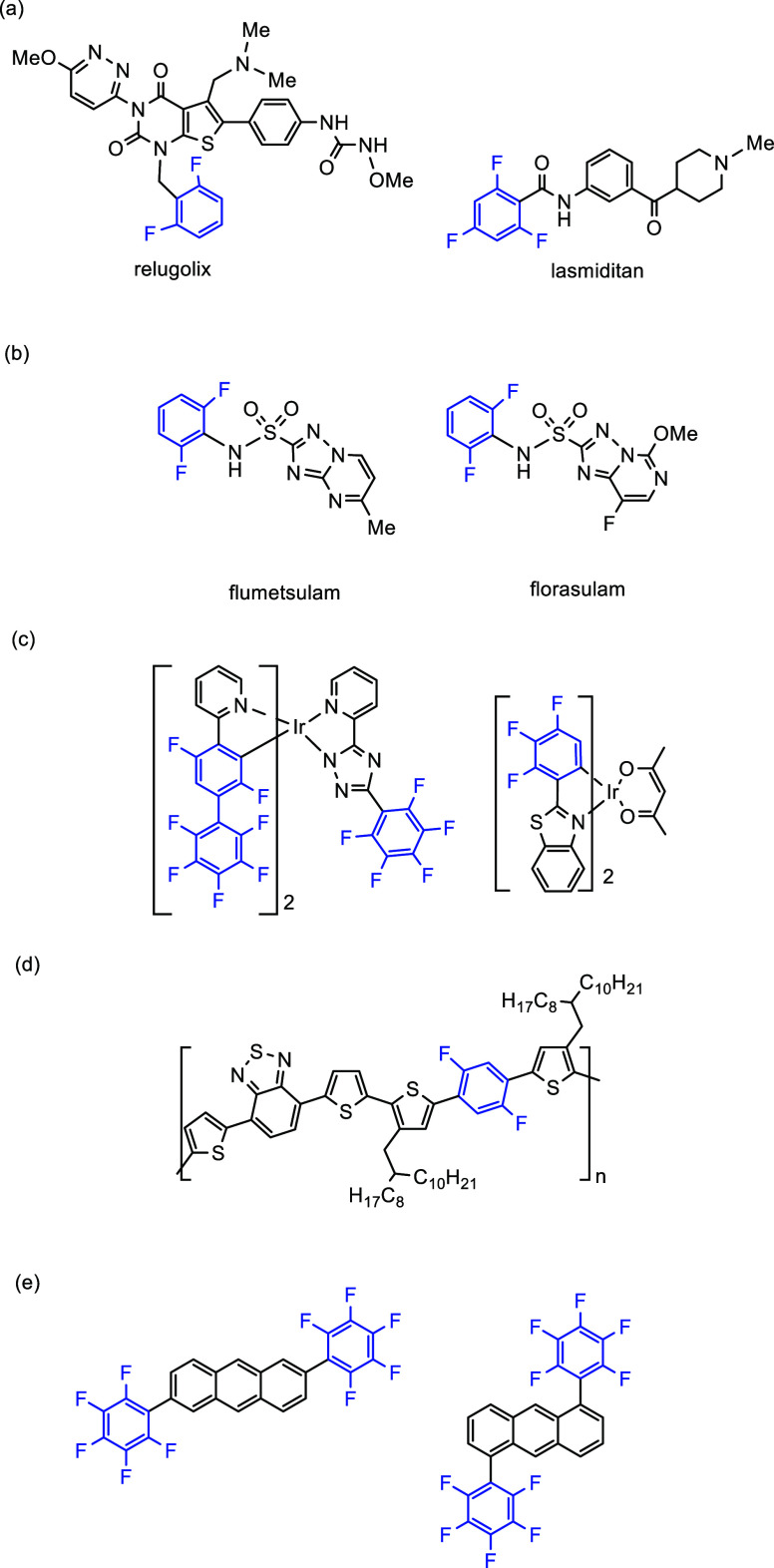
Fluoro-pharmaceuticals. (a) Relugolix (left) is used for advanced prostate cancer. Lasmiditan (right) is used for acute migraine treatment.^9^ (b) Flumetsulam (left) and florasulam (right) are used as herbicides.^13^ (c) Iridium complexes with (poly)fluoroarene ligands for OLEDs.^18^ (d) Fluorinated conjugated polymers for organic semiconductors.^18^ (e) Bis(pentafluorophenyl)anthracene derivatives for OFETs.^41^

Over the last 50 years, the number of fluoro-pharmaceuticals has significantly increased, and an estimated 20% of drugs currently marketed contain fluorine.^1−9^ Considerable research, medicinal, and biological data have been accumulated to make general predictions about the expected effect of fluorination on biological activity, and many reviews have been published on that topic.^1−9^ The incorporation of fluorine substituents provides several physiological advantages such as decreased metabolism, increased solubility and thus deliverability, hydrophobicity, and decreased negative side effects.^4^ For example, relugolix has recently been approved for the treatment of advanced prostate cancer in Japan (Figure 1a, left), and lasmiditan is used for treatment of acute migraine (Figure 1a, right).^9^ In addition, transition-metal ligated fluorinated benzene compounds have also shown promise as antiproliferative agents against HT29 (colon carcinoma) and MCF-7 (breast adenocarcinoma).^10^ Fluorine-containing organic compounds have also shown tremendous potential in other areas of science and industry such as in agrochemicals (Figure 1b),^11−14^ organic light emitting diodes (OLEDs) (Figure 1c),^15−19^ electron-transport materials,^20,21^ crystal engineering,^22−37^ metal–organic frameworks (MOFs),^38^ supramolecular chemistry,^39^ semiconducting materials (Figure 1d),^18,39,40^ and organic-field-effect transistors (OFETs) (Figure 1e).^41^ As such, there is a growing demand for the development of novel synthetic methodologies for the generation of these valuable compounds.^42,43^ In the examples in Figure 1a,b,e, positions 2 and 6 on the aromatic ring (the *ortho* positions) are occupied by fluorine.

As there are no known examples of naturally occurring fluoroarene-containing molecules, these compounds must be accessed via chemical synthesis. In addition, numerous fluoroarenes are commercially available. For an overview of fluoroarene synthesis and applications in pharmaceuticals, agrochemicals, and liquid crystals, see ref (44). Reflecting this, an increasing number of applications of fluoroaryl-containing compounds encourages the development of new strategies to synthesize the required building blocks with maximum efficiency and minimum cost, and, in recent years, significant developments have been made to access these valuable intermediates.

For step- and atom-economical strategies, methods for direct C–H and C–F functionalization of (poly)fluoroarenes have considerable advantages over traditional methods that require more steps to produce preactivated reagents and make this approach “greener”. There are several reviews which concern access to fluorinated organic molecules via transition-metal-catalyzed C–F bond functionalization reactions.^43,45−49^ This review summarizes the applications of transition-metal-catalyzed C–H bond activation, specifically at positions *ortho* to fluorine, for the synthesis of functionalized fluoroarenes. As such, it complements our earlier review,^50^ which focused on the competition between C–F and C–H activation via interaction of fluorinated arenes with transition metal complexes. The *ortho* to fluorine functionalized reagents can be obtained, with selectivity being controlled by either electronic^51−57^ or steric factors^58−61^ that, in turn, reflect both thermodynamics and kinetics. We discuss their selectivity to undergo transition metal-catalyzed direct C–H arylation, heteroarylation, allylation, alkylation, alkynylation, olefination, carbonylation, amination, amidation, and hydroxylation processes, and relevant mechanistic studies.

## 2 Reactivity of the C–H bonds of (Poly)fluoroarenes

Before addressing applications, we start by discussing the properties of C–H bonds that make the reactivities of (poly)fluoroarenes special. Reviews concerning the contribution of the unique properties of their C–H bonds and their interaction with transition metal centers have been published by Eisenstein and Perutz et al.,^50,62^ by Gorelsky et al.,^63^ by Pabst and Chirik,^64^ and by Davies, Macgregor, and McMullin.^65^ In addition, a perspective focusing on the activation and functionalization of C–H and C–F bonds in fluoroarenes by main group elements has recently been published by Hevia et al.^66^ In this section, we address the origins of selectivity between different positions in a particular fluorine-substituted benzene ring for activation by transition metals, for which there is rich experimental evidence. There is little (if any) experimental information about competition between different isomeric fluorobenzenes, e.g., between 1,2-C_6_F_2_H_4_, 1,3-C_6_F_2_H_4_, and 1,4-C_6_F_2_H_4_, although computational studies provide predictions.

### 2.1 C–H Oxidative Addition or Oxidative Cleavage

**Scheme 1:**
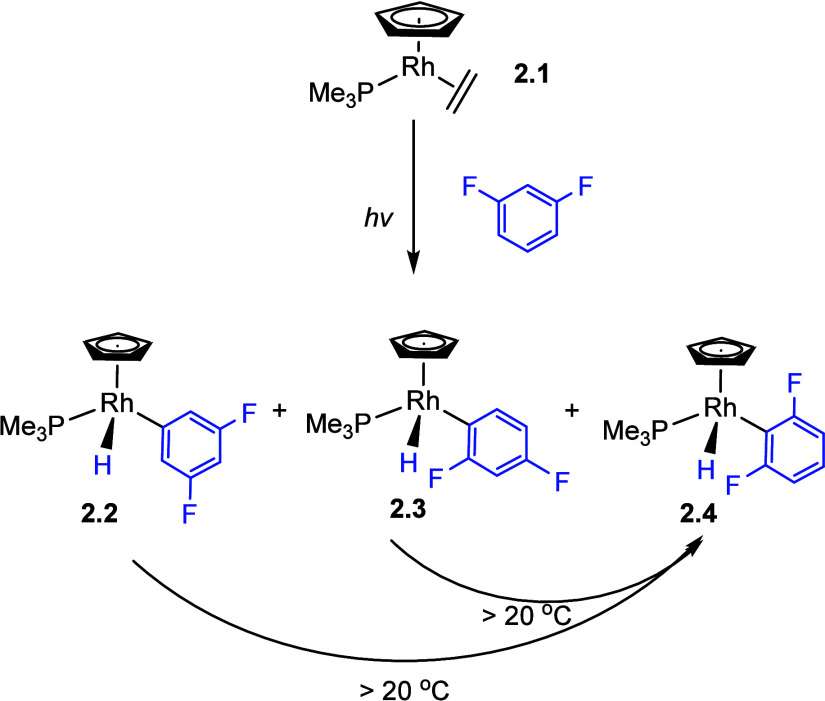
Experimental Preference of C–H Oxidative Product *ortho* to Fluorine at [CpRh(PMe_3_)]^67^

**Scheme 2:**
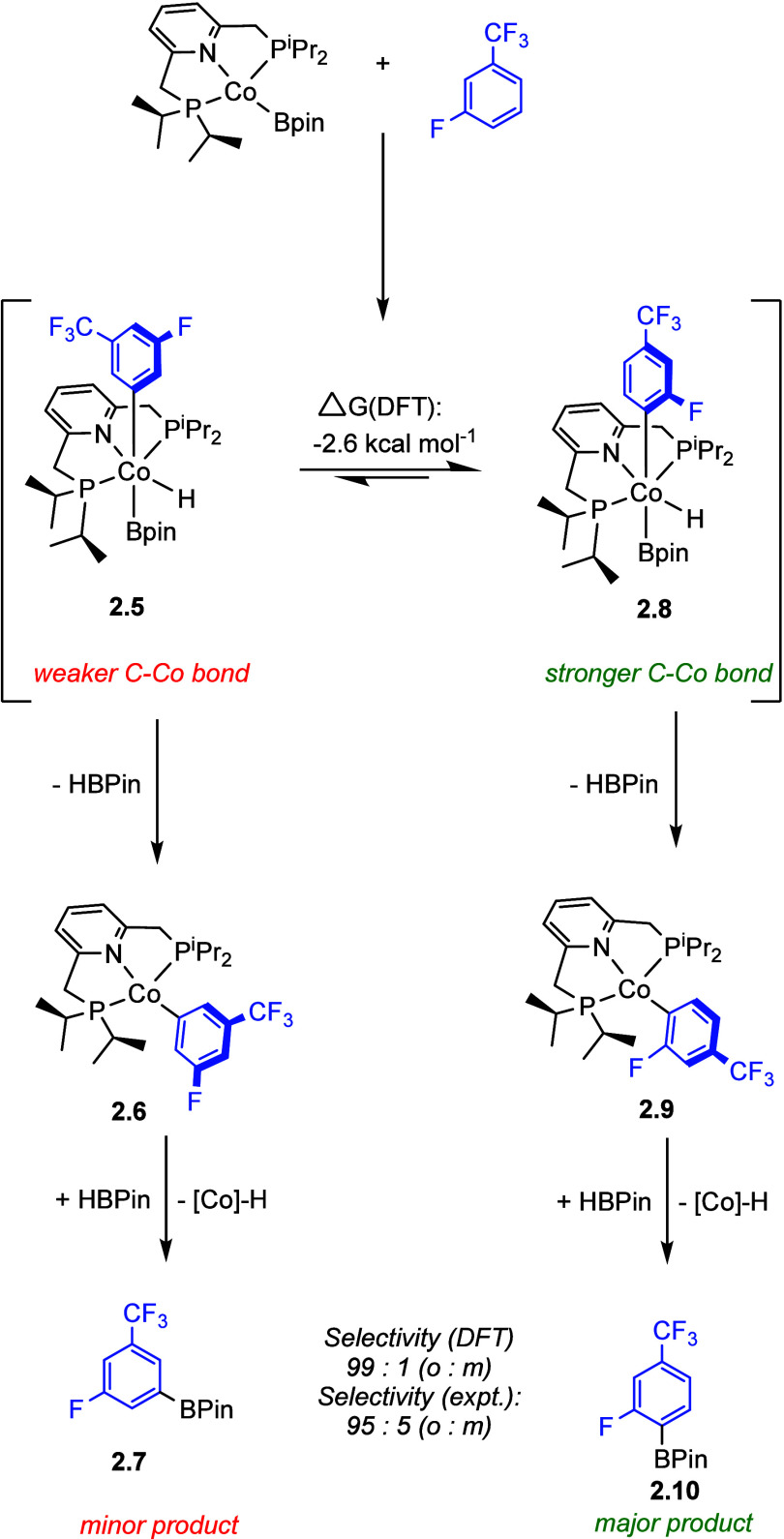
Thermodynamic Control of C–H Oxidative Addition in Cobalt-Catalyzed *ortho* to Fluorine C–H Borylation^72^

Some of the earliest evidence for the regioselectivity of C–H oxidative addition of fluorobenzenes was reported by Perutz and Jones et al.^67^ They investigated the selectivity of the C–H oxidative addition process via photolysis of [CpRh(PMe_3_)(C_2_H_4_)] (Cp = η^5^-C_5_H_5_) (**2.1**) with fluorobenzenes at room temperature or below and the thermal reaction of [Cp*Rh(PMe_3_)(Ph)H] (Cp* = η^5^-C_5_{CH_3_}_5_) with fluorobenzenes C_6_F_n_H_6–n_ (n = 1–5) at 67 °C. Both reactions proceed through the 16-electron fragments [(η^5^-C_5_R_5_)Rh(PMe_3_)] (R = H or Me). These reactions generate the C–H oxidative addition product [(η^5^-C_5_R_5_)Rh(PMe_3_)(C_6_F_n_H_5–n_)H] and show strong evidence for *ortho* selectivity. Of particular interest were the reactions with 1,3-difluorobenzene. A photochemical reaction at very low temperature generated all three possible product isomers (**2.2**–**2.4**), but heating to room temperature caused complete conversion to the isomer with two fluorines *ortho* to Rh (**2.4**). The Cp* complexes behaved similarly, but with a higher barrier to isomerization, proving that this is the thermodynamically most stable isomer (Scheme 1). Considering the established existence of η^2^-arene complexes of CpRh, the isomerization was postulated to proceed via reductive coupling to Rh(η^2^-arene), shifts of Rh around the ring, followed by oxidative cleavage to form the alternative isomer. The origin of this selectivity was later traced to the strengthening of metal-aryl bonds with an increasing number of *ortho* fluorine substituents.^68−72^ The cobalt-catalyzed borylation of 1-fluoro-3-trifluoromethylbenzene provides another example of *ortho* selectivity, which was studied in detail and traced to the same principle of reversible C–H oxidative addition leading to a preference for the strongest Co–C bonds (**2.8**), but this time the oxidative addition is followed by irreversible borylation (**2.10**) (Scheme 2).^72^ Since the early studies, numerous examples of the preferential formation of products with *ortho* fluorine substituents have been reported, demonstrating that this is a general principle, as shown in this review. This may be the result of thermodynamic or kinetic factors, or both.

#### 2.1.1. Thermodynamic Factors

The formation of a metal–carbon bond by reaction of a fluoroarene by C–H oxidative addition (or related reactions) involves cleavage of a fluoroaryl C–H bond and formation of a metal–C(fluoroaryl) bond (eq 1). In simple terms, the electronic energy of the reaction corresponds to the difference in bond dissociation energies (BDEs) between the H–C and H[M]–C bonds, where [M] is the metal–ligand fragment. The key energies then relate to the homolytic cleavage of H–C and H[M]–C bonds (eqs 2 and 3, see also the review by Xue et al.).^73^ (The H[M]–C(fluoroaryl) bonds are abbreviated subsequently as M–C bonds.)
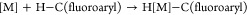
1
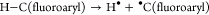
2
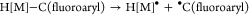
3Both the H–C and M–C BDEs vary very significantly with the number and position of the fluorine substituents. As shown in more detail below, the principal determinant is the number of fluorine substituents *ortho* to the bond that is cleaved or formed in the reaction. The bond strengths increase with the number of *ortho* fluorine substituents, but the M–C bonds increase in strength much more rapidly than the H–C bonds. The effect of *para* and *meta* fluorines is much more minor. The resultant thermodynamic preference for *ortho* fluorine substituents has been observed experimentally through the interconversion of isomers (Scheme 1)^67^ and through the experimental estimation of bond energies.^69^

Systematic testing of the energetics of all possible isomers is rarely, if ever, possible by experiment, but has been achieved by computation. The calculated M–C bond energies (relative to that of M–C_6_H_5_) may be fitted to a simple linear function of the number of *ortho* and *meta*/*para* fluorines (eq 4).
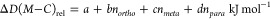
4

**Figure 2:**
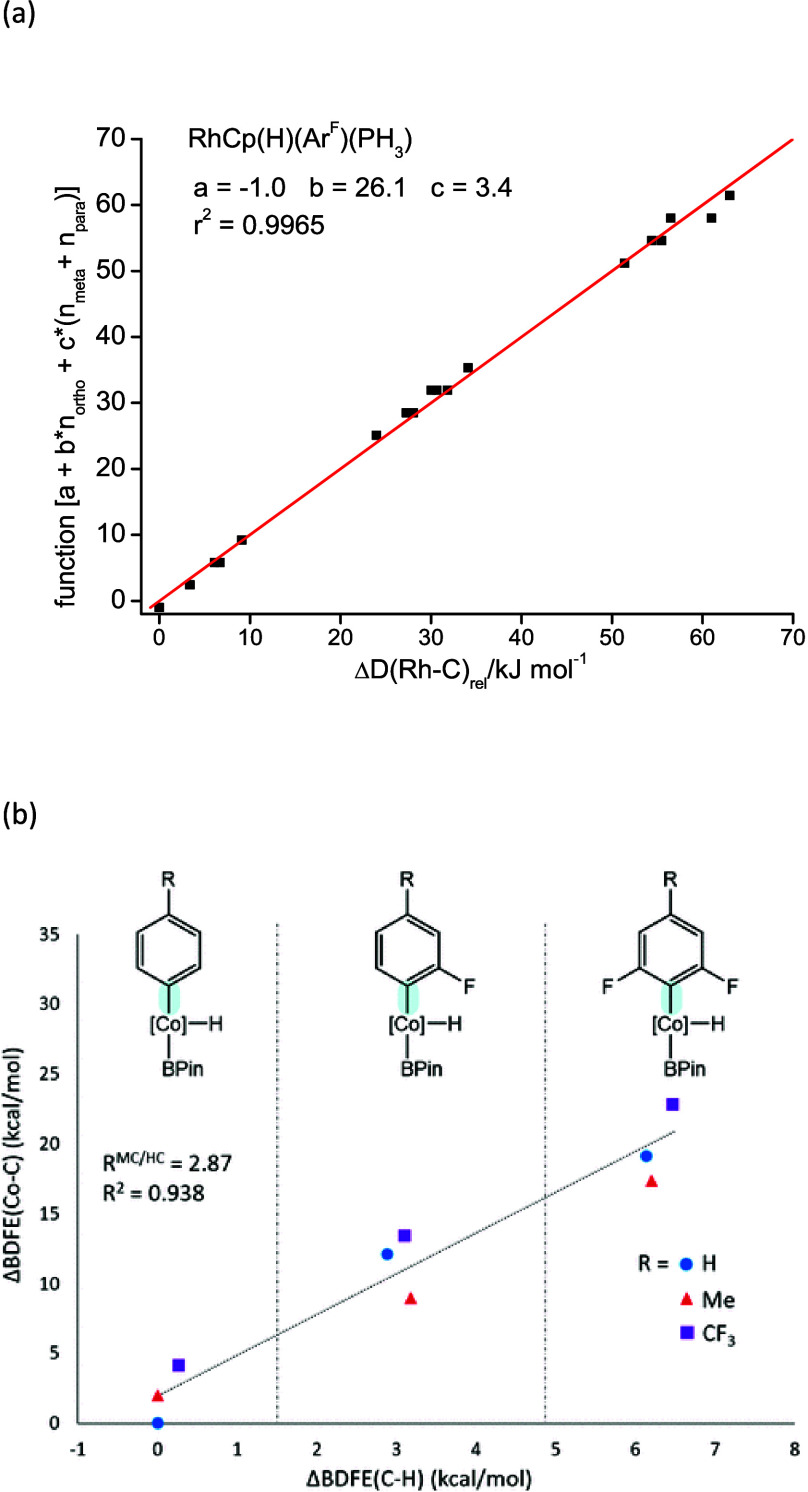
(a) Calculated contributions to Rh–C bond dissociation energy in kJ/mol from *ortho* and *meta*/*para* fluorine substitution for [CpRh(PH_3_)(H)(Ar_F_)]. Each point represents a different fluorinated benzene. The straight line represents the function in eq 1. Reproduced with permission from ref (71). Copyright 2009 American Chemical Society. (b) Correlation between the Co–C bond dissociation free energies (BDFEs) of the type represented by **2.5** and **2.8** and the C–H BDFEs in kcal/mol of the corresponding arenes. The zero points for the *x*- and *y*- axes correspond to the values for the C–H BDFE in benzene and the Co–C BDFE in the phenyl analogue of **2.5**, respectively. Reproduced with permission from ref (72). Copyright 2019 American Chemical Society. Note the way that the points fall into groups for 0, 1, or 2 *ortho* fluorines in both graphs.

The resultant parameters show the dominance of the *ortho* contributions. For example, the parameters for [CpRh(PH_3_)(C_6_H_5–n_F_n_)] (n = 0–5) are *a* = −1.0 ± 0.6, *b* = 26.1 ± 0.4, *c* = 3.8 ± 0.4, and *d* = 2.5 ± 0.6 kJ mol^–1^ with a correlation coefficient R^2^ > 0.99 (Figure 2a). A similar analysis of Δ*D*(H–C)_rel_ for the parent benzenes yields:^70,71^*a* = 0.7 ± 0.3, *b* = 10.4 ± 0.2, *c* = 0.3 ± 0.2, and *d* = 3.4 ± 0.3 kJ mol^–1^.

Thus, the most important feature of the M–C bond energies is that the parameter *b*, the coefficient for the *ortho* fluorines, is far greater than that for the parent fluorobenzenes. Correlation of the calculated M–C bond energies with the calculated H–C bond energies shows three groups of points according to the number of *ortho* fluorines (0, 1, or 2) for a wide range of metal–ligand systems. The slope of this linear correlation varies from 1.93 for [(CpIr(PH_3_)(C_6_H_5–n_F_n_) to 3.05 for [Cp_2_Zr(H)(C_6_H_5–n_F_n_)]. While these calculations were performed with simplified models and a B3PW91 functional, a set of calculations with full structures and including dispersion (ωB97XD, def2-TZVP) on [Co(PNP)H(Bpin)(C_6_H_4–n_F_n_R)] (PNP is a pincer ligand, R = H, CH_3_, CF_3_, n = 0, 1, 2) yielded a similar correlation with a slope of 2.87 (Figure 2b).^72^ This striking heightened sensitivity to the number of *ortho* fluorines is characteristic of the metal–aryl bonds, although they are far weaker than the H–C bonds. The electronic origin of these effects is analyzed in detail in ref (71), but we emphasize that they are inherent to the M–C bonds. Two features are likely to be particularly important, namely the charge on the *ipso* carbon atom and delocalization of the M–C electron density into the σ*(C–F) orbitals.

As a consequence of the changes in BDEs described above, C–H oxidative addition becomes more favorable thermodynamically as the number of *ortho* fluorine substituents increases. However, for practical purposes, it is important to consider the competing reactions of C–F oxidative addition and hydrodefluorination that are affected by the C–F BDE. DFT calculations show clearly that C–F BDEs of fluorinated benzenes show the opposite trends to C–H BDEs, although the relative values of the parameters *a*–*d* in the analogous equations to eq 4 differ appreciably.^74^ When forming M–F bonds as in C–F oxidative addition, the M–F BDE also comes into play. Calculations show that C–H oxidative addition becomes much more favorable relative to C–F oxidative addition for third-transition-series platinum than for first-row nickel, mainly as a consequence of the stronger Pt–H bond.^62,75^ Within this review, we are targeting C–H functionalization, so these C–F bond-breaking reactions are undesired competitors. We can generalize that C–H oxidative addition is most favorable energetically with *ortho* fluorine substituents and third-row transition metals. It is also worth emphasizing that rationalizations based solely on the properties of the free arenes are inappropriate to such reactions. Consideration of the reactants and products is essential to an understanding of the energetics.

#### 2.1.2 Kinetic Factors

**Scheme 3:**
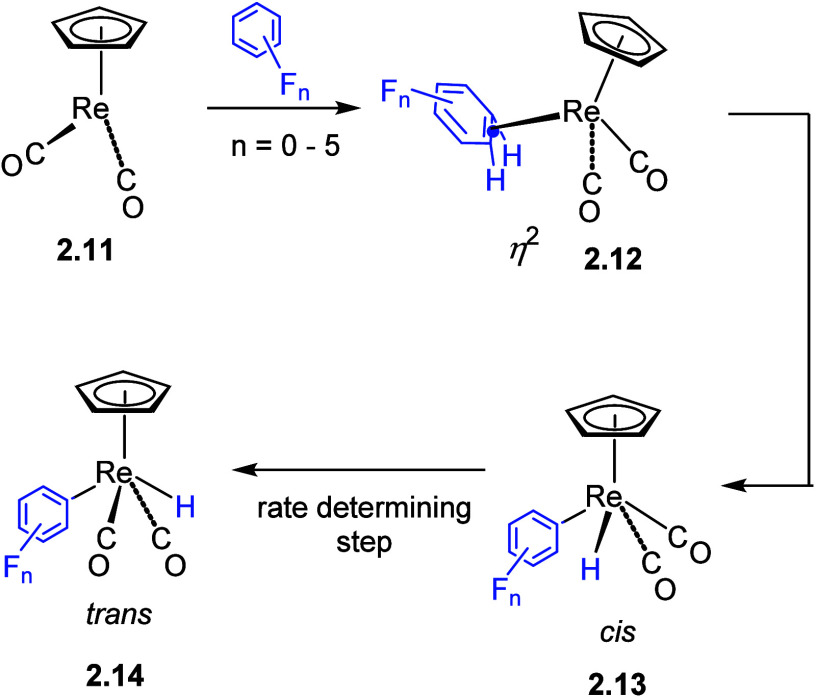
Formation of a Re(η^2^-Arene) Intermediate in the C–H Oxidative Addition of [CpRe(CO)_2_] with Fluoroarenes^70^

The barrier to C–H oxidative addition reactions does not necessarily follow the order of the free energy of the reaction. In the case of reaction of [CpRh(PMe_3_)] with fluoroarenes, the formation of all possible isomers on initial reaction indicates that the kinetics do not follow the thermodynamics, but are influenced by other factors. A closely related set of reactions is observed by photolysis of [(η^5^-C_5_R_5_)Re(CO)_2_L] (R = H or Me, L = CO or N_2_) with fluoroarenes, but this time Re(η^2^-arene) products are observed for some fluoroarenes (Scheme 3).^70,76^ The oxidative addition products are formed with *ortho* selectivity. Notably, photoreaction of [Cp*Re(CO)_2_(N_2_)] with 1,3-C_6_F_2_H_4_ yielded exclusively *trans*-[Cp*Re(CO)_2_(2,6-C_6_F_2_H_3_)(H)] (the isomer of **2.14** with both fluorines *ortho* to Re). DFT analysis of these reactions showed that the strength of the Re–C bonds in the products follows a completely analogous pattern to those of the [CpRh(PMe_3_)] complexes described above. The consequences are different from those of the [CpRh(PMe_3_)] complexes. With no *ortho* fluorines, the reactions stop at η^2^-coordination because oxidative cleavage is energetically unfavorable; with one *ortho* fluorine, a mixture of C–H activation and η^2^-coordination is obtained, and with two *ortho* fluorines, only C–H oxidative addition is observed. Surprisingly, the largest barrier in the reactions arises not from C–H bond cleavage but from *cis*–*trans* isomerization. Nevertheless, the barriers to both C–H oxidative cleavage and *cis*–*trans* isomerization follow the order of the C–H and Re–C bond strengths. Thus, the overall result is a combination of thermodynamics and kinetics working together (Hammond Principle). In Section 4.7.3, we summarize the mechanistic analysis of the nickel phosphine-catalyzed hydrofluoroarylation of alkynes. Although the mechanism is very different, involving a ligand-to-ligand hydrogen transfer (LLHT), the same principles apply. A more detailed analysis of kinetic factors affecting *ortho* selectivity concentrating on pincer complexes has been published by Pabst and Chirik.^64^

**Scheme 4:**
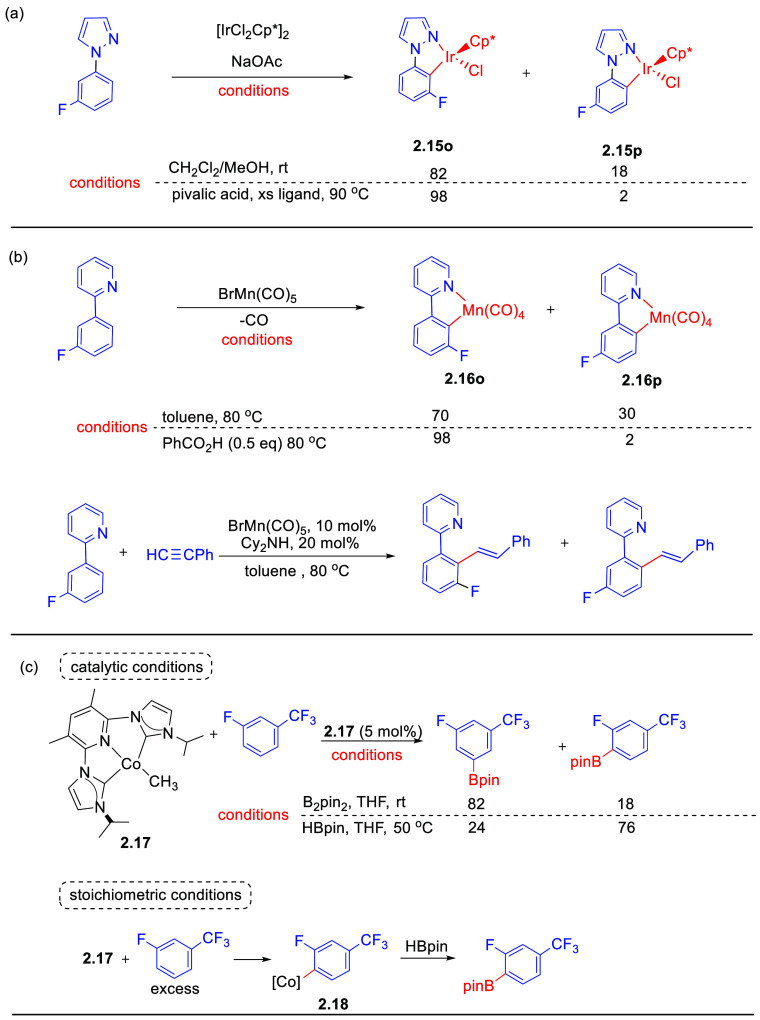
Selected Examples of Switchable Selectivity of Kinetic and Thermodynamic Factors on Fluorinated Substrates in (a) Cyclometalation of 1-Phenylpyrazole at Iridium,^77^ (b) Cyclomanganation of 2-Phenylpyridine,^78^ and (c) Borylation of Fluoroarenes at Cobalt Dicarbene Complexes^79^

Three recent papers provide detailed information about the differences between kinetic and thermodynamic products and how to switch between them. In the first two, the steric effects are modest, but in the third, the catalyst is designed to impose strong constraints. Davies and Macgregor provided crucial evidence for the CMD/AMLA mechanism of C–H functionalization in their early studies of cyclometalation at [Cp*MCl_2_]_2_ (M = Rh, Ir) (see Section 2.2). In revisiting these reactions,^77^ they showed how the product distribution may be manipulated. When 1-phenylpyrazole substituted with fluorine *meta* to the C(phenyl)–N bond is reacted with [Cp*IrCl_2_]_2_ in the presence of NaOAc, there is a choice of positions of C–H activation. Cyclometalated products may be formed with fluorine placed either *ortho* or *para* to the Ir–C bond (**2.15o** and **2.15p**, Scheme 4a). At room temperature, the reaction is irreversible, and the *o*/*p* product ratio, 82:18, reflects the kinetic distribution. However, addition of acid and heating shifts to the thermodynamic ratio, resulting in an increase in the *ortho*-substituted product (*o*/*p* = 98:2). More dramatic changes are observed with bulkier substituents than fluorine. Detailed DFT calculations pinpoint the origin of the kinetic selection.

Reaction of 2-phenylpyridine derivatives with bromomanganese pentacarbonyl results in cyclomanganation. This reaction forms one of the steps in the catalytic alkenylation reaction of phenylpyridines with phenylacetylene in the presence of amine bases (Scheme 4b). As in the previous example, with fluorine placed *meta* to the C–C(pyridine) bond, F may lie either *ortho* or *para* to the newly formed C–Mn bond.^78^ The stoichiometric reaction of this fluorinated phenylpyridine in toluene (80 °C) results in a 70:30 *o*/*p* mixture of cyclomanganated products (**2.16o** and **2.16p**). The products do not interconvert, indicating that this is an irreversible reaction and that the product ratio reflects kinetic selectivity. However, addition of benzoic acid (0.1 equiv) changes the product ratio to 94:6 *o*/*p*. Competition reactions demonstrate that this change depends on intermolecular exchange between free and coordinated 2-phenylpyridine, leading to the thermodynamic product ratio (compare the cobalt system). No significant differences between isomers could be observed in the Mn–C bond lengths, but DFT calculations confirmed the energetic preference for the *ortho* isomer. The full mechanism of the catalytic reaction could be determined by time-resolved IR spectroscopy initiated by photolysis of the cyclomanganated product. This study provides evidence that the cyclomanganation step sets the selectivity for the catalytic alkenylation reaction.

The interplay between steric and kinetic factors is further demonstrated in the cobalt-catalyzed borylation of fluoroarenes with B_2_pin_2_ with a sterically demanding dicarbene ligand in the catalyst **2.17** that controls ligand entry.^79^ The authors use fluoroarenes containing a substituent in one of the *meta* positions, such that activation may occur at the remaining *meta* C–H bond or an *ortho* C–H bond. Under catalytic conditions (5 mol % catalyst, room temperature, THF, 1 equiv B_2_pin_2_), this process exhibits selectivity for *meta* over *ortho* C–H activation for a large range of substrates (Scheme 4c). Paradoxically, stoichiometric reaction of **2.17** with excess 1-fluoro-3-trifluoromethylbenzene in benzene gives the opposite selectivity, generating **2.18** with C–H activation *ortho* to F. Subsequent reaction with HBpin gave the borylated product with Bpin *ortho* to F, demonstrating that the borylation is irreversible. The difference arises because the catalytic reaction conditions impose kinetic selection, whereas the stoichiometric conditions allow reversible C–H activation and thermodynamic selection. Confirmation arises from the reaction of **2.17** with excess 1,3-C_6_F_2_H_4_ at room temperature that gives the cobalt fluoroaryl derivative with both fluorines *meta* to Co after 30 min, but after 24 h, this product isomerizes to the product with both fluorines *ortho* in direct analogy to the observations on CpRh complexes mentioned earlier. Excess fluoroarene is required for isomerization. Thus, kinetics favor *meta*, but thermodynamics favor *ortho*. By doing the catalytic reaction at higher temperature and with HBpin in place of B_2_pin_2_, the isomerization is faster and borylation slower, resulting in *ortho* selectivity (Scheme 4c).

### 2.2 Base-Assisted C–H Bond Activation Reactions

**Scheme 5:**
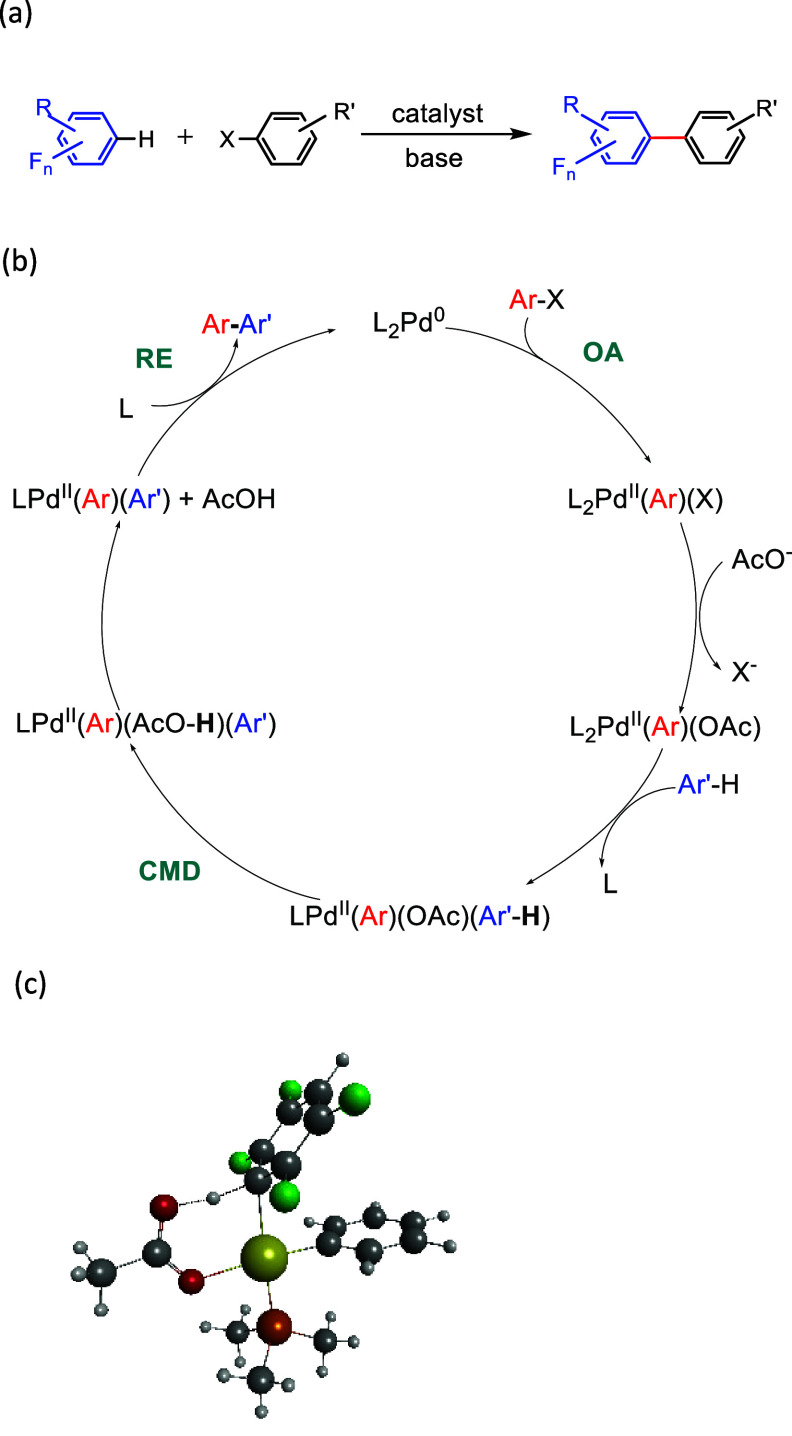
C–H Functionalization Reactions (a) General C–H functionalization reaction for a fluoroarene. (b) General mechanism for a C–H functionalization with a palladium catalyst and acetate base (OA = oxidative addition, RE = reductive elimination).^79^ (c) Calculated structure of the CMD/AMLA transition state for conversion of [Pd(Ar)(OAc)(PMe_3_)(η^2^-1,2,4,5-C_6_F_4_H_2_)] to [Pd(Ar)(HOAc)(PMe_3_)(C_6_F_4_H)].^81^

Base-assisted C–H bond activation reactions typically proceed through the Concerted Metalation Deprotonation (CMD) mechanism, also called Ambiphilic Metal–Ligand Activation (AMLA, Scheme 5a).^63,65^ For example, these reactions enable the conversion of an arene to a biaryl by reaction of the arene with a haloaromatic in the presence of a base and a catalyst—so-called direct arylation. The selectivity for *ortho* fluorines was established in the early studies.^80^ Since then, an enormous range of biaryls have been synthesized according to these principles. The overall reaction is shown in Scheme 5a, and a general mechanism is shown in Scheme 5b, assuming that the catalytically active metal is palladium. This element dominates the following discussion because detailed studies of regioselectivity with fluorine substituents are confined to palladium.

In the CMD/AMLA mechanism, deprotonation occurs via coordination of the base (e.g., a carboxylate or a carbonate) assisted by an agostic interaction between the reacting C–H bond and the metal. This agostic interaction enhances the acidity of the substrate (Scheme 5c). As these reactions involve deprotonation of the fluoroarene by base, the p*K*_a_ of the fluoroarene is relevant to the thermodynamics, but it is not the only factor. It should be recalled that p*K*_a_ depends on solvent and temperature; values are listed in Table 1.^82−85^ Fluorination results in marked decreases in p*K*_a_ with the greatest effect for a fluorine *ortho* to the ionizing hydrogen, as shown for the set of tetrafluorinated benzenes. *Ortho*, *meta*, and *para* fluorine substituents are estimated to reduce the p*K*_a_ by 5.2, 3, and 1.4, respectively.

**Table 1:** p*K*_a_ Values of Fluorinated Benzenes

	Experimental in C_6_H_11_NH_2_ at 34 °C^82^	Experimental in THF at 25 °C^83^	DFT Computational in THF^84^	DFT Computational in DMSO^85^^a^
C_6_F_5_H	25.8 ± 0.2			29.0
1,2,4,5-C_6_F_4_H_2_		28.4 ± 0.2	29.0	23.1
1,2,3,4-C_6_F_4_H_2_	31.5 ± 0.2	32.2 ± 0.2	35.9	30.6
1,3,4,5-C_6_F_4_H_2_		29.9 ± 0.2	30.9	25.0
1,3,5-C_6_F_3_H_3_		33.1 ± 0.2	33.7	31.5
1,2,4-C_6_F_3_H_3_				26.1
1,2,3-C_6_F3H_3_				33.2
1,3-C_6_F_2_H_4_		34.3 ± 0.2		28.7
1,2-C_6_F_2_H_4_	35.0 ± 0.2			33.9
1,4-C_6_F_2_H_4_				40.1
C_6_FH_5_				36.8
C_6_H_6_	43.0 ± 0.2			44.7

a The p*K*_a_ of the most acidic position is listed.

**Figure 3:**
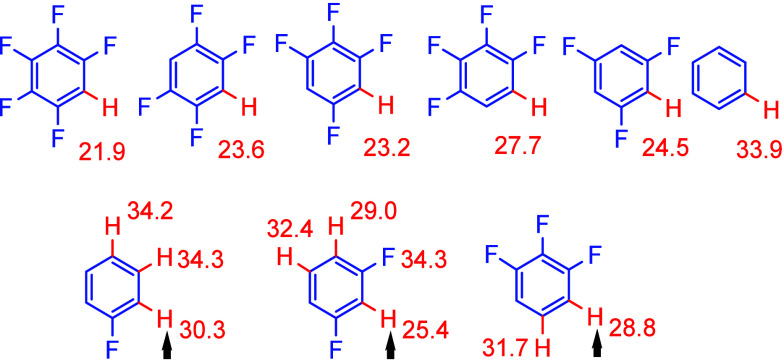
Gibbs free energies of activation (Δ*G*^‡^(298 K) in kcal/mol) of C–H bonds in different fluorinated arenes by [Pd(C_6_H_5_)(PMe_3_)(OAc)] via the CMD/AMLA process. For the three fluorobenzenes below, the arrow shows the position with the lowest barrier.^63^

Three computational studies address the C–H functionalization of fluorobenzenes. Perutz and Eisenstein reported calculations on the complete set of fluorobenzenes for the catalytic cycle in Scheme 5a and addressed regioselectivity.^81^ Gorelsky studied a similar catalytic reaction with a very wide range of substrates and reported data for several fluorobenzenes (see Figure 3).^63^ Ess et al.^86^ reported calculations, with the same catalytic system, on 1,3-difluorobenzene and numerous other nonfluorinated substrates. As the catalytic reaction is irreversible, the selectivity is kinetically determined. Perutz and Eisenstein showed that the CMD/AMLA step is endothermic and divides into three groupings according to the number of *ortho* fluorines, with the lowest barrier for the group with two *ortho* fluorines.^81^ The barrier to the CMD/AMLA reaction correlates with the heterolytic C–H bond energy. However, the order is reversed for the barrier to reductive elimination of the biaryl, with the highest barrier arising for the group of substrates with two *ortho* fluorines. In the overall reaction, the sensitivity to the number of *ortho* fluorines is much greater for the CMD/AMLA step than for the reductive elimination step. Thus, the observed *ortho*-selectivity must arise at the CMD/AMLA step.

Gorelsky’s calculations with [Pd(C_6_H_5_)(PMe_3_)(OAc)] as the catalyst confirm that the lowest barriers to C–H cleavage via the CMD/AMLA reactions are obtained for the substrates with two *ortho* fluorines (barrier 22–25 kcal/mol), a higher barrier with one *ortho* fluorine (27–30 kcal/mol), and a higher barrier still with no *ortho* fluorines (32–34 kcal/mol), consistent with the previous calculations summarized above.^63^ Gorelsky also stressed the important contribution of decreased C–H acidity on fluorine substitution. He noted that fluorine substitution reduces the heterolytic Pd–C bond energies and increases the Pd–C homolytic BDEs.

All authors agree that *ortho*-selectivity arises at the CMD/AMLA step, but the source of selectivity is more controversial. Perutz and Eisenstein stressed the role of Pd–C BDEs as well as C–H acidity (B3PW91 6-31G(d,p)). Gorelsky deduced that the most important factor is the reduced distortion energies of the arene C–H bond *ortho* to fluorine (B3LYP/DZP(Pd), TZVP). Ess et al. (M06/LANL2DZ, 6-31G(d,p)) pinpointed the developing strength of the Pd–C bond in the transition state and showed that this correlates excellently with the activation barrier.^86^

As will be seen in subsequent sections, complexes of metals such as ruthenium have also been employed as catalysts, but we await a full analysis of their *ortho*-selectivity. In most studies, the metal salts used as bases are presumed to act through the anion only, typically a carboxylate, carbonate, or phosphate.^87^ The cations are assumed to have only minor roles such as precipitating the halide salts in the product. It was not until 2016 that it was discovered that this assumption did not apply to silver salts. In several cases, it has now been proven that silver salts catalyze H/D exchange in fluorobenzenes and that Ag(I) can act as the C–H bond activator in C–H functionalization instead of, or in competition with, palladium.^88−98^ Silver carbonate, often used as a base, is highly insoluble in all common solvents, but is solubilized by coordination to phosphines. Dinuclear silver carbonate complexes may be isolated with coordinated phosphine.^94,96^ Moreover, Ag_2_CO_3_ reacts with pentafluorobenzene in the presence of the bulky phosphine XPhos to form isolable mononuclear [Ag(C_6_F_5_)(Xphos)] which reacts with a palladium aryl to form a biaryl below room temperature. This silver complex is also catalytically competent.^96^ It is now evident that a full understanding of these reactions requires an appreciation of silver chemistry. For example, [Ag(C_6_F_5_)(Xphos)] and [{Ag(XPhos)}_2_(CO_3_)] are moderately labile and undergo exchange with free phosphine on a time scale of seconds.^96^ On the other hand, the corresponding triphenylphosphine complexes [Ag(C_6_F_5_)(PPh_3_)_n_] and [{Ag(PPh_3_)_2_}_2_(CO_3_)] are so labile that they defy characterization in solution.^97^ At present, little is known about the regioselectivity of silver-catalyzed reactions or their mechanisms. Whether there are any other metals salts used as additives that have a role in C–H activation has not been established either.

## 3 *Ortho* C–H Borylation of Fluoroarenes

**Scheme 6:**
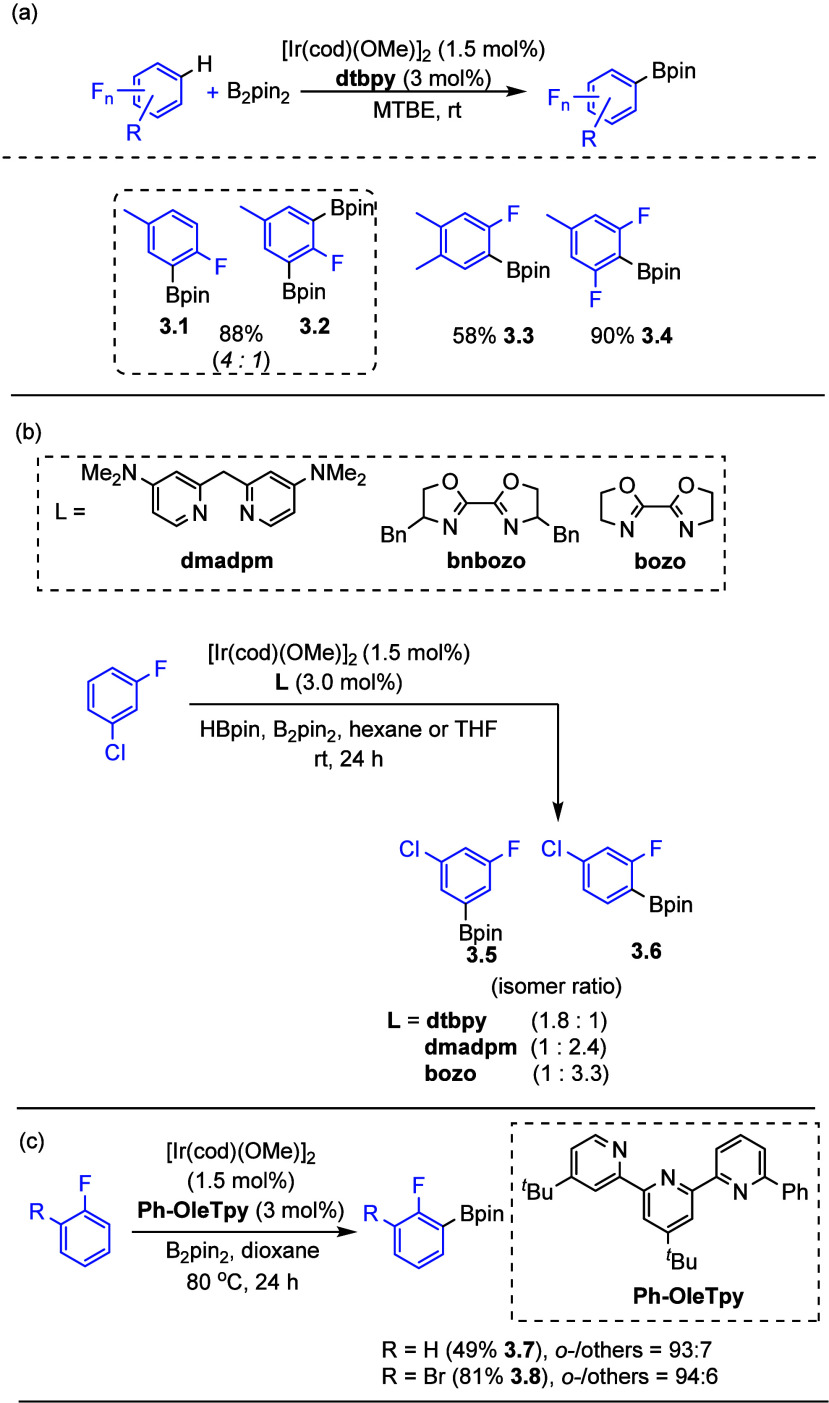
Selected Examples of *ortho*-to-Fluorine Borylation^113,57,120^

Many methods for the introduction of (poly)fluoroarenes into more complex organic molecules are based on (poly)fluoroaryl organometallic reagents. These metals include boron,^99^ zinc,^100,101^ magnesium,^102^ lithium,^103,104^ and silicon.^105^ Most of these species are formed and used in situ, and these transformations are described in the relevant sections below. The significant exception is borylation, as the boronate esters or related derivatives are often isolable. However, it is important to note that electron-deficient fluorinated aryl boronates can be challenging substrates to employ due to rapid protodeboronation issues, and methodologies have been developed to utilize such organoboronates which circumvent this problem.^99,106−112^ The generation of *ortho*-fluoro boronic acid derivatives remains a challenge. Industrially, the classical approach of lithiation and trapping with a borate ester remains the most prevalent method. However, as noted above, the move to more sustainable chemistry necessitates more direct catalytic C–H activation approaches.^53,72,113−118^ In this area, methods using iridium,^57−61,113^ platinum,^54,55,119^ and cobalt^53,68−72^ have come to the forefront. Iridium-catalyzed C–H borylation of fluoroarenes is the most common method, and there are multiple examples in which an *ortho* to fluorine boronate ester can be accessed in this way (**3.1**–**3.4**) (Scheme 6a). Of these, the report by Maleczka Jr. and Smith III et al. (Scheme 6b),^57^ in which the use of classical [Ir(cod)(OMe)]_2_ (cod = 1,5-cyclooctadiene) in combination with less hindered and weaker electron-donating ligands such as 2,2′-bis-2-oxazoline (bozo)-type ligands led to significantly higher *ortho* to fluorine selectivity (**3.5**, **3.6**), is particularly noteworthy and suggests that further advances in ligand design will enhance access to these useful building blocks. Inspired by Maleczka Jr. and Smith III, recently, Ilies et al. reported the use of terpyridine ligand derivative such as 4′,4″-di-*tert*-butyl-3-phenyl-1λ^4^-1,2′:6′,2″-terpyridine (Ph-OleTpy) in combination with [Ir(cod)(OMe)]_2_ as the precatalyst. This method led to the highest selectivity that has ever been reported in terms of iridium-catalyzed C–H borylation *ortho* to fluorine, yielding up to 94% of the *ortho* isomer product (**3.7**–**3.8**) (Scheme 6c).^120^ DFT studies revealed that *N,N,N*-coordinated terpyridine Ir^III^(Bpin)_3_ is predicted to undergo rollover cyclometalation to give cyclometalated *N,N,C*-coordinated terpyridine Ir^III^(Bpin)_2_, which may undergo oxidative addition of fluorobenzene. The DFT barrier for *ortho* C–H oxidative addition is the lowest when compared to the *meta* or *para* pathways.

**Scheme 7:**
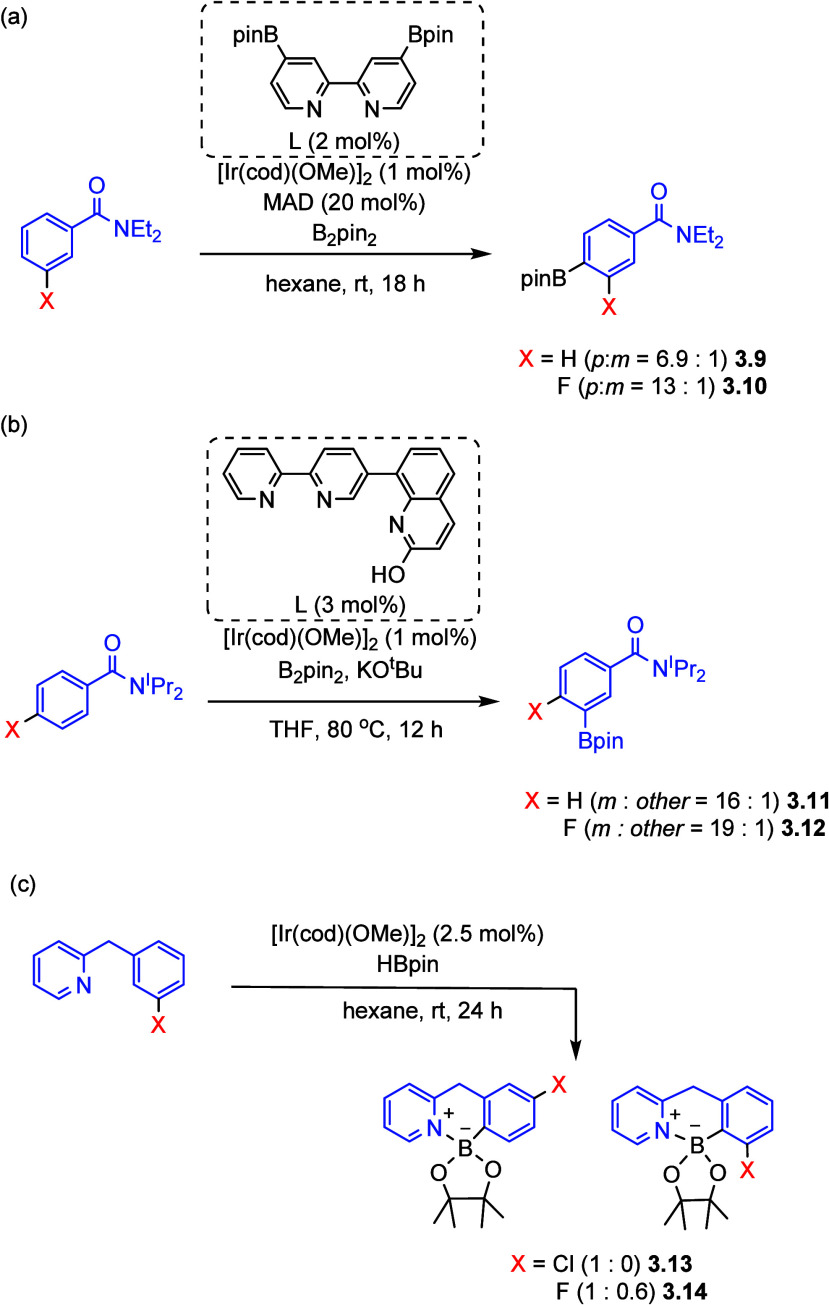
Selected Examples of C–H Borylation Regioselectivity Enhanced by an *ortho* Fluorine Substituent^121−123^ (MAD = methyl aluminum bis(2,6-di-*tert*-butyl-4-methylphenoxide).

In general, the selectivity in iridium-catalyzed C–H borylation, especially with 4,4′-di-*tert*-butyl-2,2′-bipyridine (dtbpy) as a ligand, the most common ligand used for this reaction, is largely driven by steric factors, but there is an underlying electronic influence.^57−61,113^ As a result, *ortho* to fluorine borylation is enabled by the small size and high electronegativity of the fluorine atom, which facilitate the C–H activation and also stabilizes the Ir–C bond. In addition to the reports presented in Scheme 6, this can be observed in other studies in which the presence of a single fluorine atom enhances the rate or selectivity (**3.10**, **3.12**, **3.14**) when compared with the nonfluorinated analogue (**3.9**, **3.11**, **3.13**) (Scheme 7).

**Scheme 8:**
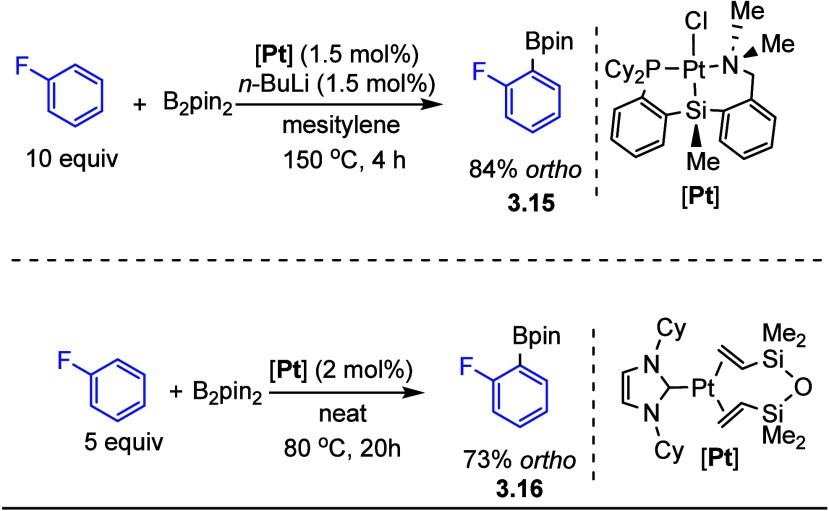
Platinum-Catalyzed C–H Borylation of Fluoroarenes^54,55,119^

Platinum catalysts have also been reported to catalyze C–H borylation as shown in Scheme 8.^54,55,119^ As with the other systems discussed, the introduction of fluorine substituents into a substrate increased the selectivity for and reactivity of an *ortho* to fluorine C–H bond.

**Scheme 9:**
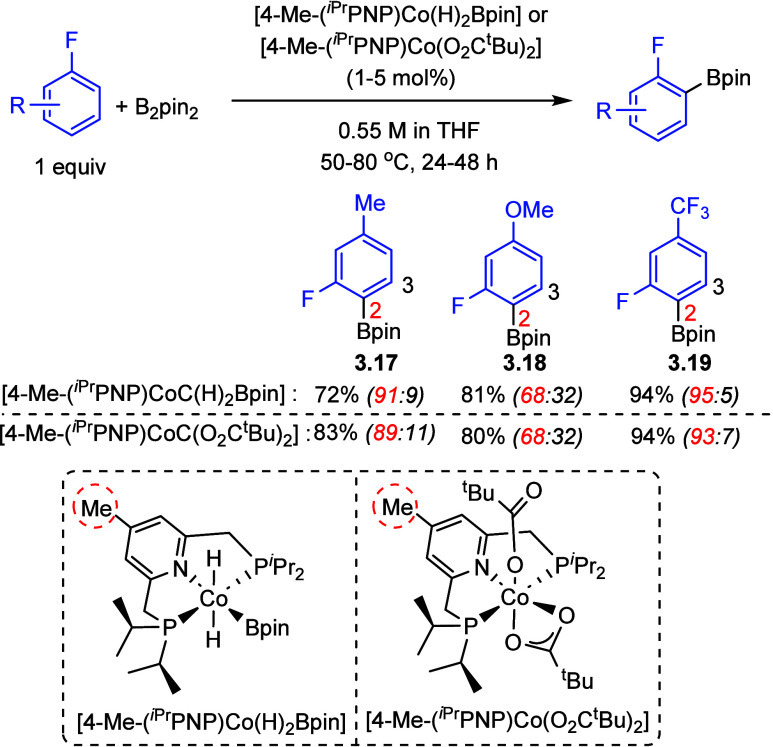
Cobalt-Catalyzed *ortho*-to-Fluorine C–H Borylation^118^

More recently, cobalt-mediated C–H borylation of fluoroarenes has been reported by Chirik et al.^53,67,72,118^ (Scheme 9) (**3.17**–**3.19**), and, here, the driver for *ortho* borylation is a reversible C–H activation and the dominance of the stronger M–C bond (see also Section 2.1, Scheme 2).

## 4 C-C Bond Forming Reactions

### 4.1 Arylation Reactions

Direct C–H arylation of (poly)fluoroarenes was pioneered by Fagnou in 2006.^80^ Subsequently, many reports on the C–H arylation of (poly)fluoroarenes appeared, and a review focused specifically on this arylation approach was published nearly a decade ago by Doucet et al.^124^ Thus, in this Section, we review arylation methods reported in the past decade, and some older reports which were not covered in the review by Doucet et al.

#### 4.1.1 With Aryl Halides

##### 4.1.1.1 Palladium/Silver-Catalyzed C–H Arylation of (Poly)fluoroarenes

**Scheme 10:**
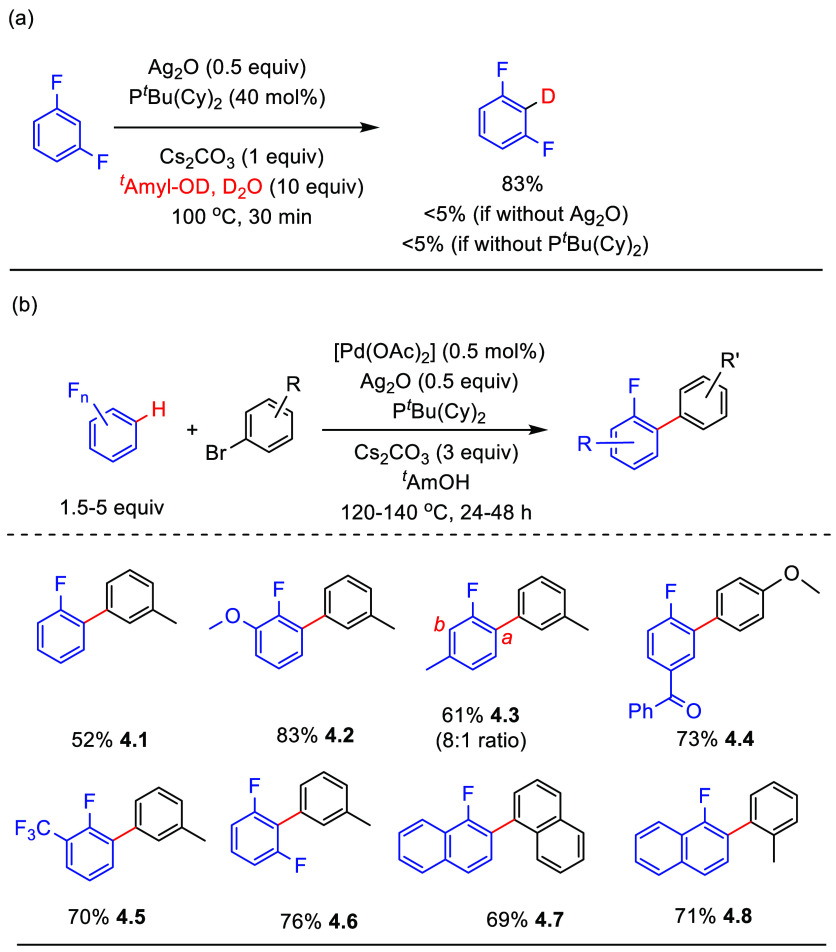
Scope of Palladium-Catalyzed Direct C–H Arylation^93^

In 2013, Larrosa et al.^125^ reported a combination of palladium- and silver-mediated C–H arylation of fluoroarenes that are coordinated with Cr(CO)_3_ through the π-system, with aryl halides as coupling partners. Then, in 2016, Larrosa reported the role of silver in this process.^88^ It was demonstrated that phosphine-ligated silver carbonate could activate the C–H bond of the arene to form aryl silver, which undergoes transmetalation with palladium. A subsequent reductive elimination step yields the biaryl product (for more information about the role of silver in *ortho* to fluorine C–H activation, see Section 2.2). On the other hand, this method suffered from the need to remove the Cr(CO)_3_ unit after the arylation reaction. In 2021, Hartwig et al.^93^ demonstrated the arylation of fluoroarenes using a synergistic combination of palladium and silver as a mediator. Similar to the silver carbonate system by Larrosa,^88^ control experiments by Hartwig also imply that a combination of Ag_2_O with a phosphine ligand, instead of the palladium species, functions to cleave the C–H bond *ortho* to fluorine in the arylation process (Scheme 10a). With the optimized conditions described in Scheme 10b (selected examples), the fluorinated biaryls can be obtained selectively using a combination of 5 mol % of [Pd(OAc)_2_] and P(Cy)_2_(^*t*^Bu) as the catalyst, with Ag_2_O as the C–H activator and Cs_2_CO_3_ as the base, in fair to very good yields (**4.1**–**4.8**). This method can be expanded to employ fluorinated naphthalenes (**4.7** and **4.8**).

##### 4.1.1.2 Palladium/Copper Bimetallic-Catalyzed C–H Arylation of (Poly)fluoroarenes

**Scheme 11:**
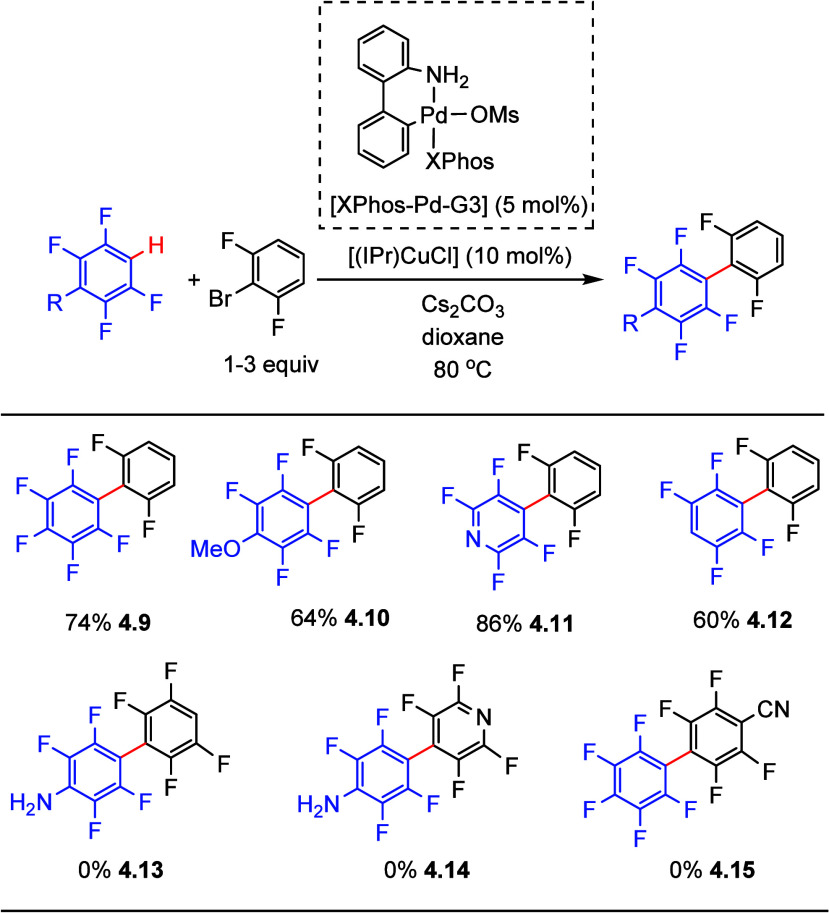
Palladium/Copper Bimetallic-Catalyzed C–H Arylation of Fluoroarenes with Aryl Halides^128^

Copper is known to be able to activate C–H bonds, thus generating copper–carbon bonds in situ.^126^ In 2018, Casares et al. reported that palladium/copper transmetalation of fluorinated aryls is very fast.^127^ This phenomenon was exploited by Casares and Espinet et al. utilizing palladium/copper bimetallic catalytic processes for the C–H arylation of (poly)fluoroarenes with aryl halides.^128^ After optimization, a combination of the precatalysts XPhos-Pd-G3 and [(IPr)CuCl] (IPr = 1,3-bis(2,6-diisopropylphenyl)imidazoline-2-ylidene) was found to generate biaryls, with *ortho* to fluorine substituents on both rings, in moderate to good yields (**4.9**–**4.12**) (selected examples shown in Scheme 11). However, besides the need for the expensive palladium Buchwald precatalyst, the substrate scope is limited to arenes containing 4 and 5 fluorine substituents, i.e., C_6_F_5_H and 1,2,4,5-tetrafluorobenzene derivatives. Notably, substrates bearing −NH_2_ (**4.13**–**4.14**) or (**4.15**) −CN substituents were not viable for this process.

**Scheme 12:**
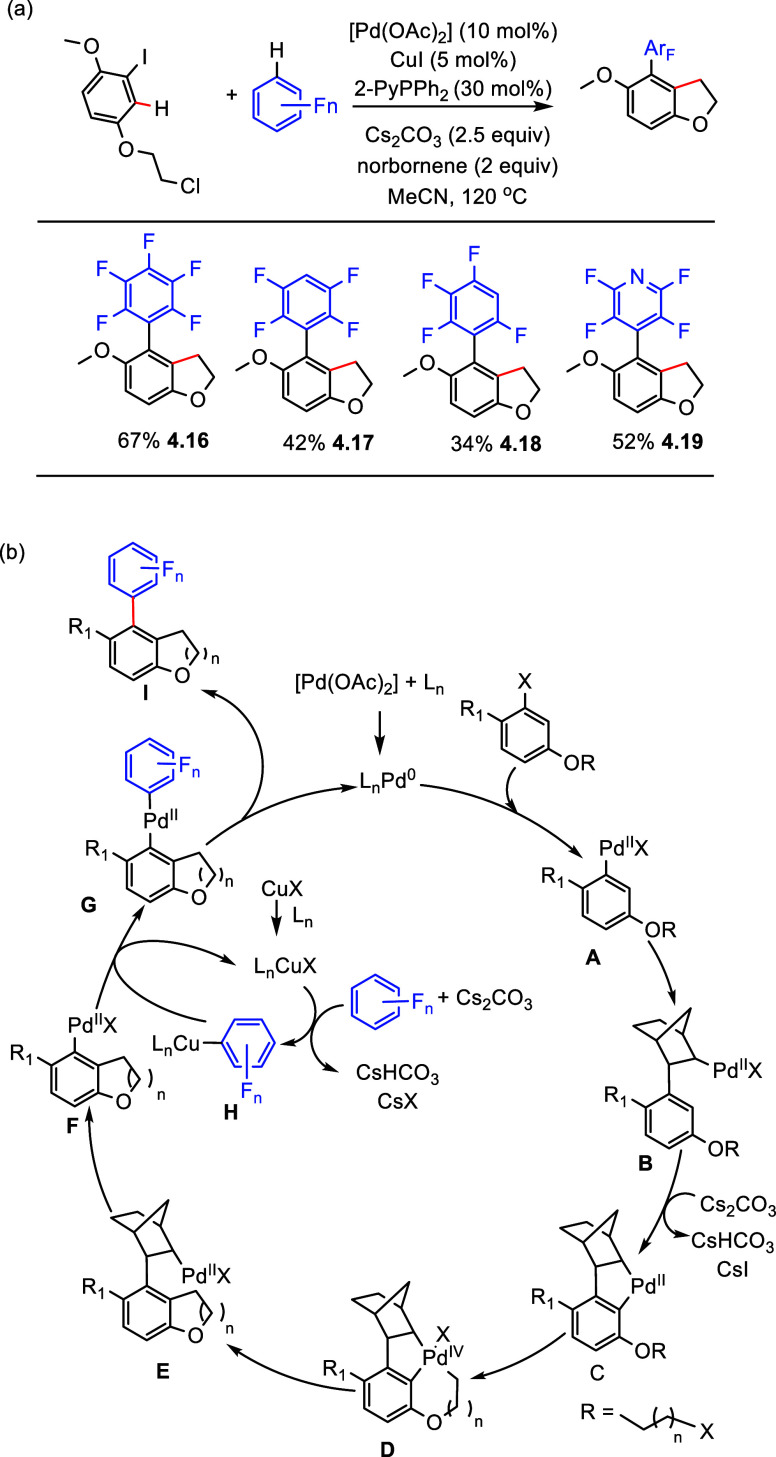
(a) A Cooperative Palladium/Copper-Catalyzed Dual C–H Bond Activation and Coupling of Electron-Deficient (Poly)fluoroarenes with Aryl Halides and (b) Proposed Catalytic Cycle^129^

In 2022, Liang et al. reported a protocol to create (poly)fluoroarene-substituted benzofuran derivatives.^129^ This process was conducted via a Catellani-type reaction^130^ using a combination of [Pd(OAc)_2_] and CuI as the precatalysts, 2-PyPPh_2_ as the ligand, Cs_2_CO_3_ as the base, and norbornene as the *ortho*-directing transient mediator additive in acetonitrile solvent at 120 °C. Under optimized conditions, the corresponding pentafluorophenyl derivative was formed in 67% yield (**4.16**) (Scheme 12a), whereas a lower yield of 42% was obtained for 1,2,3,5-tetrafluorobenzene, attributed to the lower acidity of its C–H bonds (**4**.**18**) (regarding acidity, see also Section 2 and Figure 3). Interestingly, (poly)fluoropyridines were also found to be viable in this process (**4.19**).^129^ No examples of arene substrates with less than four fluorine substituents were employed.

Based on experimental studies, with confirmation from X-ray crystallography, a plausible mechanism was proposed (Scheme 12b). First, oxidative addition of the aryl halide to Pd(0) gives intermediate **A**. Then, insertion of norbornene into intermediate **A** gives intermediate **B**. Next, C–H activation occurred to form an alkylaromatic five-membered palladacycle **C**. Then, oxidative addition of the alkyl halide to palladacycle **C** forms the Pd(IV) complex **D**, which undergoes reductive elimination to give Pd(II) complex **E**. The elimination of norbornene gives the aryl palladium intermediate **F**. At the same time, C–H activation of the (poly)fluoroarene with [L_n_Cu^I^X] in the presence of a base yields aryl copper intermediate **H**. Transmetalation of **H** with **F** gives palladium bis aryl complex **G** and regenerates the [L_n_CuX] species. Finally, reductive elimination from **G** produces the desired cross-coupling product **I** and regenerates the active Pd(0) species.

##### 4.1.1.3 Ruthenium-Catalyzed C–H Arylation of (Poly)fluoroarenes

**Scheme 13:**
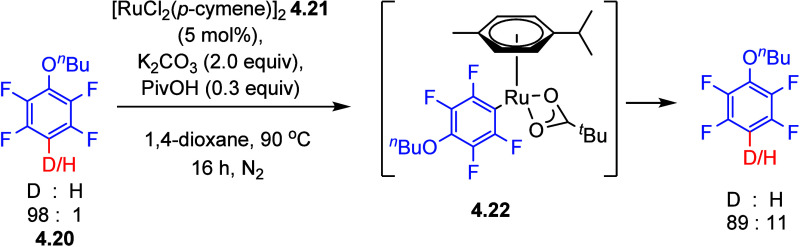
D/H Scrambling via [Ru(fluoroaryl)(OPiv)(π-cymene)] **4.22** Intermediate C–D/C–H Activation^131^

Among the second-row transition metals, developing ruthenium catalysts can represent a significant economic benefit as ruthenium is currently ca. one-third the price of palladium. In 2016, Larossa et al. developed a ruthenium-catalyzed arylation of (poly)fluoroarenes with aryl halides.^131^ This investigation began after they reported that Ru(II) complexes are able to activate a C–H bond that is flanked by two C–F bonds. When deuterated (poly)fluoroarenes **4.20** (98% deuteration) were subjected to the reaction using 5 mol % of [RuCl_2_(p-cymene)]_2_**4.21** with conditions described in Scheme 13, interestingly, 9% of H incorporation was detected. Furthermore, ^1^H and ^19^F NMR analysis showed the formation of intermediate [Ru(fluoroaryl)(OPiv)(p-cymene)] (HOPiv = pivalic acid) **4.22** and, notably, no scrambling occurred in the absence of [RuCl_2_(p-cymene)] **4.21**. Larrosa et al. then optimized the conditions to synthesize [Ru(fluoroaryl)(OPiv)(p-cymene)] **4.22** in very good yields by reacting [Ru(OPiv)_2_(p-cymene)] **4.21** with fluoroarenes in the presence of a stoichiometric amount of Na_2_CO_3_ in 1,4-dioxane.^131^

**Scheme 14:**
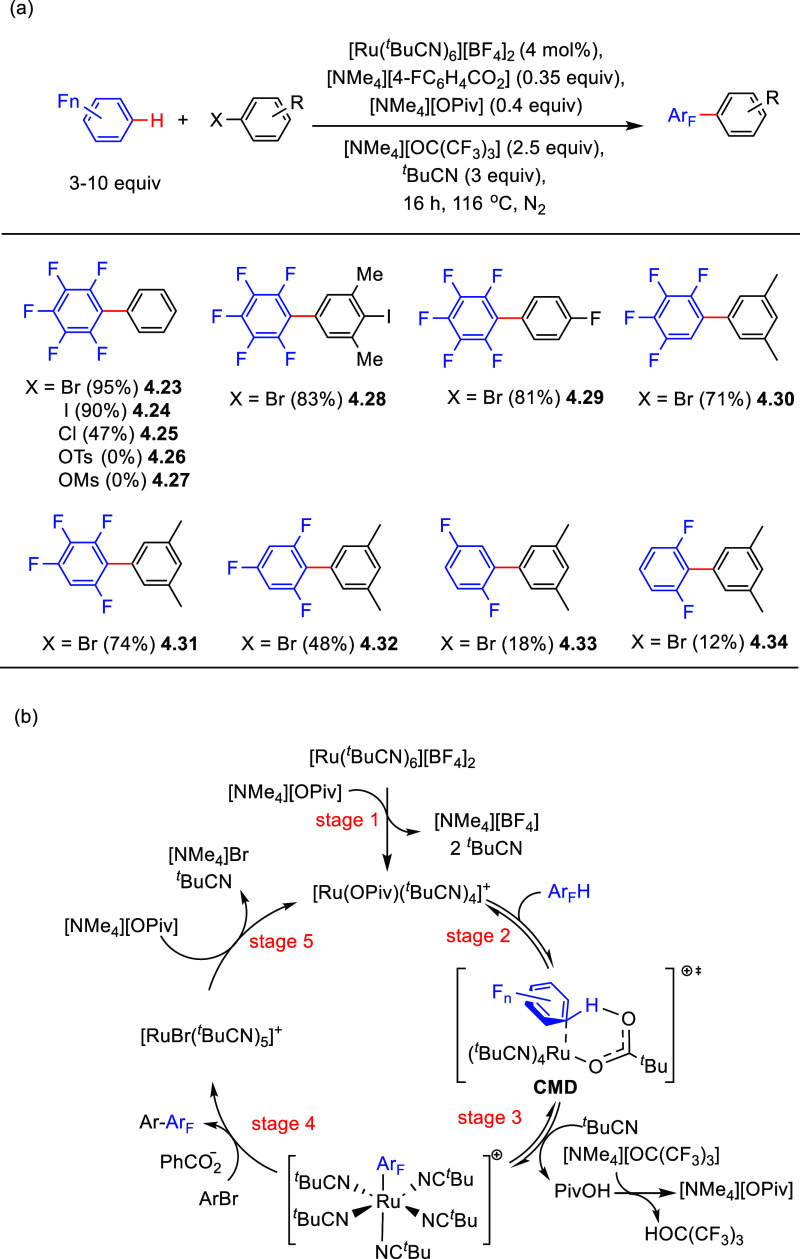
(a) Ruthenium-Catalyzed Arylation of (Poly)fluoroarenes with Aryl Halides and (b) Proposed Catalytic Cycle^131^

Having found that ruthenium catalysts are able to activate the C–H bond of (poly)fluoroarenes, Larrosa and co-workers reported an optimized catalytic system for the C–H arylation of these substrates with aryl halides. Under the conditions described in Scheme 14a with selected examples, the corresponding biaryl products were formed in moderate to excellent yields. While aryl bromides, iodides, and chlorides (**4.23**–**4.25**) worked well, other leaving groups such as OTs (“tosyl”) **4.26** or OMs (“mesyl”) **4.27** failed to give the desired products. Interestingly, if an iodide substituent is sterically protected by two *ortho* substituents, the iodine-containing product was formed in good yield. It should be noted that these conditions did not work well for fluoroarene substrates containing less than three fluorine substituents.

Based on experimental and computational studies, a catalytic mechanism was proposed, as depicted in Scheme 14b.^131^ First, the cationic ruthenium intermediate, [Ru(OPiv)(^*t*^BuCN)_4_]^+^, formed from reaction of the precatalyst [Ru(^*t*^BuCN)_6_][BF_4_]_2_ with [NMe_4_][OPiv] (Scheme 14b, stage 1), activates the C–H bond of the (poly)fluoroarene via a CMD/AMLA process (stages 2 and 3). Next, the Ar_F_–Ru intermediate oxidatively adds the aryl bromide in the presence of the benzoate additive, followed by reductive elimination to form the biaryl product Ar–Ar_F_ (stage 4) and the [RuBr(^*t*^BuCN)_5_]^+^ complex intermediate. Then, halide abstraction regenerates the active cationic ruthenium complex [Ru(OPiv)(^*t*^BuCN)_4_]^+^, and thus closes the catalytic cycle (stage 5).

#### 4.1.2 With Aryl Germanes

**Scheme 15:**
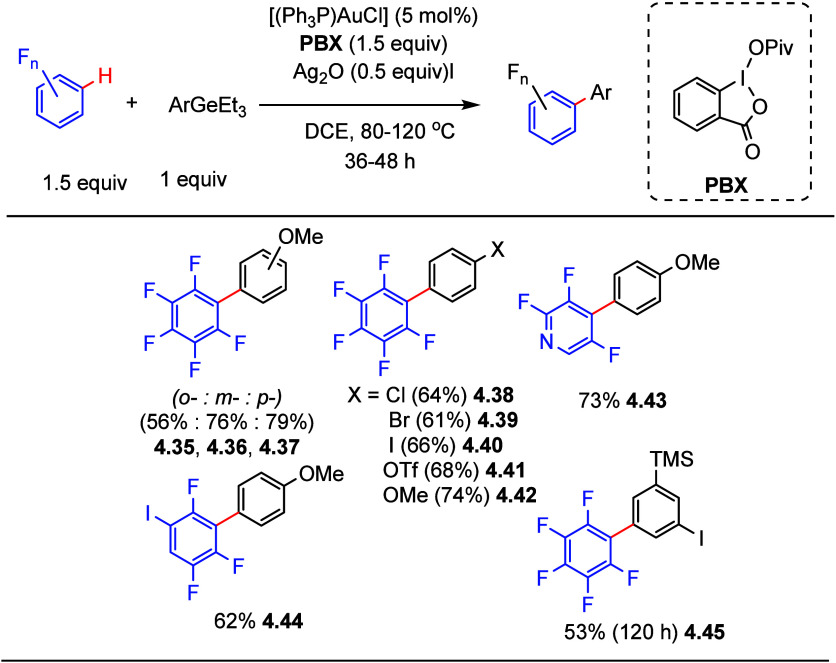
Gold-Catalyzed C–H Arylation of (Poly)fluoroarenes with Aryl Germanes^132^

In the context of gold-catalyzed C–H arylation of (poly)fluoroarenes, elegant work by Larrosa et al. demonstrated that (poly)fluoroarenes can be coupled directly with highly electron-rich arenes via a Au(I)/Au(III) catalytic process (see Section 4.1.3 for details). Inspired by that work, in 2020, Schoenebeck et al.^132^ reported the oxidative coupling of (poly)fluoroarenes with aryl germanes as coupling partners via a gold-catalyzed process. Initial investigations showed that the (poly)fluoroaryl-Au(I) species assisted by Ag(I) promotes C–H activation of C_6_F_5_H, which is then oxidized by an in situ-formed hypervalent iodine oxidant to yield a Au(III) intermediate. Then, addition of 4-methoxyaryl germane to the reaction mixture generated the cross-coupling product C_6_F_5_-aryl in 45% yield after 2 h at 70 °C. After screening of silver sources and different oxidants, it was found that silver oxide, in combination with 1-pivaloyloxy-1,2-benziodoxol-3(1H)-one (PBX), allows the arylation of pentafluorobenzene with 4-methoxyaryl germane and 3-methoxyaryl germane to give 76% (**4.36**) and 79% (**4.37**) yields, respectively (selected examples shown in Scheme 15), whereas the sterically more hindered substrate 2-methoxyaryl germane gave a 56% yield (**4.3**, **5**). Importantly for further functionalization, and similarly to the work reported by Larrosa et al. (see Section 4.1.3), these conditions also tolerated substrates bearing halide and pseudo halide substituents (e.g., Cl, Br, I, OTf, and TMS) (**4.38**–**4.41**, **4.45**) to give the fluorinate biphenyls in moderate yields. Tri-, tetra-, and pentafluoroarenes as well as heteroarenes were also coupled in fair to moderate yields using this process. However, the authors did not employ arene substrates bearing less than three fluorine substituents.

#### 4.1.3 With Unfunctionalized Arenes

**Scheme 16:**
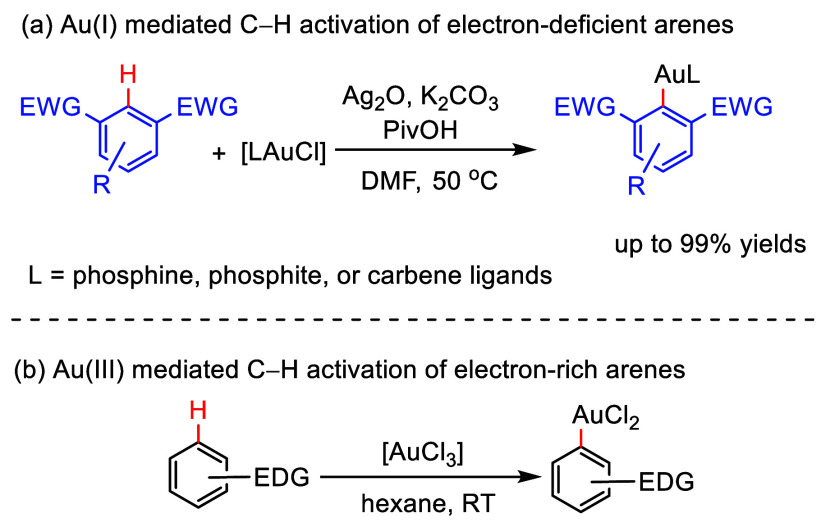
Au(I)- vs. Au(III)-Mediated C–H Activation EWG = electron-withdrawing group, EDG = electron-donating group.^133−135^

**Scheme 17:**
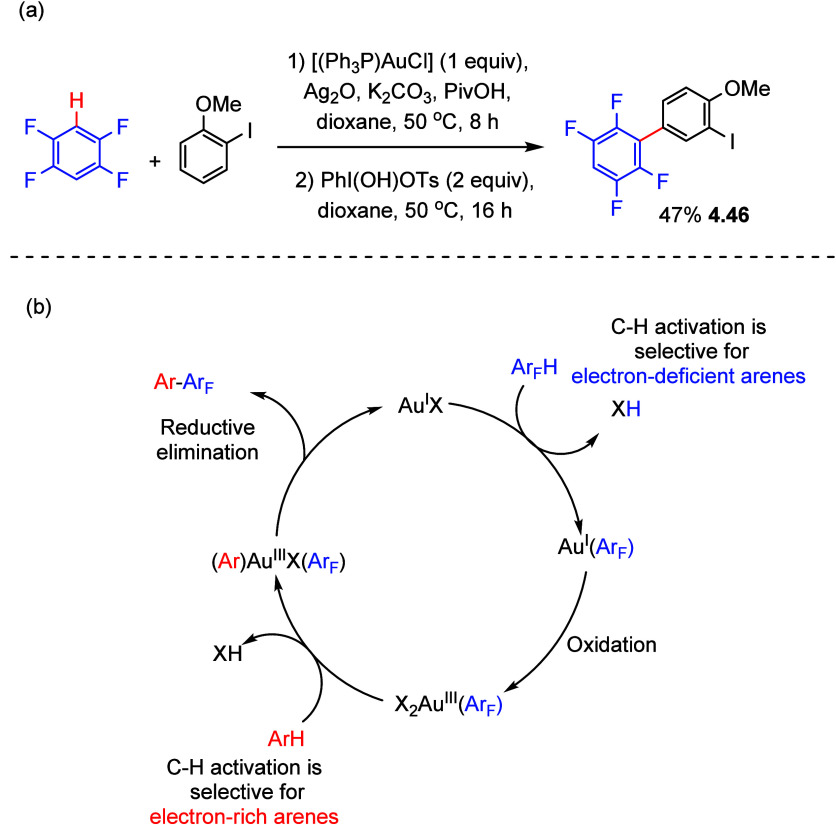
Selective Cross-Coupling via C–H Arylation of (Poly)fluoroarenes with Arenes Containing Electron-Donating Groups^136,137^

Over the past decade, most research has focused on arylation of fluoroarenes with preactivated arenes.^124^ However, using nonpreactivated arenes to arylate fluoroarenes is desirable. In 2010, Larrosa and co-workers reported the ability of Au(I) salts to mediate C–H activation of electron-deficient (poly)fluoroarenes at 50 °C (Scheme 16a). This protocol is compatible with diverse ligands on Au(I), such as alkyl and aryl phosphines, phosphites, and NHCs.^133^ In contrast to Au(I), higher-oxidation-state species such as Au(III) are well-known to mediate C–H activation of electron-rich arenes at room temperature (Scheme 16b).^134,135^ The benefits of Au(I) and Au(III) were combined by Larossa et al. to develop mild synthetic methodologies for C–H arene functionalization via oxidative coupling of electron-deficient (poly)fluoroarenes with electron-rich arenes (Scheme 17). Thus, ArF_n_–H is converted to ArF_n_–Ag, which then reacts with Au(I). Subsequent oxidation to form ArF_n_–Au(III) occurs followed by electrophilic aromatic substitution of electron-rich arenes.

For example, a mixture of 2-iodoanisole (5 equiv) and 1,2,4,5-tetrafluorobenzene (5 equiv), treated with [(Ph_3_P)AuCl] (1 equiv) using dioxane as the solvent, led to the formation of C_6_F_4_H–Au(I) (>99%) and, as ascertained by ^1^H and ^13^P NMR spectroscopic analysis, leaving the 2-iodoanisole untouched. Filtration of the mixture through Celite followed by the addition of PhI(OH)OTs (2 equiv) as the oxidant, to oxidize Au(I) to Au(III), gave selective fluoroarylation of 2-iodoanisole in 47% yield (**4.46**) (Scheme 17a). Again, this process was highly selective as the remaining electron-deficient arene 1,2,4,5-tetrafluorobenzene was left untouched. Notably, the iodo substituent is tolerated throughout this process, and only very small amounts (<1%) of the homocoupling products of both substrates could be detected by GC-MS analysis. This report shows the high selectivity that can be achieved by applying redox selectivity control, as shown in the representative mechanism in Scheme 17b.^137^ However, it must be mentioned that PPh_3_ can function as a reducing agent, reducing the proposed Au(III) species.^138^ This reduction is often inhibited by chelated phosphine ligands which form more stable Au(III) complexes.^139^

**Scheme 18:**
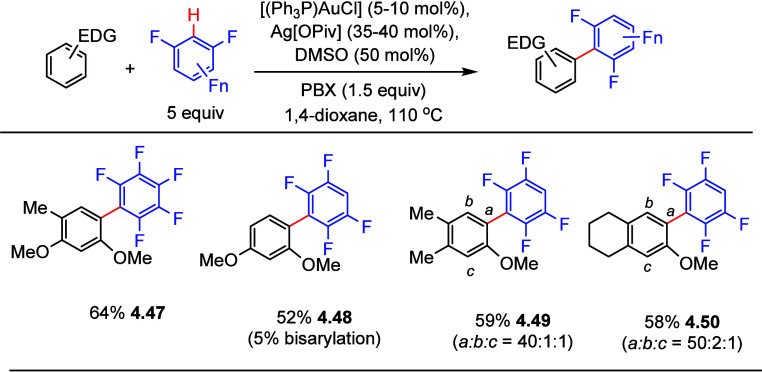
Gold-Catalyzed Cross-Coupling of (Poly)fluoroarenes with Electron-Rich Arenes via Double C–H Activation^137^

Further optimization, using a catalytic amount of [(Ph_3_P)AuCl], was reported in 2015 by Larrosa et al.,^137^ but requiring the more stable oxidant, 1-pivaloyloxy-1,2-benziodoxol-3(1H)-one (PBX), in equimolar amounts to suppress iodination side reactions. Selected examples in Scheme 18 show that (poly)fluoroarylation of electron-rich carbocyclic arenes such as 2,4-dimethoxytoluene, 1,3-dimethoxybenzene, 3,4-dimethylanisole, and a tetrahydronaphthol derivative all gave cross-coupling products in fair yields (**4.47**–**4.50**). Notably, a small amount of bisarylation occurred when 1,2,4,5-tetrafluorobenzene was used as a substrate, due to the reactivity of both C–H bonds (see also Section 2, Figure 3). The authors did not employ electron-deficient arenes bearing less than four fluorine substituents as arylation targets, except for heteroarylation reactions which will be discussed in Section 4.2.1.

### 4.2 Heteroarylation Reactions

#### 4.2.1 With *N*-TIPS-Indole via Double C–H Activation

**Scheme 19:**
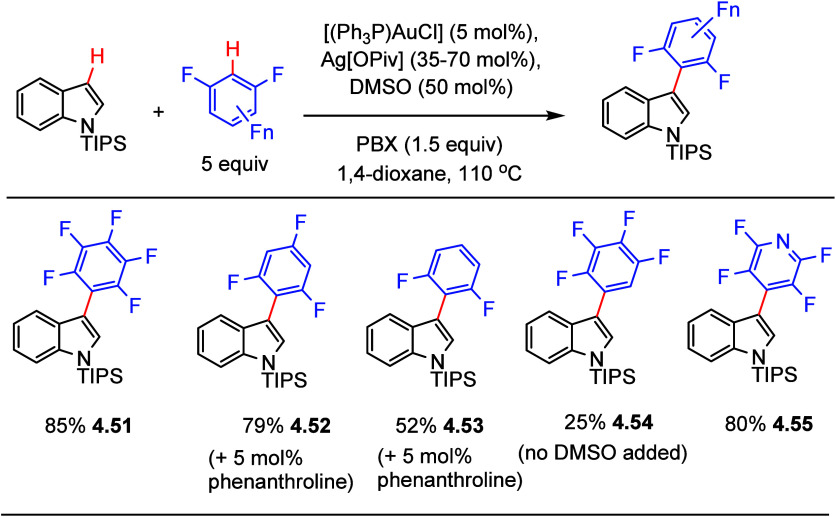
Gold-Catalyzed Cross-Coupling of (Poly)fluoroarenes with Electron-Rich Heteroarenes via Double C–H Activation^137^

It was previously noted that Larossa et al. reported the Au(I/III)-catalyzed oxidative coupling of (poly)fluoroarenes with arenes (see Section 4.1.3). In 2015, he extended the scope of this reaction to include the oxidative coupling with heteroarenes such as N-protected indoles.^137^ Using the optimized conditions already presented in Scheme 18, *N*-TIPS-protected indole can be coupled with various electron-poor (poly)fluoroarenes (Scheme 19). Notably, the presence of two *ortho* fluorines promoting C–H activation is essential for good performance; thus, selected examples in Scheme 19 show that cross-coupling products were obtained in moderate to good yields when employing pentafluorobenzene, 1,3,5-trifluorobenzene, 1,3-difluorobenzene, and, interestingly, 2,3,5,6-tetrafluoropyridine (**4.51**–**4.53**, **4.55**). Conversely, employing arenes containing only one *ortho* fluorine, such as 1,2,3,4-tetrafluorobenzene (25%, **4.54**), fluorobenzene (failed), and 1,4-difluorobenzene (failed), led to poor performance.

#### 4.2.2 With Benzothiazoles via Double C–H Activation

**Scheme 20:**
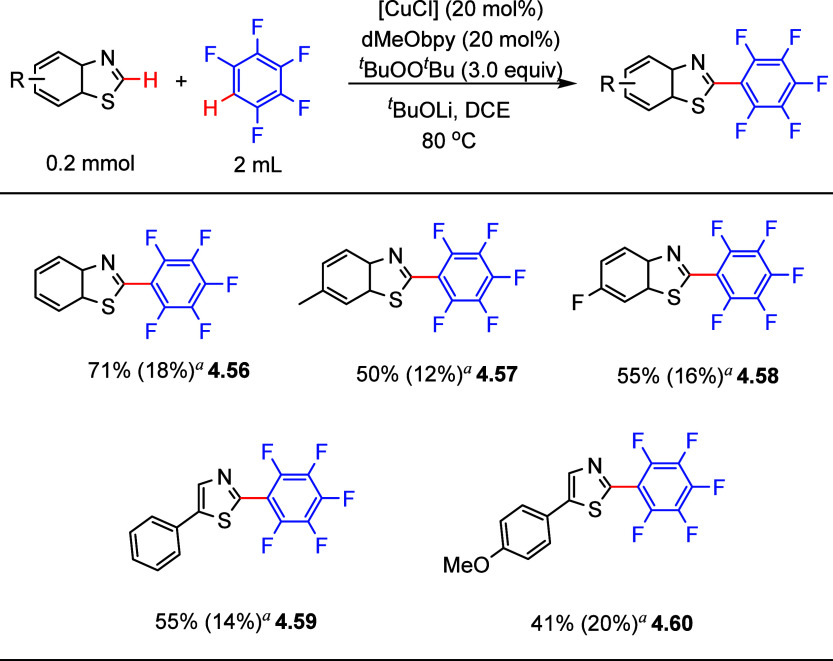
Copper-Catalyzed Dehydrogenative Cross-Coupling of Benzothiazoles with Pentafluorobenzenes^140^ The number in parentheses is the homocoupling product of C_6_F_5_H.

Another approach to transition metal-catalyzed dehydrogenative cross-coupling from two simple C–H bonds was reported by Zhang et al. in 2012.^140^ They demonstrated dehydrogenative cross-coupling of pentafluorobenzene with benzothiazoles using copper(I) chloride as the precatalyst, 4,4′-dimethoxy-2,2′-bipyridine (dMeObpy) as the ligand, and ^*t*^BuOO^*t*^Bu as the oxidant. Selected examples in Scheme 20 show that 2-(poly)fluoroarylthiazole derivatives were isolated in fair to good yields (**4.56**–**4.60**). In general, the yields decreased when benzothiazoles bearing electron-donating or electron-withdrawing substituents were employed. Notably, in addition to the desired cross-coupling product, small amounts of byproduct generated from C_sp^2^_–H homocoupling of pentafluorobenzene was also observed (Scheme 20). However, these conditions were not applied to other types of fluorinated arenes.

### 4.3 Allylation Reactions

#### 4.3.1 With Allylic Carbonates and Halides

**Scheme 21:**
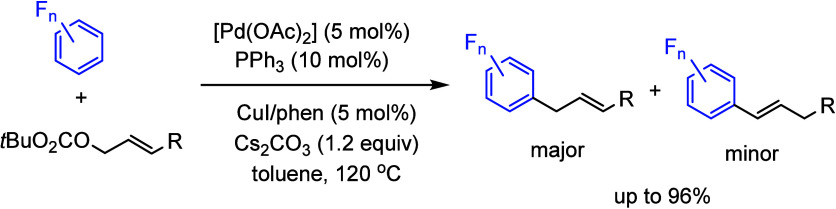
Direct Allylation of (Poly)fluoroarenes with Allylic Carbonates^146^

Allylated arenes are found in many biologically active compounds and natural products.^141,142^ Moreover, the versatility of the allyl group toward functionalization makes this class of compounds very important.^143^ The common Friedel–Crafts allylation reaction is limited in substrate scope to π-electron-rich arenes and moderately electron-deficient ones.^144,145^ In 2011, Zhang et al. reported the use of electron-deficient (poly)fluoroarenes as substrates for direct C–H allylation with allylic carbonates under a [Pd(OAc)_2_]/PPh_3_ catalyst system (Scheme 21).^146^ In this process, the addition of a catalytic amount of CuI/phenanthroline was used to assist the reaction of (poly)fluoroarenes with the palladium catalyst.

**Scheme 22:**
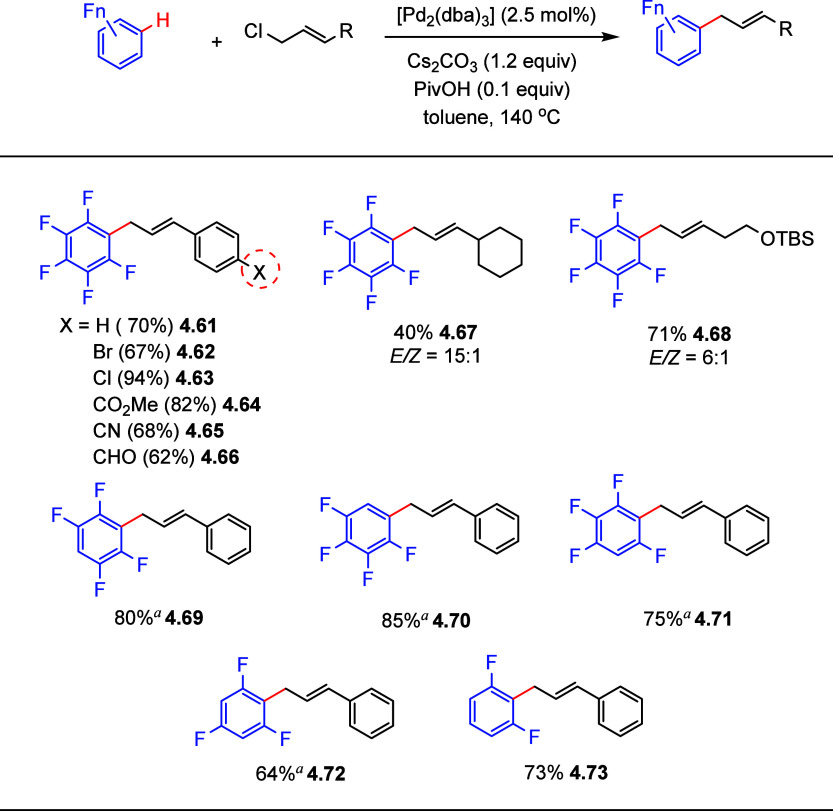
Palladium-Catalyzed Allylation of Fluoroarenes with Allylic Chlorides^147^ Minor disubstituted allylated byproducts were observed by ^19^F NMR.

In 2012, Zhang et al. continued this work on the use of electron-deficient (poly)fluoroarenes as substrates for direct C–H allylation, with allyl halides under ligand-free palladium catalysis (Scheme 22).^147^ Thus, selected examples in Scheme 22 show that reaction of pentafluorobenzene with cinnamyl chloride, under optimized conditions in the absence of a ligand, gave the C–H allylation product in 94% yield, whereas addition of PPh_3_ inhibited the process.

Allylation of pentafluorobenzene with various allyl chlorides containing functional groups on the arene ring such as bromide, chloride, ester, nitrile, and aldehyde, gave fair to excellent yields, with the ratio *E*-3/other isomers >20:1 (**4.62**–**4.66**). Less-reactive aliphatic allylic chlorides gave moderate yields (**4.67**). Arenes containing 2–4 fluorine substituents gave the desired products in fair to moderate yields (**4.69**–**4.73**), with preferential reaction occurring at C–H bonds *ortho* to two fluorines.

**Scheme 23:**
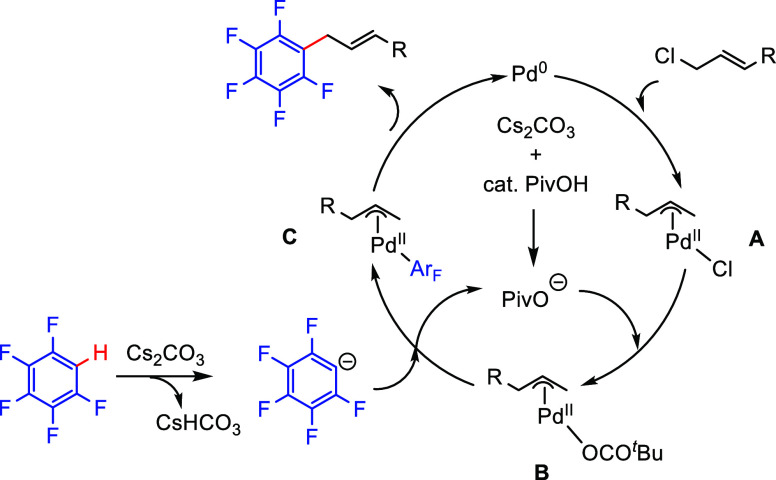
A Plausible Mechanism for the Palladium-Catalyzed C–H Allylation of Fluoroarenes^147^

Based on kinetic isotope studies and H/D-exchange experiments, a mechanism for the palladium-catalyzed C–H allylation reaction was proposed (Scheme 23). The catalytic process begins with oxidative addition of the allyl chloride to Pd(0) to generate [Pd(π-allyl)Cl] (intermediate **A**). This is followed by ligand exchange between pivalate and chloride to generate the (π-allyl) palladium pivalate complex (intermediate **B**). The fluoroarenyl anion, generated by deprotonation with Cs_2_CO_3_, reacts with complex **B** to form [Pd((poly)fluoroaryl)(π-allyl)] **C**, followed by reductive elimination which produces allylic (poly)fluoroarenes and regenerates the active Pd(0) species.

**Scheme 24:**
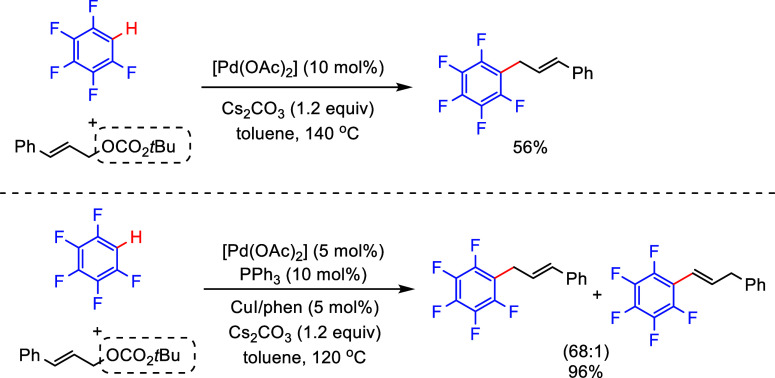
Allylation of (Poly)fluoroarenes with *tert*-Butyl Cinnamyl Carbonate^147,148^

Lower yields were observed when the allylic substrate contained other leaving groups such as *tert*-butyl carbonate, and the desired product was not obtained for the bromide.^147^ However, *tert*-butyl cinnamyl carbonate was a viable substrate for the allylation of (poly)fluoroarenes when using a combination of catalytic amounts of [Pd(OAc)_2_], PPh_3_, and CuI with phenanthroline. This process affords excellent yields with high regioselectivities (Scheme 24).^148^

#### 4.3.2 With *gem*-Difluorinated Cyclopropanes

**Scheme 25:**
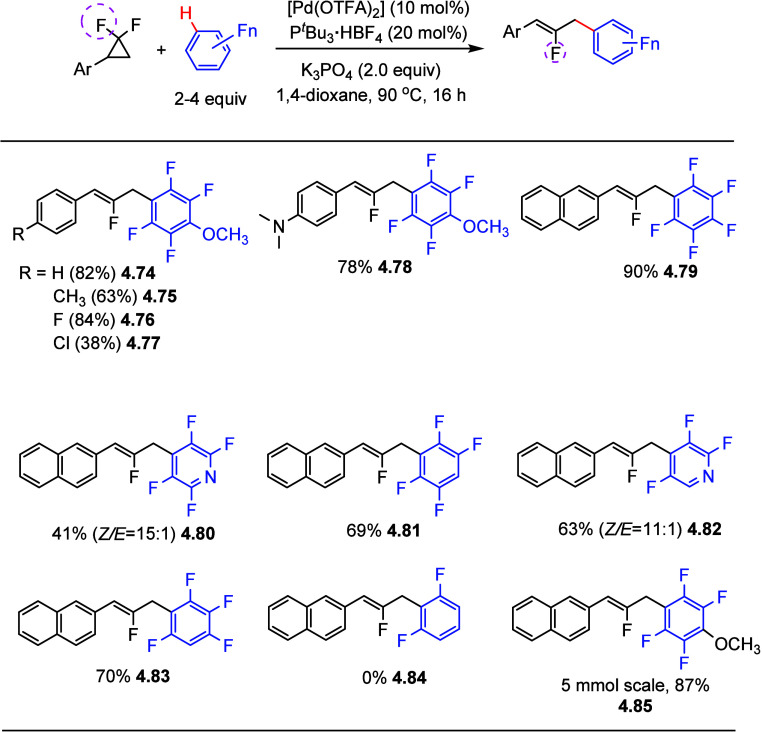
Palladium-Catalyzed C–H Allylation of (Poly)fluoroarenes with *gem*-Difluorinated Cyclopropanes^152^

Recently, transition metal-catalyzed ring-opening of *gem*-difluorinated cyclopropanes was shown to be a promising approach to access monofluoroalkenes. Thus, Wang’s,^149^ Gade’s,^150^ and Xia’s^151^ groups developed protocols for silver-, nickel-, and rhodium-catalyzed ring-opening of *gem*-difluorinated cyclopropanes, respectively. Under Xia’s optimized conditions,^151^ the rhodium-catalyzed C–H allylation of arenes with *gem*-difluorinated cyclopropanes afforded good yields when electron-rich and moderately electron-poor arenes were employed as allylation targets. Notably poorer performance was observed when highly fluorinated arenes such as 1,3-difluorobenzene were used, indicating a remaining challenge. In 2021, Zhou et al. reported the C–H allylation of highly fluorinated benzenes with *gem*-difluorinated cyclopropanes via palladium catalysis.^152^ Initial screening involving 2,3,4,5-tetrafluoroanisole and 2-(2,2-difluorocyclopropyl)naphthalene as coupling partners, showed that 10 mol % of [Pd(OTFA)_2_], 20 mol % of P^*t*^Bu_3_·HBF_4_, and 2.0 equiv of K_3_PO_4_ as a base, in dioxane at 90 °C for 16 h were the best conditions, giving the mono-fluorinated allyl arene product in 95% yield. A survey of substrate scope revealed that *gem*-difluorinated cyclopropanes bearing electron-donating and electron-withdrawing substituents efficiently afforded the desired products in fair to good yields (Scheme 25). However, employing alkyl-substituted *gem*-difluorinated cyclopropanes was unsuccessful. A variety of arenes and heteroarenes containing 3–5 fluorine atoms were tolerated under these conditions, giving allylated products in good to excellent yields (**4.74**–**4.83**), while 1,3-difluorobenzene proved ineffective (**4.84**) (Scheme 25). The process can be scaled up to gram scale (**4.85**).

#### 4.3.3 With Alkynes As Allylic Electrophile Surrogates

**Scheme 26:**
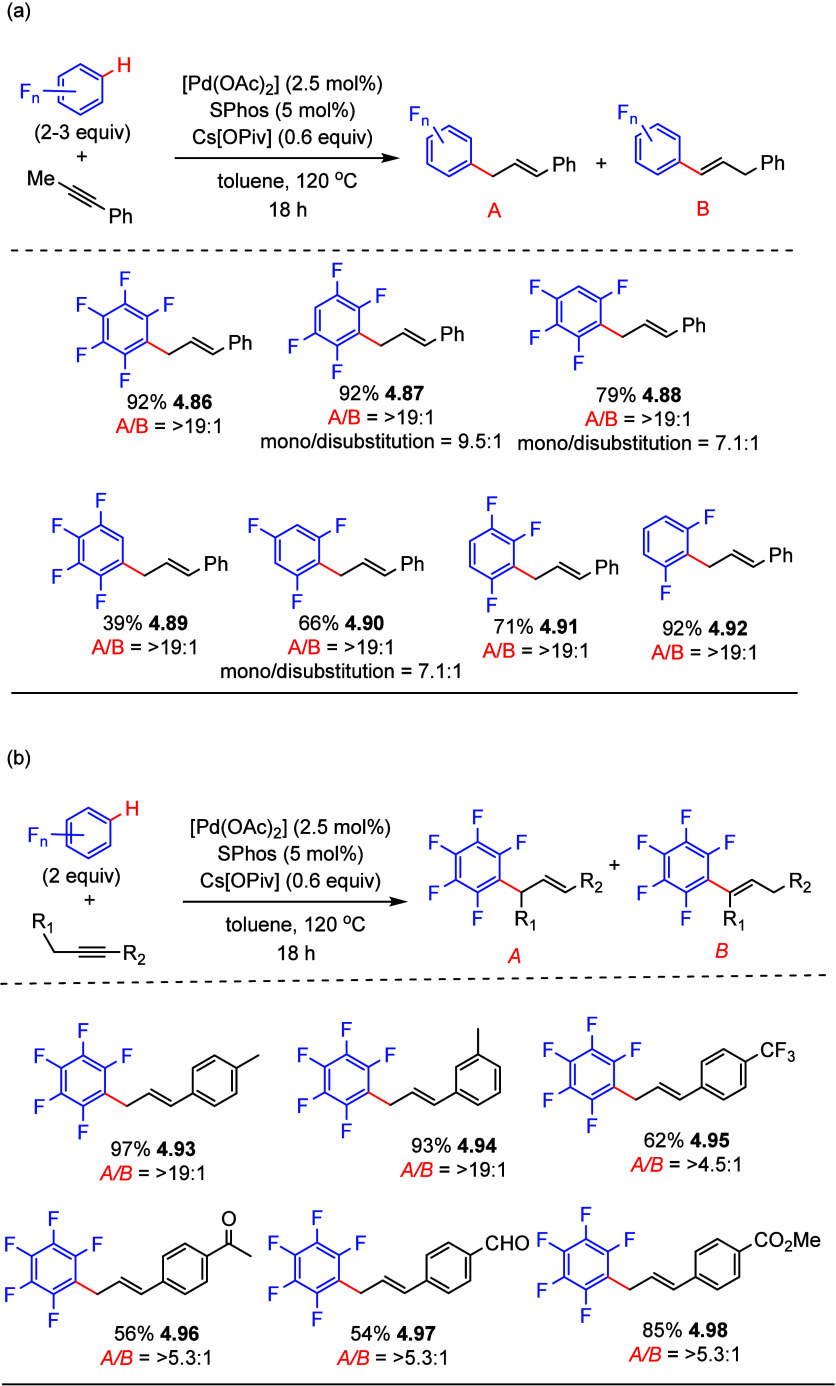
Palladium-Catalyzed Allylation of (Poly)fluoroarenes with (a) 1-Phenyl-1-propyne and (b) Various Alkynes^153^

In 2018, Breit et al. used alkynes as allylic electrophile surrogates in the direct C–H allylation of (poly)fluoroarenes using a palladium catalyst.^153^ It is known that adding a Brønsted acid cocatalyst is necessary to promote alkyne-to-allene isomerization for the allylation reactions. However, as mentioned above, typical palladium-catalyzed allylations of (poly)fluoroarenes required stoichiometric amounts of base to promote deprotonation. Thus, Breit et al. employed the strategy of using a weak base to deprotonate the (poly)fluoroarene. The acid generated can promote the formation of a palladium hydride, which is followed by alkyne-to-allene isomerization to form the desired π-allylpalladium intermediate. With pentafluorobenzene and 1-phenyl-1-propyne as model substrates, the optimized yield was achieved in the presence of 2.5 mol % of [Pd(OAc)_2_], 5 mol % of SPhos, and 0.6 equiv of Cs[OPiv] in toluene at 120 °C for 18 h. The scope of this process was expanded to various arenes bearing two to four fluorine atoms. Selected examples in Scheme 26a show that, in general, activation of a C–H bond *ortho* to two fluorines was favored, giving moderate to very good yields with high regioselectivity (**4.86**–**4.88**, **4.90**–**4.92**). Consistent with this, a low yield was obtained with 1,2,3,4-tetrafluorobenzene, which does not have a C–H bond *ortho* to two fluorines (**4.89**).

**Scheme 27:**
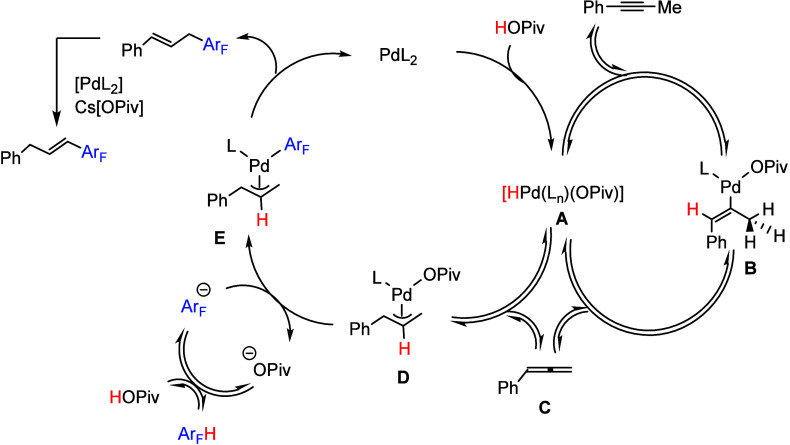
Proposed Mechanism for the Palladium-Catalyzed Allylation of (Poly)fluoroarenes with Alkynes^153^

In addition, selected examples in Scheme 26b show that, in general, internal aryl alkynes containing electron-donating substituents gave excellent yields and high regioselectivities (**4.93**,**4.94**), whereas those with electron-withdrawing substituents gave good yields and moderate regioselectivities (**4.95**–**4.98**). Based on control experiments, H/D exchange experiments, and isotope-labeling experiments, the mechanistic pathway shown in Scheme 27 was proposed. First, deprotonation of pentafluorobenzene with Cs[OPiv] forms the pentafluorobenzene anion and HOPiv. Then, HOPiv oxidizes the palladium catalyst to form the palladium hydride complex **A** (Scheme 27). *syn*-Migratory insertion of the alkyne into the Pd–H bond gives intermediate **B**, and β-hydride elimination gives phenyl allene **C** and reforms palladium hydride complex **A**. In a separate cycle, migratory insertion of **C** into Pd–H in **A** generates π-allylpalladium intermediate **D**. The reaction of **D** with deprotonated pentafluorobenzene affords complex **E**, and the reductive elimination generates the allylation product and regenerates the active palladium catalyst.

#### 4.3.4 With Internal Secondary Allylic Phosphates

**Scheme 28:**
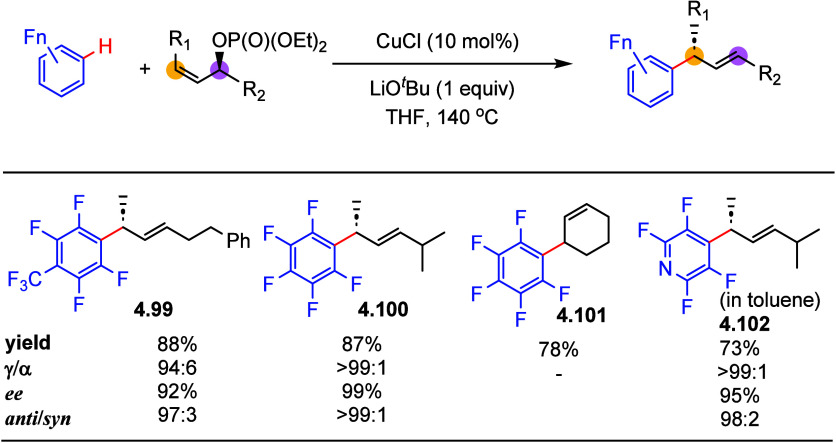
Copper-Catalyzed Allylic Alkylation of (Poly)fluoroarenes^156^

In 2010, Sawamura reported the copper-catalyzed allyl–aryl coupling of aryl boronates with (*Z*)-acyclic or cyclic allylic phosphates.^154^ Inspired by Daugulis and co-workers, who pioneered the functionalization of electron-deficient arenes using copper catalysis,^155^ in 2012, Sawamura applied their previously developed copper-catalyzed allyl–aryl coupling conditions to react fluoroarenes with secondary allylic phosphates.^156^ As described in Scheme 28, selected examples show that this copper-catalyzed reaction of pentafluorobenzene or tetrafluoroarene derivatives with enantio-enriched phosphates gave good to excellent yields of products with excellent regioselectivity and 1,3-*anti* stereochemistry (**4.99**–**4.102**), whereas arenes containing less than four fluorines were not tested.

**Scheme 29:**
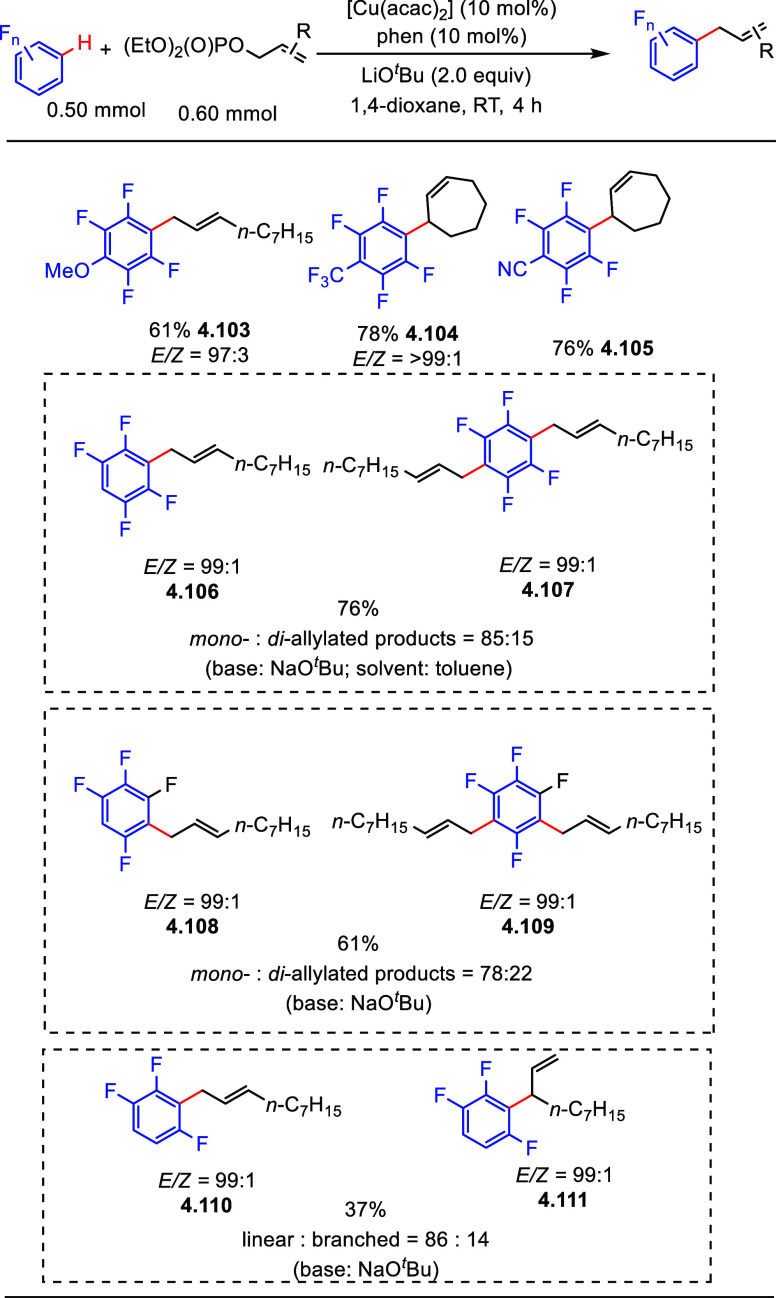
Copper-Catalyzed Allylation of (Poly)fluoroarenes with Allyl Phosphates^157^

A similar allylation method was developed by Miura et al. in 2011, using a combination of [Cu(acac)_2_] and phenanthroline as the catalyst for the direct C–H allylation of (poly)fluoroarenes with allyl phosphates.^156^ Selected examples in Scheme 29 show that allylation of an array of (poly)fluoroarenes was successful, including tetrafluorobenzenes bearing diverse substituents, e.g., methoxy, trifluoromethyl, and cyano groups (**4.103**–**4.105**). A cyclic allyl phosphate was also tolerated (**4.104**, **4.105**). Noting that 1,2,4,5-tetrafluorobenzene gave mono- and di-allylated products (**4.106**, **4.107**) in a ratio of 85:15 under palladium catalysis (Scheme 29), a similar result was found when employing 1,2,3,5-tetrafluorobenzene in the current reaction (**4.108**, **4.109**). Notably, 1,2,4-trifluorobenzene reacted differently: 1) the yield was low; 2) the reaction occurred exclusively at C-2, *ortho* to two fluorines (**4.110**), and no product from allylation at the 5- or 6-position was observed; 3) a significant amount of branched isomer was obtained (**4.111**). Fluoroarenes containing less than three fluorine atoms did not react with allyl phosphates, suggesting that the reactivity and selectivity of this allylation depends predominantly on the acidity of the C–H bond.

#### 4.3.5 With Alkyl-, Aryl-, and Heteroaryl Alkenes

**Scheme 30:**
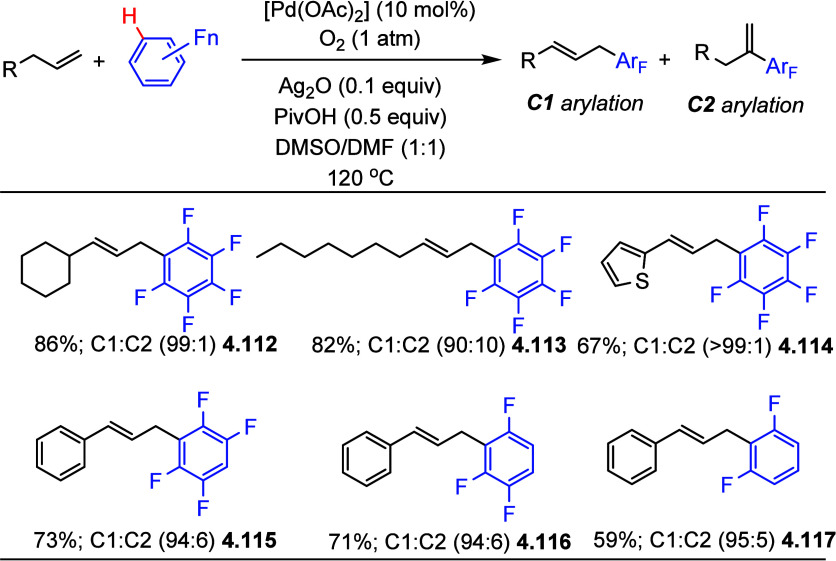
Allylation of (Poly)fluoroarenes with Alkyl-, Aryl-, and Heteroaryl Alkenes^158^

The Tsuji–Trost-type reaction protocols for allylation as reported by Zhang et al. required various allylic chlorides for the (poly)fluoroarylation process (see Section 4.3.1).^147^ However, in 2014, Jiang et al.^158^ reported the oxidative allylation of (poly)fluoroarenes with alkenes via dual C_sp^2^_–H (alkene) and C_sp^2^_–H (fluoroarene) activation. As both substrates are nucleophiles, the reaction is predicted to go via oxidative coupling; thus, an oxidant is needed. Using ligand-free [Pd(OAc)_2_] as the catalyst under an oxygen atmosphere (1 atm) in the presence of 0.1 equiv of Ag_2_O and 0.5 equiv of PivOH, the products were formed in fair to good yields with high regioselectivities. As summarized in Scheme 30, selected examples show that terminal alkyl alkenes, aryl-, and heteroaryl-substituted propenes generated the corresponding linear (*E*)-allylpentafluorobenzene derivatives in high yields with high regioselectivities (**4.112**–**4.114**). The scope of (poly)fluoroarenes was expanded, and the reaction worked well as long as the arene C–H bond was flanked by two C–F bonds (**4.115**−**4.117**).

**Scheme 31:**
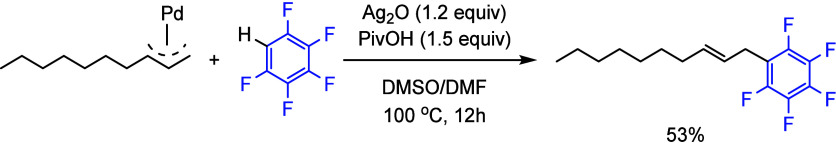
Stoichiometric Reaction of an Allyl-Pd Complex with C_6_F_5_H^158^

The stoichiometric reaction of a π-allylpalladium complex with pentafluorobenzene gave the desired product only in the presence of Ag_2_O and PivOH (Scheme 31), indicating that the silver salt plays a pivotal role in the catalytic process.

**Scheme 32:**
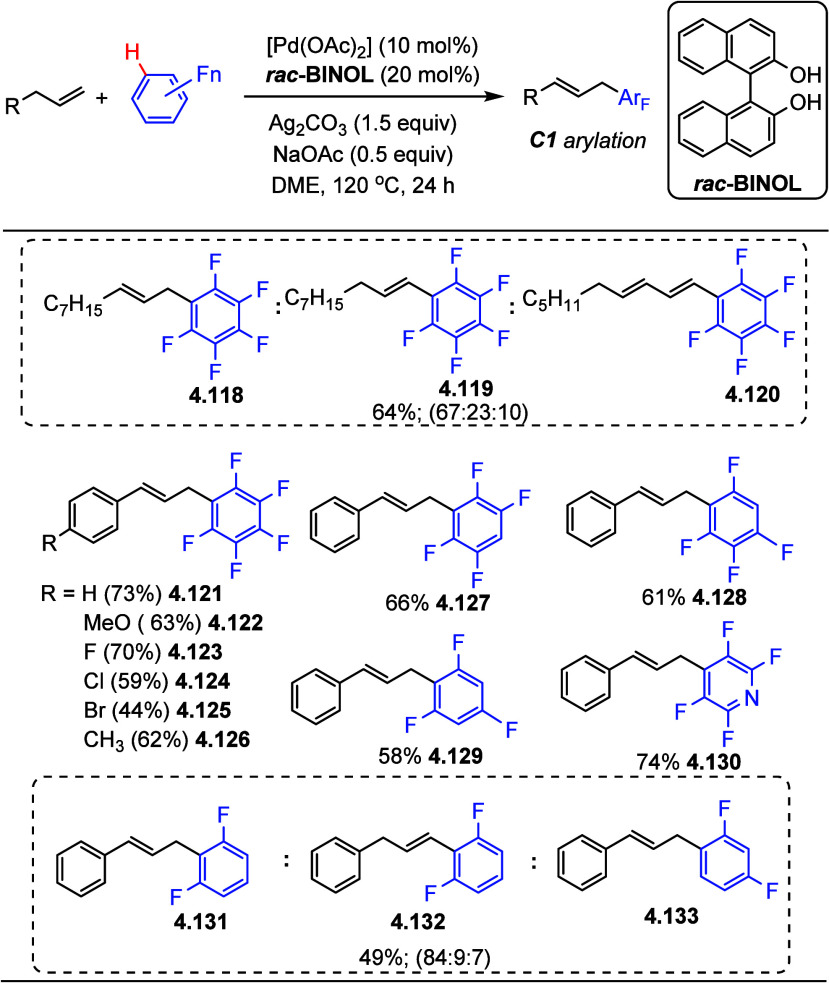
Palladium-Catalyzed Allylic C–H Arylation with (Poly)fluoroarenes^159^

In 2014, Yang et al.^159^ used 1,1′-bi-2-naphthol (*rac*-BINOL) as the ancillary ligand and [Pd(OAc)_2_] as the precatalyst, and a stochiometric amount of an oxidant such as Ag_2_CO_3_. Selected examples in Scheme 32 show that the allylation of (poly)fluoroarenes worked well for allylbenzenes containing electron-donating (−CH_3_**4.126**, −OCH_3_**4.122**) or -withdrawing groups (−F **4.123**, −Cl **4.124**, −Br **4.125**). Substrates containing various numbers of fluorine atoms on the arene and heteroarene ring also resulted in fair to good yields with good regioselectivities (**4.127**–**4.130**). Small amounts of byproducts (but still with good regioselectivity) were observed when substrates such as aliphatic alkenes or 1,3-difluorobenzene were employed (**4.131**–**4.133**).

#### 4.3.6 With Allylic Pivalates

**Scheme 33:**
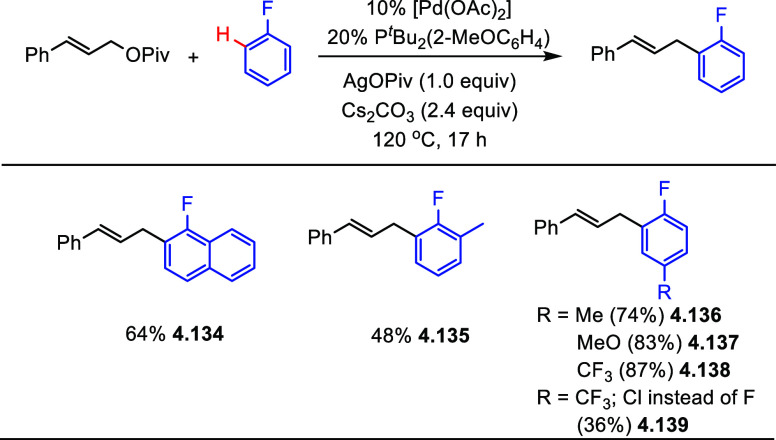
Scope of the Palladium-Catalyzed C–H Allylation of Monofluoroarenes with Cinnamyl Pivalate^90^

Most direct C–H allylations mentioned above (Sections 4.3.1–4.3.5) require a large number of fluorine substituents on the arene ring. Thus, C–H allylation of arenes that are less electron-poor was challenging. In 2016, Hartwig et al.^90^ reported that a combination of [Pd(OAc)_2_] with P*^t^*Bu_2_(2-MeOC_6_H_4_) catalyzes the direct C–H allylation of monofluorobenzenes with allylic pivalates in the presence of Ag(I) additive to give linear (*E*)-allylated arenes. Under the optimized conditions described in Scheme 33, selected examples show that the allylation products can be obtained in fair to good yields (**4.134**–**4.139**).

**Scheme 34:**
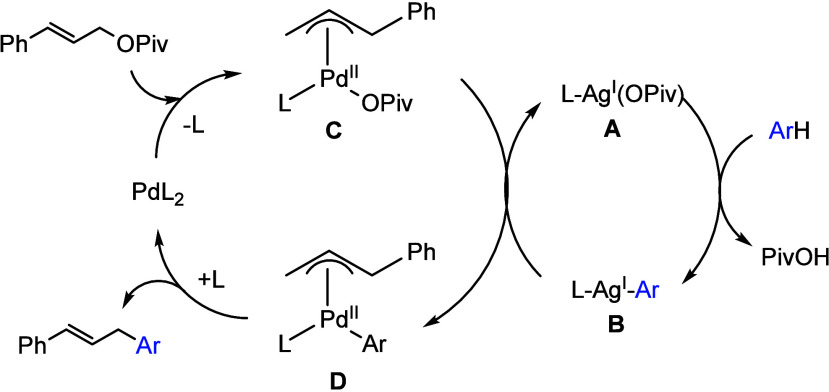
Proposed Mechanism of Palladium-Catalyzed and Silver-Mediated Direct Allylation of Arenes with Allylic Pivalates^90^

Mechanistic H/D exchange studies showed that reaction in the presence of Ag(OPiv) and P*^t^*Bu_2_(2-MeOC_6_H_4_) but in the absence of [Pd(OAc)_2_] gave 67% deuterium incorporation at the position *ortho* to fluorine (for the role of silver in C–H activation, see Section 2.2). This is in line with the selectivity of the allylation products, suggesting that the CMD/AMLA process occurs at the most acidic C–H bond. Based on data from kinetic, NMR, and isotopic labeling studies, Hartwig et al. proposed the catalytic cycle shown in Scheme 34. A pivalate-assisted CMD/AMLA mechanism occurs between L-Ag(OPiv) (**A**) and an arene to give aryl silver intermediate (**B**). In parallel, Pd(0) undergoes oxidative addition with the allylic ester to give allyl palladium species (**C**) followed by transmetalation with aryl silver to generate allyl palladium aryl (**D**). Finally, reductive elimination closes the catalytic cycle to give the allylarene and Pd(0) species.

### 4.4 Carbonylation Reactions with Aryl Halides

**Scheme 35:**
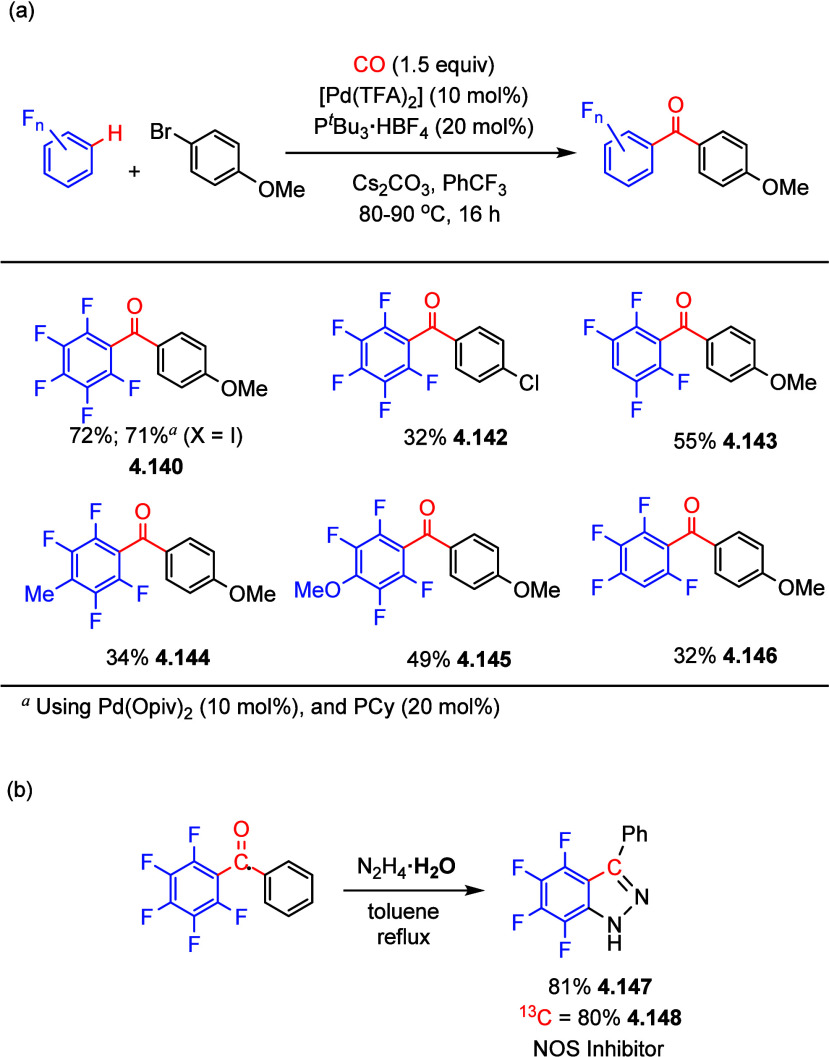
(a) Palladium-Catalyzed Carbonylative Cross-Coupling of 4-Bromoanisole with Various (Poly)fluoroarenes and (b) Synthesis of a ^13^C-Labelled NOS Inhibitor^162^

Arenes containing carbonyl substituents are important motifs that have found many applications in medicinal chemistry. In 2000, Larock and Campo reported carbonylative couplings via intramolecular C_sp^2^_–H activation by palladium.^160^ In 2013, Beller et al.^161^ reported ruthenium-catalyzed C_sp^2^_–H carbonylation in the presence of a pyridine substituent, with the N atom as a directing group. Skrydstrup et al. then speculated that the acidity of (poly)fluoroarenes can make them viable substrates for direct C–H carbonylation, and reported, in 2015, the palladium-catalyzed C–H carbonylation of (poly)fluoroarenes with aryl and heteroaryl bromides in the presence of a stoichiometric amount (1.5 equiv) of CO.^162^ Under the optimized reaction conditions described in Scheme 35, selected examples show that arenes with 4 or 5 fluorine substituents proved to be suitable for carbonylative coupling with aryl bromides and iodides giving fair to moderate yields of diarylketones (**4.140**–**4.146**). The behavior of arenes containing less than 4 fluorine atoms was not reported. Notably, the corresponding aryl iodides were viable under different reaction conditions, and, interestingly, 4-bromochlorobenzene (**4.141**) gave a poor yield with the chloro-substituent untouched (**4.142**). This methodology was used to synthesize biologically relevant compounds such as the potent nitric oxide synthase (NOS) inhibitor in good yield **4.147**, including a carbon-13 labeled version of this compound **4.148** (Scheme 35).

### 4.5 Alkynylation Reactions with Terminal Alkynes

**Scheme 36:**
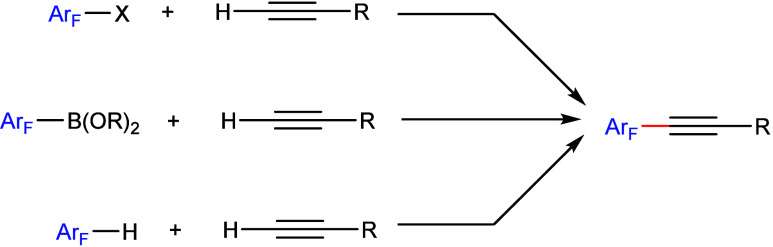
Three Different Routes to Synthesize Aryl Alkynes

The transition metal-catalyzed cross-coupling of electrophilic reagents such as aryl halides with terminal alkynes, namely the Sonogashira cross-coupling reaction, represents one of the most utilized methods to generate aryl alkynes.^163−166^ Alternatively, oxidative cross-coupling of a preactivated nucleophilic reagent, e.g., fluorinated aryl boronates with terminal alkynes, is also possible.^112^ However, direct alkynylation of arene C–H bonds to provide the same products would be desirable due to it being a straightforward route and a more efficient process (Scheme 36).

**Scheme 37:**
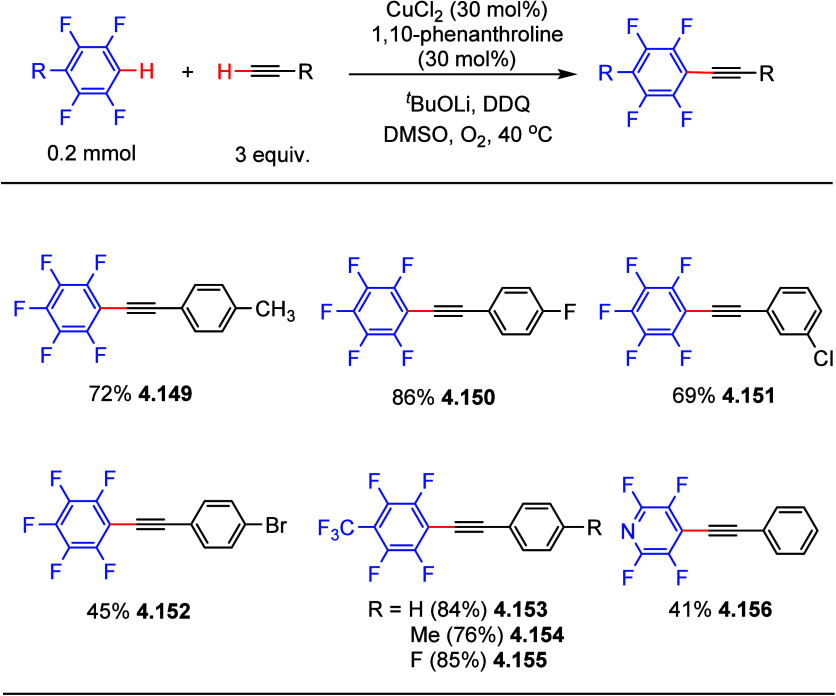
Copper-Catalyzed Direct Alkynylation of (Poly)fluoroarenes with Terminal Alkynes^167^

In 2010, Su et al. reported the copper-catalyzed direct alkynylation of electron-deficient (poly)fluoroarenes with terminal aryl and heteroarylalkynes using O_2_ as the oxidant to construct C_sp^2^_–C_sp_ bonds.^167^ A screening process led to optimized conditions to form the cross-coupling products selectively and minimize the generation of homocoupling products. Using these conditions, CuCl_2_ (30 mol%), phenanthroline (30 mol%), DDQ (15 mol%), and stoichiometric amounts of ^*t*^BuOLi (3 equiv) as the base in DMSO as the solvent, the cross-coupling products were generated in fair to good yields (Scheme 37). The conditions are viable for employment not only of arylalkynes bearing electron-donating substituents (e.g., methyl **4.149**, **4.154**), but also electron-withdrawing substituents (e.g., fluoride **4.150**, **4.155**, chloride **4.151**, and bromide **4.152**), providing a complementary platform for further functionalization. Interestingly, the heteroarene 2,3,5,6-tetrachloropyridine is also a viable substrate, giving the desired product in moderate yield (**4.156**).

**Scheme 38:**
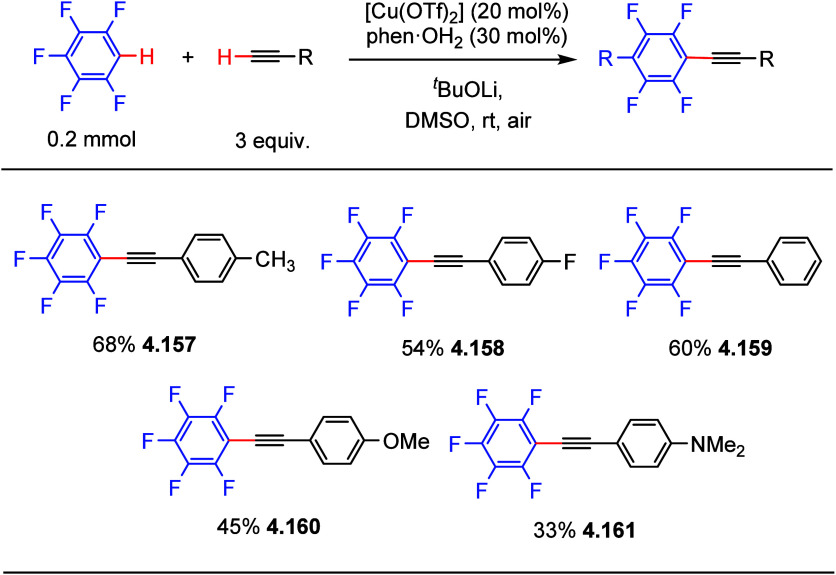
Direct C–H Alkynylation of (Poly)fluoroarenes with Terminal Alkynes Using a Copper Catalyst^168^

In the same year, similar conditions with a slightly different catalyst were also reported by Miura et al.,^168^ using a combination of [Cu(OTf)_2_] and phenanthroline monohydrate as the catalyst to generate C–H alkynylation products in moderate yields (Scheme 38). Both Su’s and Miura’s works have the same limitation that only (poly)fluoroarene substrates containing four or five fluorine substituents are viable substrates (Scheme 37 and Scheme 38).

### 4.6 Alkylation Reactions

#### 4.6.1 With Benzyl Chlorides

**Scheme 39:**
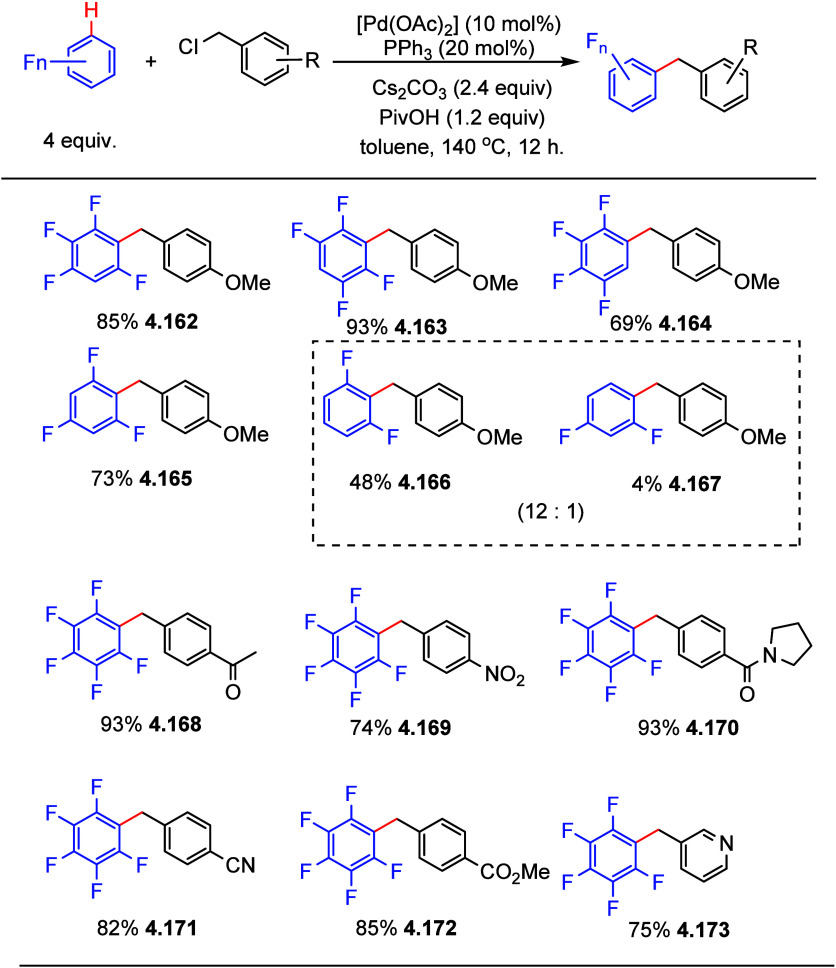
Palladium-Catalyzed Benzylation of (Poly)fluoroarenes^169^

After having developed a method for direct C–H olefination of (poly)fluoroarenes (see Section 4.7.1), Zhang reported the palladium-catalyzed benzylation of (poly)fluoroarenes with various benzyl chlorides with selected examples shown in Scheme 39.^169^ The substrate scope of (poly)fluoroarenes was extended to arenes containing 2–5 fluorine substituents (**4.162**–**4.173**), with a C–H bond flanked by two C–F bonds being the primary reaction site (**4.166**). The benzyl halide component can contain other versatile functional groups such as methyl ketone **4.168**, nitro **4.169**, amide **4.170**, nitrile **4.171**, esters **4.172** and azinyl nitrogen (pyridine) **4.173**, giving opportunities for further functionalization.

#### 4.6.2 With Vinyl Ketones

**Scheme 40:**
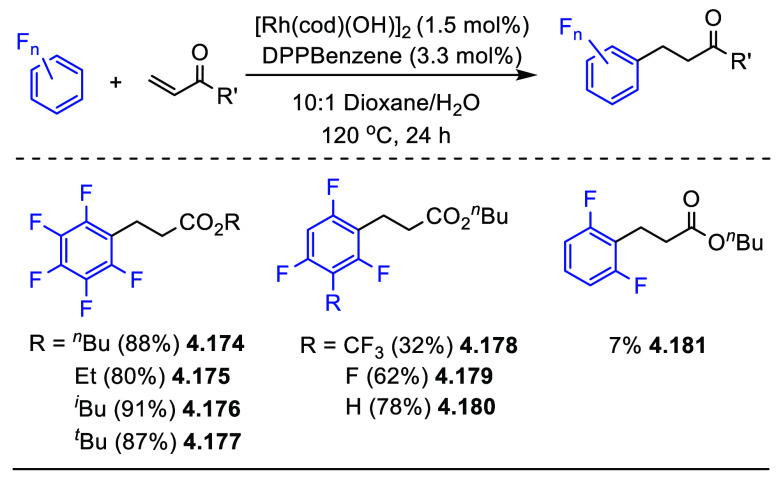
Rhodium-Catalyzed Alkylation of (Poly)fluoroarenes^171^

In 2009, Zhao reported decarboxylative conjugate addition of polyfluorinated benzoic acids in aqueous solvents using a rhodium catalyst.^170^ Inspired by earlier studies^80,155^ that reported direct C–H functionalization of (poly)fluoroarenes, Zhao et al.^171^ developed a method to directly alkylate (poly)fluoroarenes with α,β-unsaturated carbonyl derivatives using a combination of [Rh(cod)(OH)]_2_ as the precatalyst and 1,2-bis(diphenylphosphino)benzene (DPPBenzene) as the ligand in a mixed dioxane/H_2_O (10:1) solvent. Selected examples in Scheme 40 show that only arenes containing 3 to 5 fluorine substituents are viable substrates, generating alkylated arenes in fair to very good yields (**4.174**–**4.180**). It is important to note that removal of H_2_O slowed the reaction and changed the selectivity, favoring olefination which will be shown in Section 4.7.1.

#### 4.6.3 With Alkyl Aryl Carboxamides

**Scheme 41:**
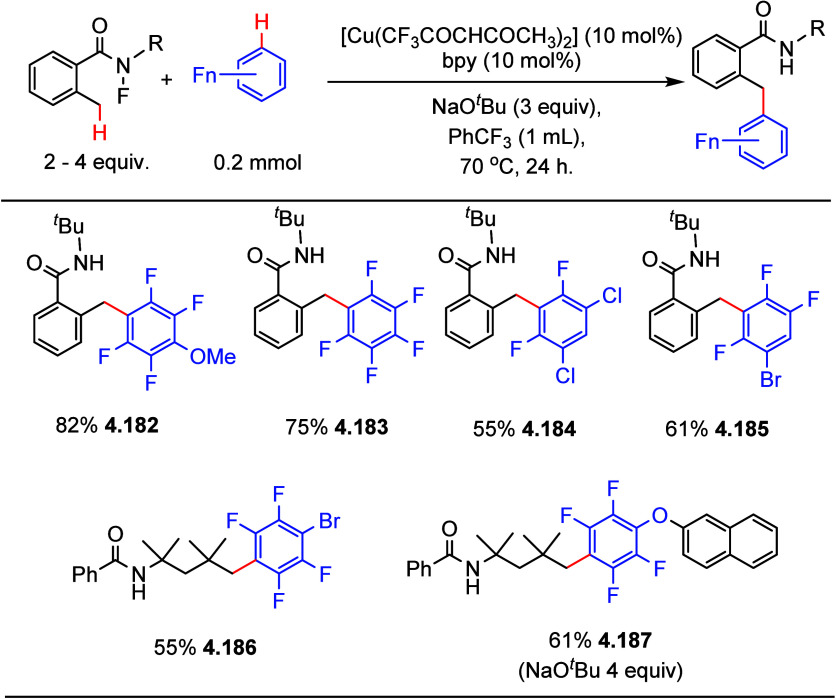
Copper-Catalyzed C–H Alkylation of Fluorinated Arenes via Remote C_sp^3^_–H Functionalization in Carboxamides^175^

The Hofmann–Löffler–Freytag (HLF) reaction, a 1,5-hydrogen atom transfer (1,5-HAT) to *N*-centered radicals, is a well-documented process.^172−174^ Liang et al.^175^ employed the C-centered radical generated from an HLF reaction to alkylate C–H (poly)fluoroarenes. Thus, in 2021, they reported the C–H alkylation of (poly)fluoroarenes via C_sp^3^_–H functionalization of aryls and amides. Using *N*-*tert*-butyl-*N*-fluoro-2-methylbenzamide and 2-(2,3,5,6-tetrafluorophenoxy)naphthalene as model substrates, [Cu(CF_3_COCHCOCH_3_)_2_] as the catalyst, NaO^*t*^Bu as the base, and 2,2′-bipyridine (bpy) as the ligand, and PhCF_3_ as a solvent at 70 °C, led, after 24 h, to the desired cross-coupling product in 60% yield. Selected examples in Scheme 41 show that the benzylic C–H bonds and aliphatic C–H bonds in *N*-fluorocarboxamides could be (poly)fluoroarylated smoothly with excellent regioselectivity (**4.182**–**4.187**). Notably, these conditions cannot be applied for fluorobenzene and 1,3-difluorobenzene substrates.

**Scheme 42:**
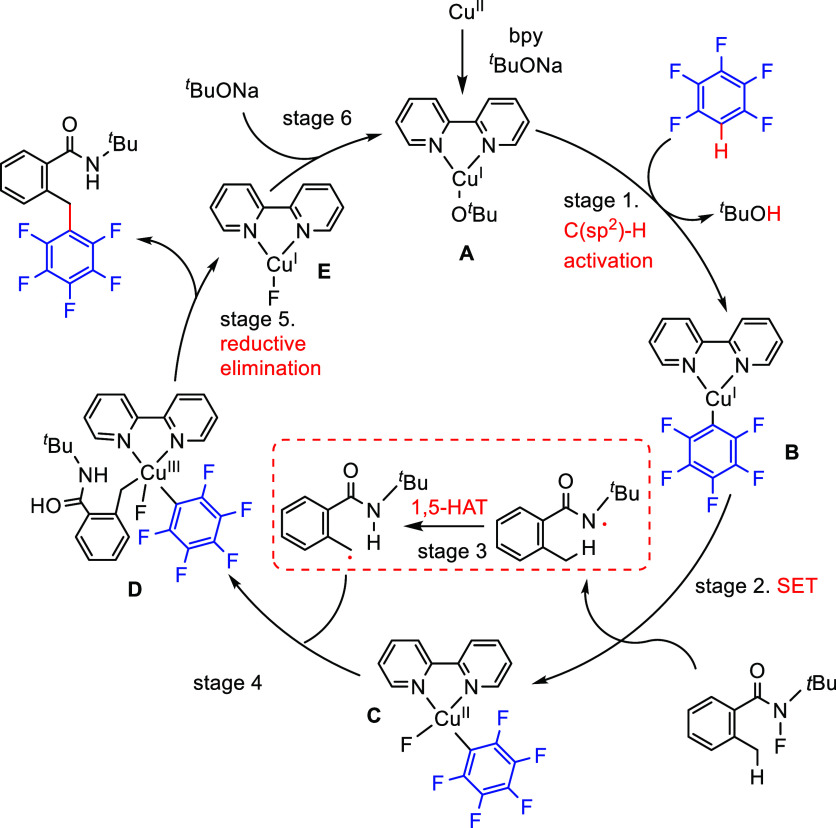
Proposed Reaction Pathway for Copper-Catalyzed C–H Alkylation^175^

Based on control experiments and DFT calculations, as well as on previous reports,^176,177^ Liang et al. proposed a mechanistic pathway for this copper-catalyzed C–H alkylation of (poly)fluoroarenes (Scheme 42). C_sp^2^_–H bond activation of pentafluorobenzene occurred with the help of an in situ-generated Cu(I) complex [Cu(bpy)(O^*t*^Bu)] **A** to yield the transmetalation product [Cu(bpy)(C_6_F_5_)] **B**, either by simple deprotonation or by direct hydrogen abstraction via a *tert*-butoxide free radical (Scheme 42, stage 1). Then, SET between carboxamides and complex **B** generated an amidyl radical and the Cu(II) species [Cu(bpy)(C_6_F_5_)(F)] **C** (stage 2). The amidyl radical led to 1,5-HAT to give carbon-centered radical (stage 3), which then combined with [Cu(bpy)(C_6_F_5_)(F)] **C** to generate a Cu(III) species **D** (stage 4). This is followed by reductive elimination to give the alkylated (poly)fluoroarene products and [Cu(bpy)(F)] **E** (stage 5). Subsequent ligand exchange between [Cu(bpy)(F)] **E** and ^*t*^BuONa closes the catalytic cycle (stage 6).

#### 4.6.4 With C_sp^3^_–H Bonds

**Scheme 43:**
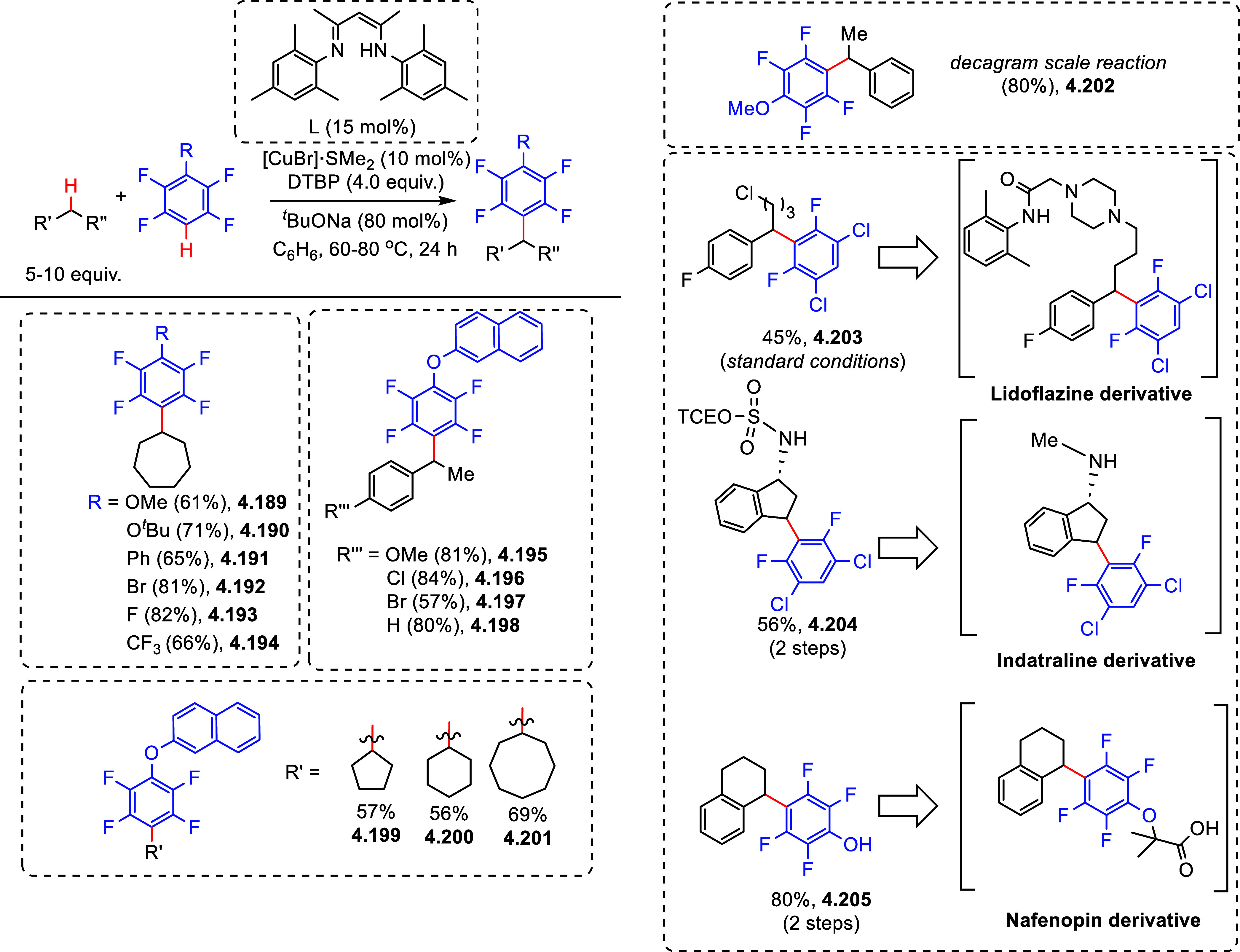
Copper-Catalyzed Direct C–H Alkylation of (Poly)fluoroarenes Using Hydrocarbons as Alkylating Agents^178^

Direct utilization of arenes and alkanes for chemoselective C_sp^2^_–C_sp^3^_ cross-couplings via direct dual C–H activation is highly demanding due to their low reactivity and the challenge of suppressing the formation of homocoupling side products. In 2020, Chang et al.^178^ reported the direct C–H alkylation of (poly)fluoroarenes with C_sp^3^_–H bonds of hydrocarbons using copper catalysis. During the optimization process, 28 ligands were screened in combination with catalytic amounts of copper salts, and the addition of oxidants and bases. The authors reported that the combination of [CuBr]·SMe_2_, and a ligand such as a β-diketiminate bearing *N*-trimethylphenyl (mesityl) substituents, in the presence of di-*tert*-butyl peroxide (DTBP) as the oxidant and ^*t*^BuONa as the base, gave the highest selectivity to the desired coupling products with respect to the homocoupling side products. Selected examples in Scheme 43 show that the C_sp^2^_–C_sp^3^_ cross-coupling products were obtained in fair to very good yields (**4.189**–**4.205**). Interestingly, halogenated substrates were employed without affecting the bromide and chloride substituents (**4.192**, **4.196**, **4.197**, **4.203**, **4.204**). This method was utilized on a decagram-scale to react 2,3,4,5-tetrafluoroanisole with ethylbenzene to generate the coupling product in 80% yield (**4.202**), and proved useful to produce precursors for fluorinated analogues of drugs such as indatraline, lidoflazine, and nafenopin in good to high yields (**4.203**–**4.205**). Notably, no alkylation products were observed when employing fluorobenzene, 1,2,3-trifluorobenzene, 1,2,4,5- tetrachlorobenzene, or 1,2,4,5-tetrabromobenzene.

**Scheme 44:**
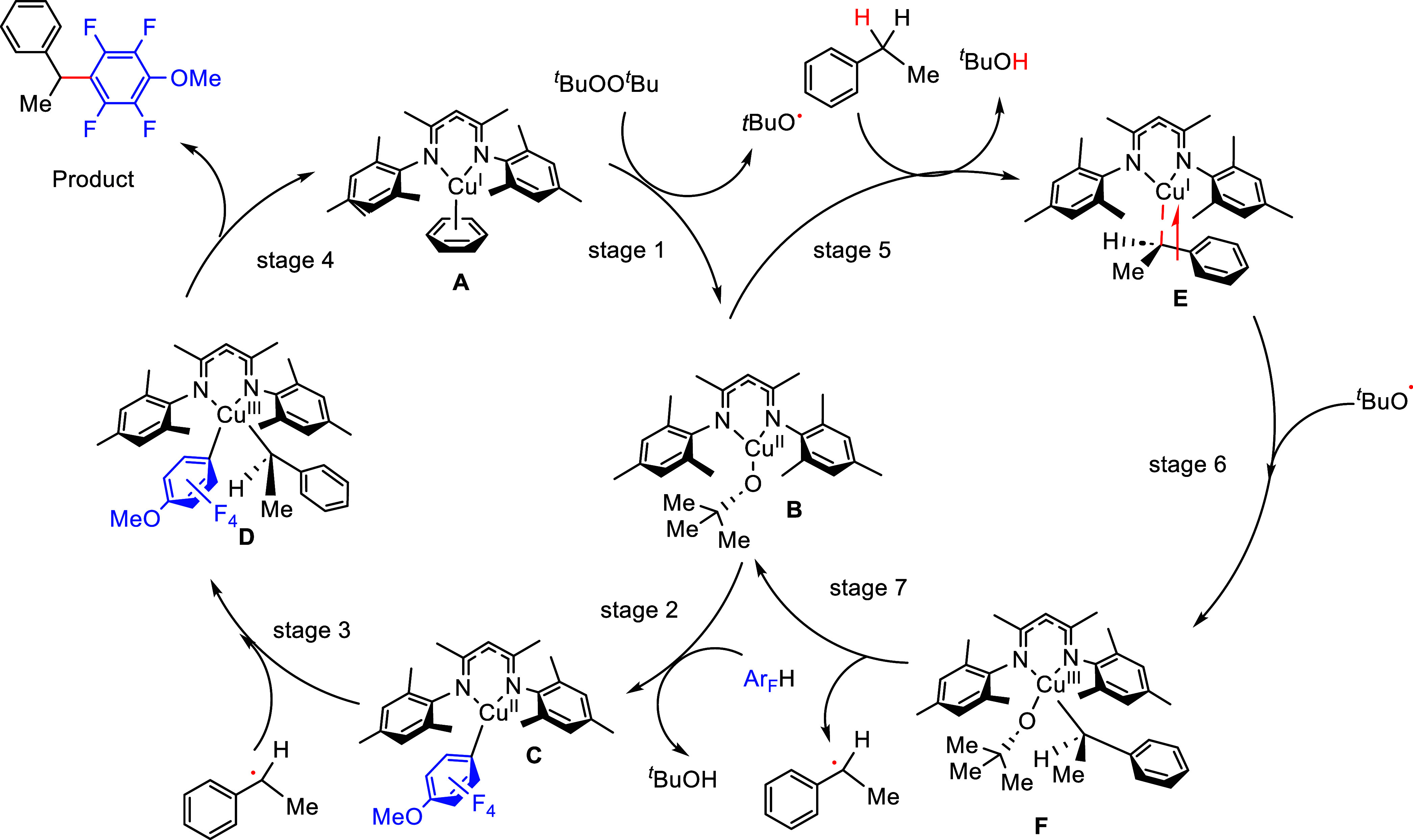
Proposed Mechanism for the Copper-Catalyzed Direct C–H Alkylation of (Poly)fluoroarenes with Hydrocarbons^178^

Based on radical trapping experiments, kinetic isotope effect studies, isolation of the active copper complex, and DFT calculations, the authors proposed a mechanism that involves ionic and radical pathways (Scheme 44). First, the reaction of the copper precursor with ligand and ^*t*^BuONa in benzene generates the active copper complex [LCu^I^(η^6^-benzene)] **A**, which then mediates the homolytic cleavage of DTBP to give intermediate [LCu^II^(O^*t*^Bu)] **B** and a ^*t*^BuO^•^ radical (stage 1).

Two pathways were then proposed for C–H bond activation. The first cycle involves C–H bond activation of the fluoroarene (Scheme 44 left side) and includes direct hydrogen atom abstraction from the (poly)fluoroarene by a ^*t*^BuO^•^ radical leading to [LCu^II^(Ar_F_)] **C** (stage 2) and the addition of the in situ-generated ethylbenzene radical to yield [LCu^III^(alkyl)(Ar_F_)] **D** (stage 3). Finally, reductive elimination from [LCu^III^(alkyl)(Ar_F_)] **D** occurs to release the allylated product and regenerate the active [LCu(η^6^-benzene)] complex **A** (stage 4). The second cycle (Scheme 44, right side) provides the alkyl radical via a copper-mediated activation process. For the alkoxy copper(II) species [LCu^II^(O^*t*^Bu)] **B**, radical character was found to be delocalized over the copper center and the oxygen atom, and a metal-centered process was proposed for C_sp^3^_–H bond activation leading to a Cu(I) alkyl radical intermediate **E** (stage 5), which then interacts with the ^*t*^BuO^•^ radical via an energetically high-lying copper(III) intermediate [LCu^III^(alkyl)(alkoxy)] **F** (stage 6), which releases the alkyl radical and reforms the copper alkoxide **B** (stage 7).

Interestingly, the analyses of the transition states (TS) for the copper-mediated activation of the C_sp^2^_–H bonds of the fluoroarene (TS between **B** and **C**, stage 2) and C_sp^3^_–H bond of ethylbenzene (TS between **B** and **E**, stage 5) revealed interesting differences in their spin density. For fluoroarene C–H activation, the spin density of the transition state was mainly localized at the copper center, leading to the proposal that C–H bond activation of (poly)fluoroarene takes place via an ionic reaction pathway. For ethylbenzene activation, significant radical character of the transition state was located on the *tert*-butoxy oxygen atom of the alkoxide ligand along with spin density on the benzylic carbon atom of the approaching ethylbenzene, implicating a radical pathway. This leads to the formation of a Cu(I) diradical species **E** (see Scheme 44), which then interacts with the ^*t*^BuO^•^ radical (via [LCu^III^(alkyl)(alkoxy)] **F**) to afford the ethylbenzene radical eventually. However, the postulated Cu(III) species **D** and **F** were not observed experimentally, and the reductive elimination of the proposed [LCu^III^(alkyl)(Ar_F_)] intermediate **D** was calculated to proceed almost barrierless (0.3 kcal/mol). Significantly, the experimental observation of the existence of a primary kinetic isotope effect of ethylbenzene demonstrated that C_sp^3^_–H bond cleavage is rate-limiting for this process, which is consistent with the computations reported.

**Scheme 45:**
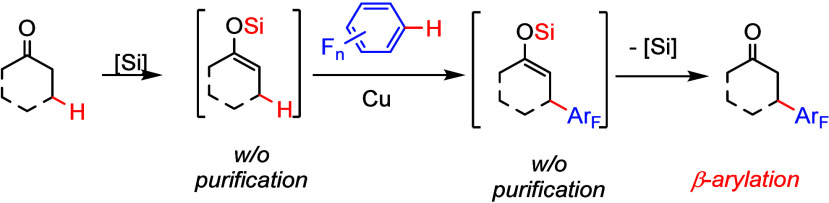
O-Silylation of Carbonyl Compounds Followed by Dehydrogenative Coupling with (Poly)fluoroarenes^176^

Molecules containing a carbonyl moiety are among the most widely used synthetic motifs.^179^ The α-arylation of carbonyls can be achieved by palladium catalysis^180−182^ or, alternatively, via silyl enol ethers.^183,184^ In contrast, only a few strategies were developed to functionalize the remote β-C–H bonds of carbonyl compounds, which is more challenging.^185−187^ In 2020, Chang et al. reported a new route to generate β-arylated carbonyls with (poly)fluoroarenes via a two-step process.^176^ As shown in Scheme 45, the strategy is initiated by the in situ O-silylation of the carbonyl group during the first step, followed by the copper-catalyzed reaction of the in situ-generated O-silyl enol ether at the allylic position, with the (poly)fluoroarenes leading to overall (poly)fluoroarylation at the β-position.

**Scheme 46:**
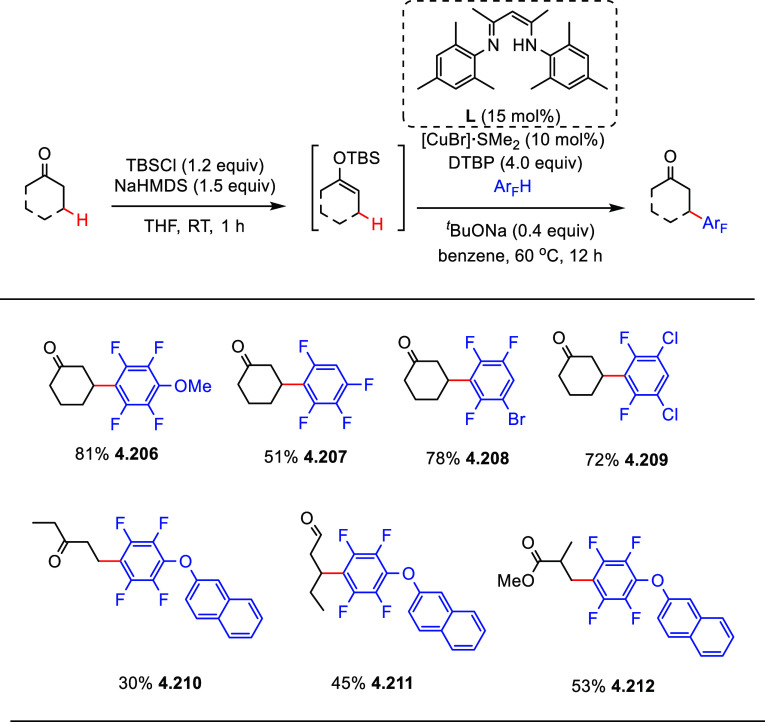
Scope of Copper-Catalyzed Formal Dehydrogenative Coupling of Carbonyl Compounds with (Poly)fluoroarenes to Give β-C–H Arylation^176^

After O-silylation at room temperature to generate O-silyl enol ethers (selected examples shown in Scheme 46), the allylic ether reacted under similar conditions to those used in the (poly)fluoroarylation reported in Scheme 43, i.e., via copper catalysis to give the desired products in fair to very good yields. This route is compatible with fluoroarenes containing bromo **4.208**, chloro **4.209**, and ether substituents **4.210**, and the carbonyl compounds used were cyclic and linear ketones **4.206**–**4.209**, aldehydes **4.211**, and esters **4.212**.

### 4.7 Olefination Reactions

#### 4.7.1 Olefination with Alkenes

**Scheme 47:**
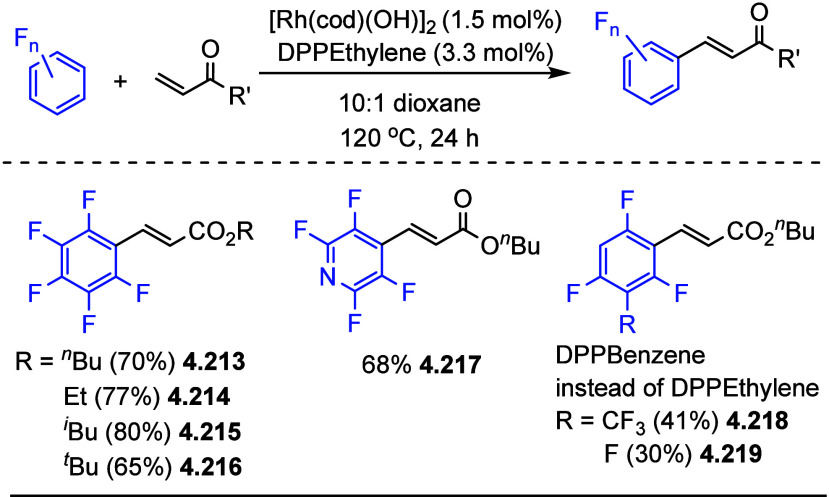
Rhodium-Catalyzed Olefination of (Poly)fluoroarenes with Vinyl Ketones Derivatives^171^

In 2001, Espinet and Milstein et al. reported the insertion of unsaturated molecules into a Pd–C_6_F_5_ bond, which plays a key role in Heck cross-coupling reactions.^188,189^ However, preactivated substrates, such as fluorinated aryl halides, were needed. In 2010, Zhao et al. reported olefination of electron-deficient (poly)fluoroarenes via rhodium catalysis.^171^ It is noted in Section 4.6.2, Scheme 40 that Zhao et al. reported that the combination of [Rh(cod)(OH)]_2_ and DPPBenzene catalyzes alkylation of fluoroarenes with vinyl ketone derivatives, in a mixed dioxane/H_2_O (10:1) solvent. Interestingly, removal of H_2_O in this process led to olefination. Changing the ligand to *cis*-1,2-bis(diphenylphosphino)ethylene (DPPEthylene) led to optimized conditions favoring olefination products in up to 80% yields (selected examples shown in Scheme 47). However, arenes containing less than 3 fluorine substituents were not investigated.

**Scheme 48:**
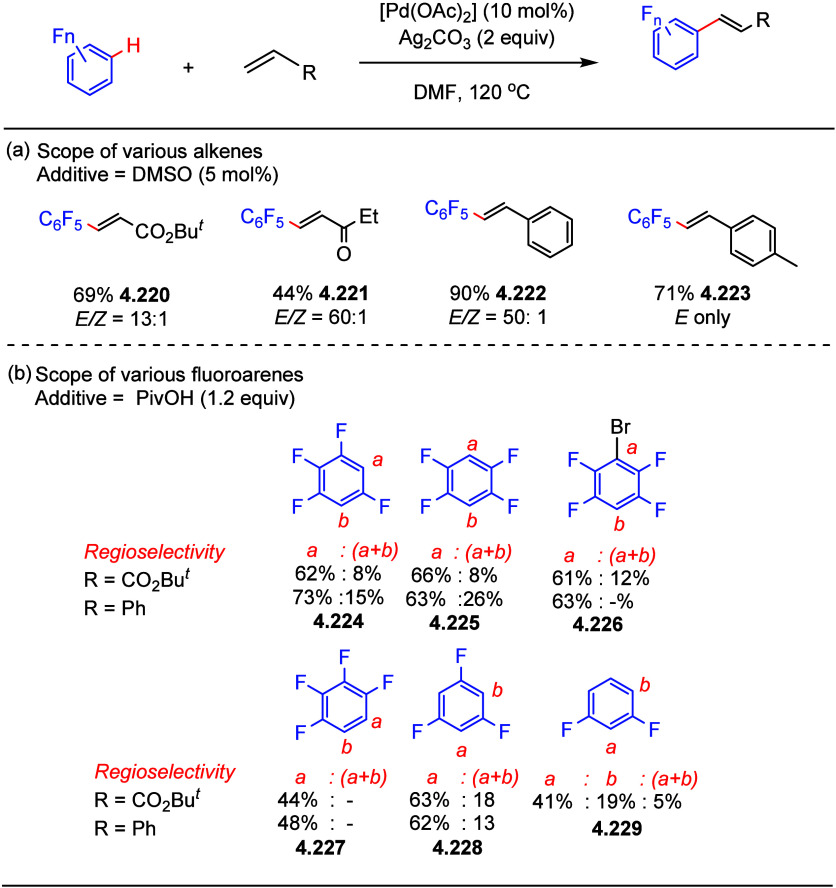
Oxidative Olefination of Fluoroarenes with Alkenes^190^

In the same year, Jiang et al. reported the direct C–H olefination of (poly)fluoroarenes, via an oxidative coupling process using [Pd(OAc)_2_] as the catalyst and Ag_2_CO_3_ as the base and oxidant.^190^ With addition of 5% of DMSO in DMF, this can generate pentafluorophenyl-substituted alkenes in up to 90% yield with *E*/*Z* stereoselectivities of up to 50:1. As shown in selected examples in Scheme 48a, in general, electron-rich alkenes gave higher yields than electron-deficient ones, giving alkenylation products in moderate to very good yields. Notably, other palladium catalysts such as PdCl_2_, [Pd_2_(dba)_3_], and [Pd(TFA)_2_], and other oxidants such as [Cu(OAc)_2_], oxone, 1,4-benzoquinone, PhI(OAc)_2_, and O_2_ showed lower or no activity.

Replacing the DMSO by PivOH (1.2 equiv) as the additive allowed other fluoroarenes to be used giving the alkenylation products in moderate yields with moderate to good regioselectivities (selected examples shown in Scheme 48b). It should be noted that C–H bonds flanked by two C–F bonds are more reactive than C–H bonds *ortho* to only one fluorine (**4.229**). Interestingly, fluoroarenes bearing a bromide substituent are viable (**4.226**), providing opportunities for further functionalization.

#### 4.7.2 Olefination with Allyl Esters and Ethers

**Scheme 49:**
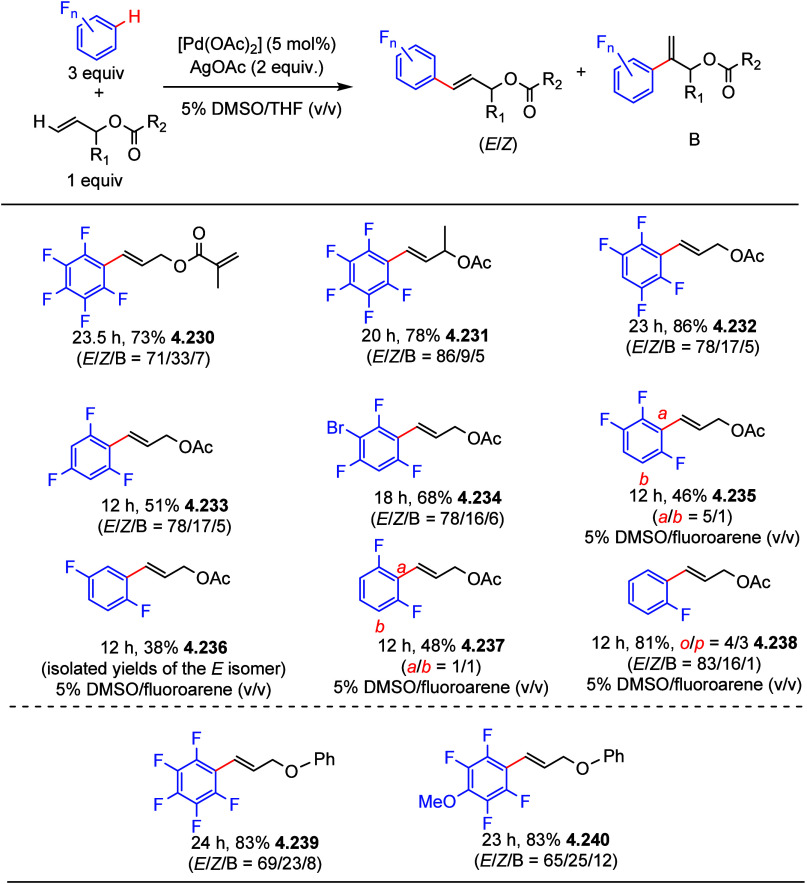
Palladium-Catalyzed Oxidative Heck Coupling of (Poly)fluoroarenes with Allyl Esters and Ethers^197^

**Scheme 50:**
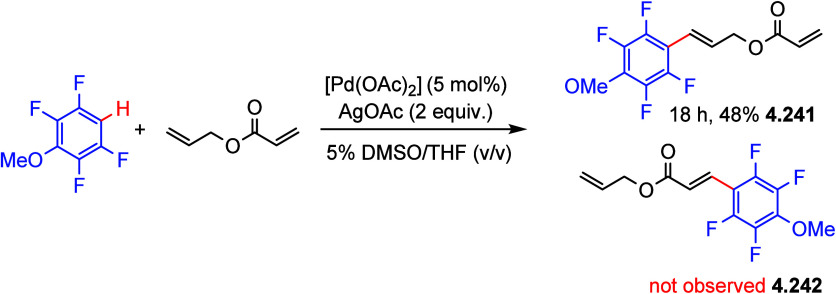
Comparison of the Reactivities of Allylic and Acrylic Double Bonds^197^

Allylic esters are commonly used as allylation reagents in organic synthesis.^191−194^ Jiao et al.,^194^ White et al.,^195^ and Xiao et al.^196^ reported oxidative Heck reactions of allylic esters with arylboronic acids, which occurred via β-hydride elimination rather than β-OAc elimination. In 2011, Liu et al.^197^ reported olefination of (poly)fluoroarenes with allyl esters via an oxidative coupling process using [Pd(OAc)_2_] as the catalyst and AgOAc as the base and the oxidant. With pentafluorobenzene and allyl acetate as the substrates, it was found that addition of DMSO in THF gave the desired product in 82% isolated yield. The scope of coupling reactions of various (poly)fluoroarenes with substituted allylic esters was examined (**4.230**–**4.240**). Selected examples in Scheme 49 show that olefination occurred at the C–H bond *ortho* to two fluorines (**4.235**). Furthermore, it was observed that the yields decreased with decreasing number of fluorine substituents on the arene rings. However, fluorobenzene can be allylated in 81% yield under nearly neat conditions (5% DMSO/fluorobenzene) (**4.238**). An allylic ether was employed successfully as the olefination agent under these conditions (**4.239**, **4.240**). The competition of reactivities between the allylic and acrylic double bonds was examined (Scheme 50), and it was found that the olefination occurred selectively at the terminal allylic C=C bond (**4.241**) rather than the acrylic double bond **4.242**.

#### 4.7.3 Olefination via Alkyne Insertion: Nickel-Catalyzed C-H Olefination of (Poly)fluoroarenes

**Scheme 51:**
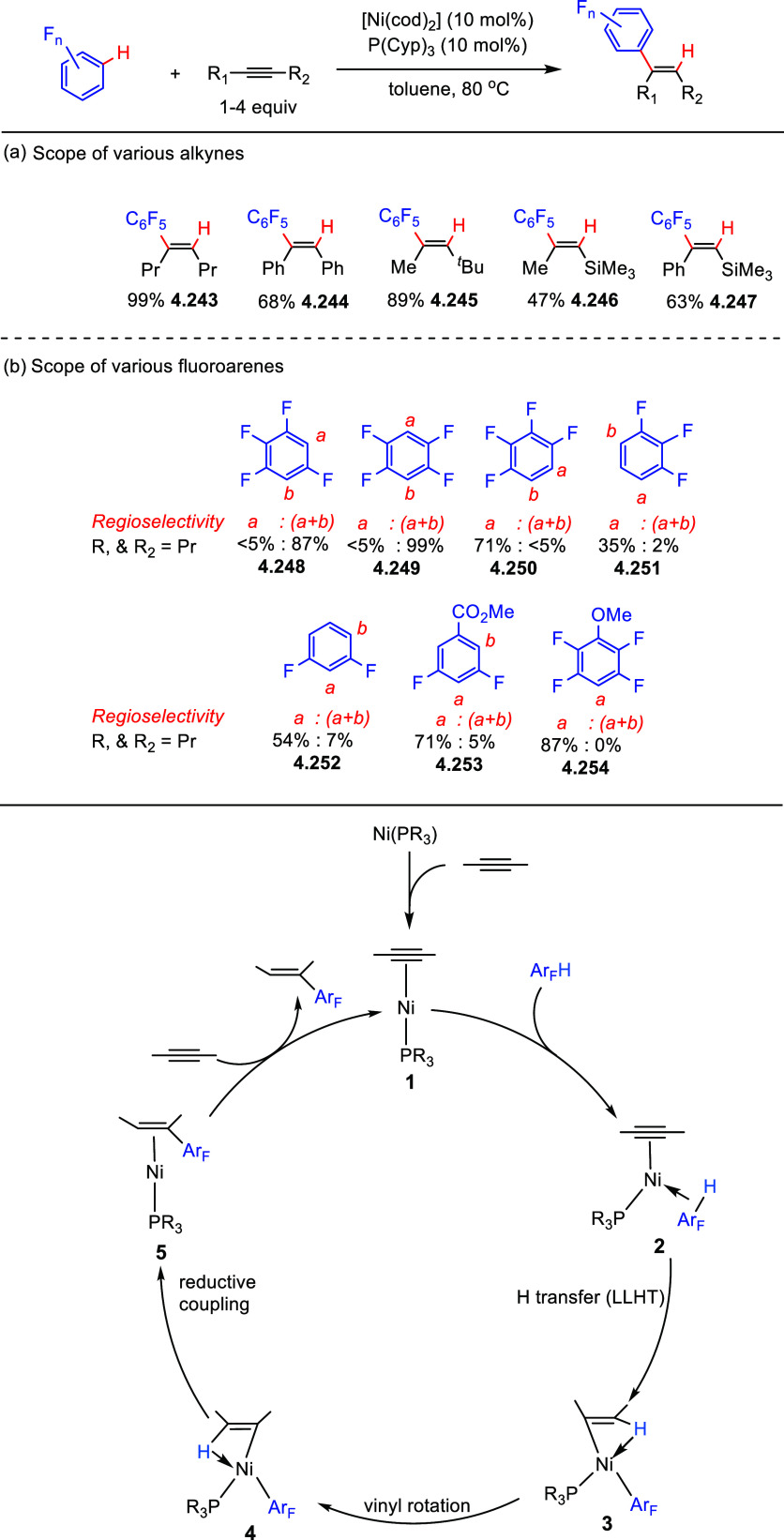
Alkenylation of (Poly)fluoroarenes Catalyzed by Nickel (Top) (205) and Postulated Catalytic Cycle Showing the LLHT Step (**2**–**3**) (Bottom)^206^

Several experimental^117,198−204^ and theoretical^78^ investigations have shown that Ni(0) complexes favor oxidative addition of C–F bonds of (poly)fluoroarenes rather than C–H bonds. In 2008, Radius et al. reported the catalytic activity of NHC-ligated Ni(0) complexes for C–F arylation of (poly)fluoroarenes with aryl halides.^200^ Also, in 2008, Nakao and Hiyama reported that reaction of pentafluorobenzene with 4-octyne led to alkenylation of (poly)fluoroarenes via activation of the C–H bond rather than the C–F bond, using a combination of precatalyst [Ni(cod)_2_] and ligand P(Cyp)_3_.^205^ Selected examples in Scheme 51 show that the reaction gave the corresponding *cis*-addition product in fair to excellent yields. The reactivity of other (poly)fluoroarenes (Scheme 51b) follows the now-familiar pattern; thus, C–H bonds flanked by two C–F bonds are more reactive than those *ortho* to one fluorine (**4.251**–**4.253**). Notably, excess alkyne led to dialkenylation rather than monoalkenylation. The presence of an electron-donating methoxy group or electron-withdrawing ester group did not affect the reaction (**4.253**–**4.354**). The mechanism of this reaction and the pattern of reactivity with different fluoroarenes has been examined by DFT methods by Eisenstein, Perutz, and colleagues.^206^ The reaction is proposed to proceed by a ligand-to-ligand hydrogen transfer mechanism (LLHT) in which a protonic hydrogen moves from σ-coordinated C–H bond of the arene to the coordinated alkyne (Scheme 51). The barriers to the various steps of reaction have been calculated for ten fluorinated benzenes and benzene itself. The energetic span^207^ of the catalytic reaction ranges from 102 to 125 kJ/mol, with the minimum values for arenes with two *ortho* fluorines. Moreover, the energy barrier for the C–H activation step correlates excellently with the Ni–C bond energy of the intermediate [Ni(PMe_3_)(Ar_F_)(MeCH=CHMe)] (**3** in Scheme 51, bottom) and far less well with the C–H bond energy. A correlation plot (compare Figure 2b) of the BDE of the Ni–C bond versus the BDE of the C–H bond yields a slope of 2.96, indicating the huge sensitivity of this BDE to the number of *ortho* fluorines. Thus, the Ni–C bond-forming step is controlled by the BDE of this bond and hence by the number of *ortho* fluorines.

**Scheme 52:**
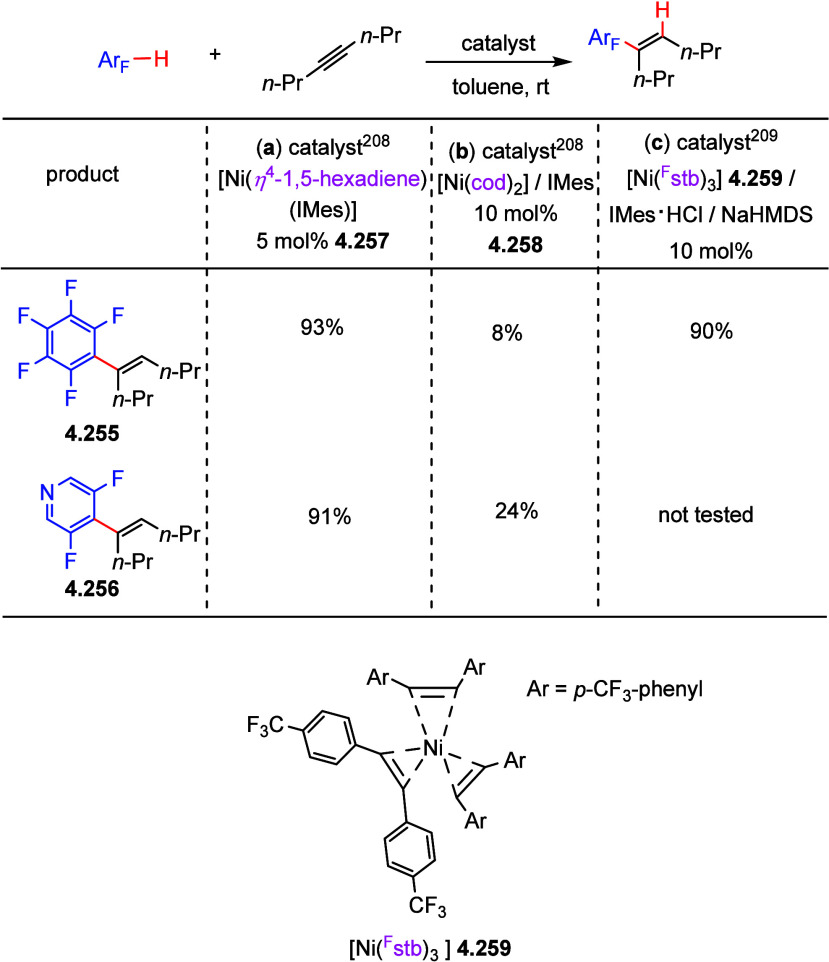
Nickel Precatalyst Comparison with and without cod as an Ancillary Ligand at Room Temperature^208,209^

**Scheme 53:**
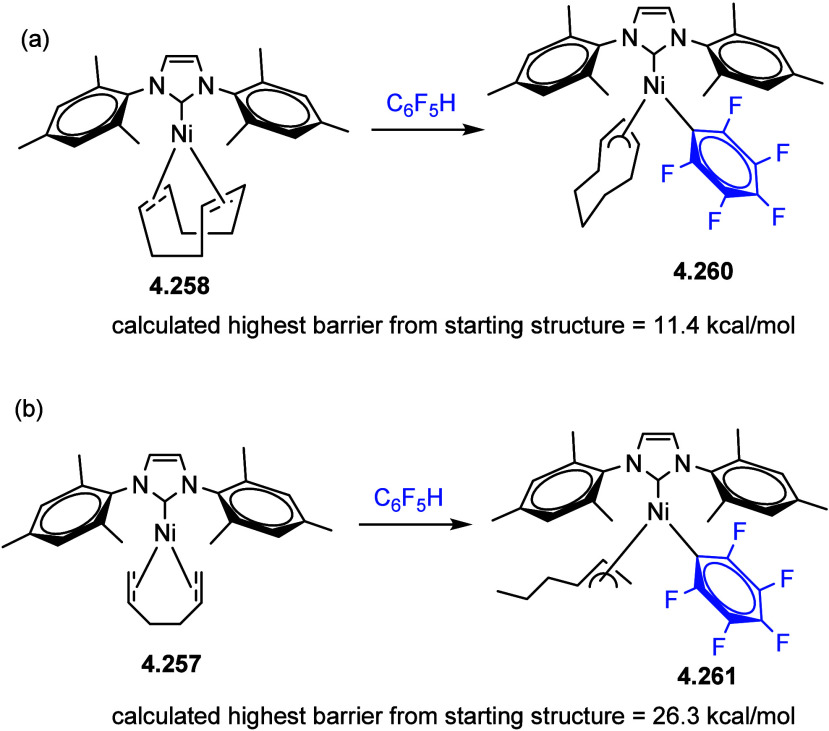
Formation of π-Allyl Nickel Complexes As Off-Cycle Intermediates via Hydride Migration from Fluoroarenes^208^

In 2015, Zimmerman and Montgomery et al. reported C–H alkenylation of (poly)fluoroarenes at room temperature, using the more active [Ni(η^4^-1,5-hexadiene)(IMes)] (IMes = 1,3-dimesitylimidazol-2-ylidene) (**4.257**) as the catalyst precursor (Scheme 52a).^208^ Conversely, utilizing [Ni(cod)_2_]/IMes (**4.258**) instead of [Ni(η^4^-1,5-hexadiene)(IMes)] (**4.257**) gave poor yields (Scheme 52b). Experimental and computational studies demonstrated that cod can inhibit the C–H functionalization processes via migration of the activated H (from fluoroarenes) to cod to generate off-cycle π-allyl complexes (**4.260**) (Scheme 53). Computational studies indicated that the highest barrier for this process is only 11.4 kcal/mol with [Ni(cod)(IMes)] (**4.258**), and experimental studies revealed that this process is achievable at room temperature (Scheme 53a). Using 1,5-hexadiene as the ancillary ligand instead of cod avoids the formation of similar off-cycle intermediates (**4.261**), as the highest barrier for this process is 26.3 kcal/mol with [Ni(η^4^-1,5-hexadiene)(IMes)] (**4.257**), and experimental studies have shown that this process does not occur at room temperature (Scheme 53b). Notably, the authors also postulate that the C–H activation step proceeds via an LLHT mechanism.

Previously, Ni(0)-olefin precatalysts (including the ones in Scheme 53) were known for their instability and rapid decomposition in air. In 2020, Cornella et al.^209^ reported an air-stable binary nickel complex, [Ni(^F^stb)_3_] **4.259**, which contains *p*-CF_3_-stilbene derivatives as ligands, making the nickel center very stable under aerobic conditions. In contrast to all reported 16- and 18-electron Ni(0)-olefin precatalysts, [Ni(^F^stb)_3_] **4.259** was reported to be stable in air for months without apparent decomposition. The use of catalytic amounts of [Ni(^F^stb)_3_] **4.259**, with the air-stable carbene salt IMes·HCl as a ligand and NaHMDS as a base was also tested for alkenylation of pentafluorobenzene, and the desired product **4.255** was produced in very good yield (Scheme 52c).

## 5 C–N Bond Forming Reactions

### 5.1 Amination Reactions with Amines

**Scheme 54:**
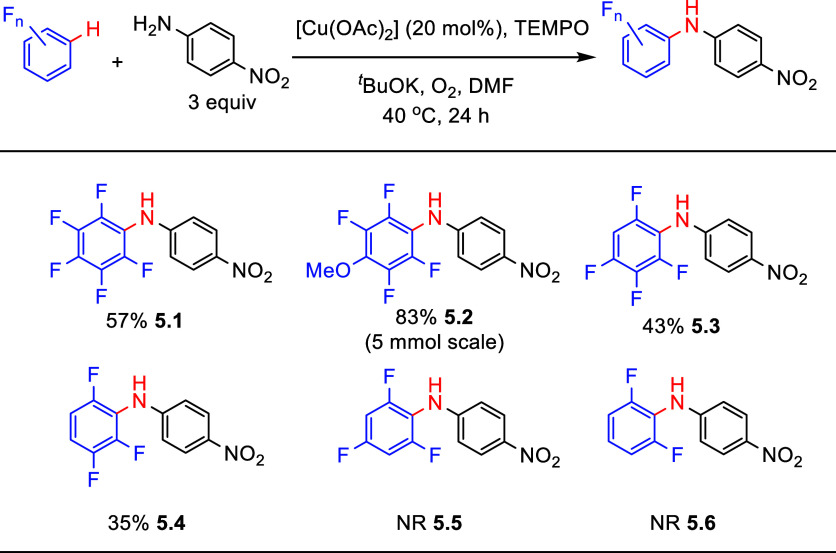
Copper-Catalyzed C–H Amination of (Poly)fluoroarenes with Amines^214^

The abundance of aromatic amines in agrochemicals^210^ and pharmaceuticals^211^ encourages researchers to develop new methods for their construction. A traditional route to form C–N bonds is via C–X amination; however, this requires preactivated aryl halides and high-temperature reactions for completion.^212,213^ The report by Daugulis in 2007 on direct C–H arylation of acidic C–H bonds of fluoroarenes with a copper catalysis system^144^ inspired Su et al., in 2010,^214^ to study the possibility of C–H amination of (poly)fluoroarenes with a copper catalyst under milder conditions. As the coupling partners are both nucleophiles, the reaction was carried out under oxidative conditions. Optimization (selected examples shown in Scheme 54) showed that TEMPO was the best oxidant in combination with an O_2_ atmosphere for the copper-catalyzed direct C–H amination reaction of fluoroarenes with 4-nitroaniline. Using TEMPO as the only oxidant, i.e., without O_2_, led to very poor yields. The substrate scope was explored, and all (poly)fluoroarenes with 4 or 5 fluorine substituents were aminated with acceptable yields (**5.1–5.3**). While 1,2,4-trifluorobenzene can be aminated (**5.4**), 1,3,5-trifluorobenzene and 1,3-difluorobenzene gave no product (**5.5–5.6**). This selectivity was attributed to the greater acidity of the C–H bond in 1,2,4-trifluorobenzene compared to those in 1,3,5-trifluorobenzene and 1,3-difluorobenzene, but there has been no analysis of the determining factors comparable to that for hydrofluoroarylation described in Section 4.7.3.

### 5.2 Amidation Reactions with *N*-Chlorocarbamates

**Scheme 55:**
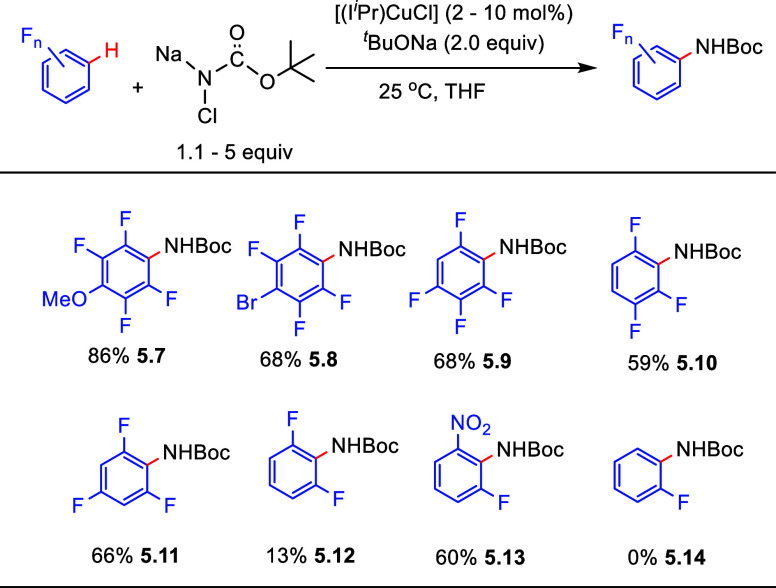
Copper-Catalyzed C–H Amidation of Fluoroarenes^215^

In Section 5.1, it was noted that Su et al. reported the amination of C–H fluoroarenes using a copper catalyst.^214^ In 2016, Chang et al. developed the C–H amidation of fluoroarenes with deprotectable *N*-chlorocarbamates, with the aid of an (NHC)Cu catalyst.^215^ The reaction proceeds under mild conditions (at 25 °C), in the presence of an alkoxide base such as ^*t*^BuONa. Under the optimized conditions described in Scheme 55, a range of (poly)fluoroarenes were examined. Selected examples show that arenes containing four and three fluorine substituents were viable (**5.7–5.11**), and the amidation took place preferably at the C–H bond flanked by two C–F bonds. Interestingly, while 1,3-difluorobenzene was not an effective substrate (**5.12**), the C–H bond *ortho* to both a fluorine and a nitro group can be amidated in fair yield (**5.13**). Benzenes containing only one electron-withdrawing group, such as fluorobenzene, failed in this process (**5.14**).

**Scheme 56:**
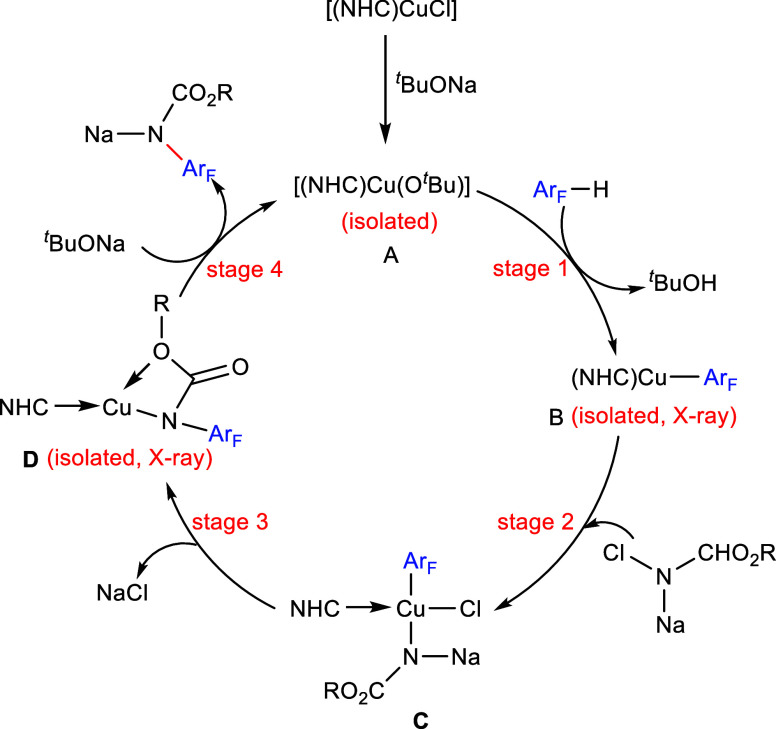
Proposed Mechanism for the Copper-Catalyzed Amidation of Arenes^215^

A mechanistic study was conducted exploring intermolecular kinetic isotope effects, H/D exchange, electron transfer inhibitors with TEMPO, and X-ray crystallography. First, exchange occurred between the chloro ligand of [(NHC)CuCl]^216,217^ and the alkoxide base to yield [(NHC)Cu(O^*t*^Bu)] **A**, which subsequently reacts with arene substrates generating [(NHC)Cu(Ar)] **B** (Scheme 56, stage 1). Then, oxidative addition of the *N*-chlorocarbamates at the Cu(I) center formed [(NHC)Cu(Ar)(Cl)(NNaCO_2_R)] **C** (stage 2). This Cu(III) species **C** transfers an aryl group to the carbamate moiety, affording a Cu(I) carbamato intermediate **D** in which the desired C–N bond is formed (stage 3). Finally, ligand exchange with ^*t*^BuONa releases the amidation product and closes the catalytic cycle (stage 4).

## 6 Hydroxylation Reactions with Oxygen

**Scheme 57:**
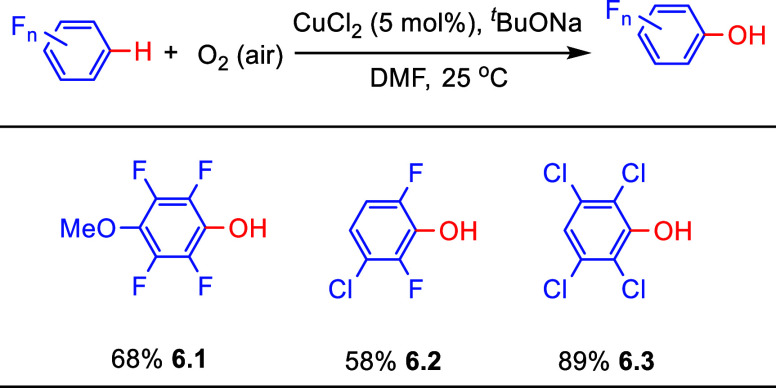
Copper-Catalyzed Oxidation of Electron-Deficient Arenes^222^

Phenols are important motifs as building blocks in organic synthesis, applied widely in pharmaceutical and polymer chemistry.^218^ Oxidation of arenes with O_2_ to form phenols is regarded as one of the main challenges in catalysis. Oxidation of benzene to phenol using a palladium catalyst with molecular oxygen requires high reaction temperatures and a high catalyst loading.^219−221^ In 2012, Lei et al. reported the copper-catalyzed oxidation of electron-deficient arenes and heteroarenes at room temperature in air.^222^ Under the reaction conditions described in Scheme 57, selected examples show that C–H bonds in the most acidic arenes containing three and four electron-withdrawing substituents such as fluorines and chlorines were oxidized to OH in moderate to good yields (**6.1-6.3**).

**Scheme 58:**
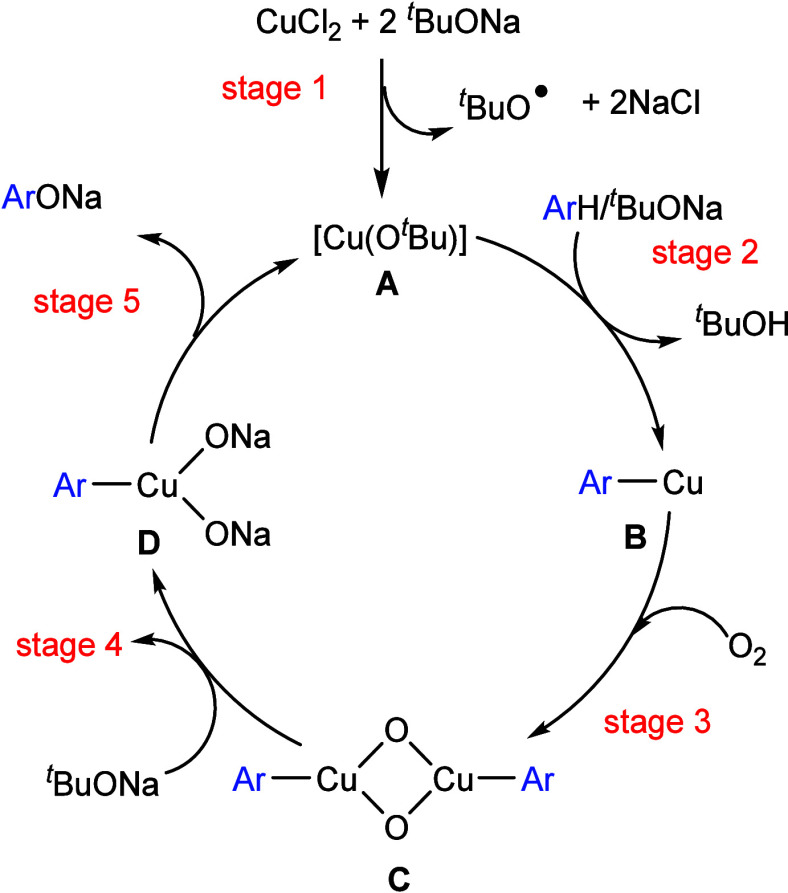
Proposed Mechanism of the Copper-Catalyzed Oxidation of Electron-Deficient Arenes^222^

Based on radical trapping, deuteration experiments, and kinetic profiles, the proposed reaction mechanism is depicted in Scheme 58. First, reduction of CuCl_2_ by NaO^*t*^Bu forms CuCl via an SET process, and this Cu(I) species reacts with NaO^*t*^Bu to form [Cu(O^*t*^Bu)] **A** (Scheme 58, stage 1). Next, transmetalation between [Cu(O^*t*^Bu)] with ArNa gives [ArCu^I^] **B** and regenerates NaO^*t*^Bu (stage 2). Then, the [ArCu^I^] **B** species is oxidized by O_2_ to form bridging complex [(Ar)_2_Cu_2_ (μ-O)_2_]^2+^**C** (stage 3). This is followed by nucleophilic attack with NaO^*t*^Bu to break the [Cu_2_(μ-O)_2_]^2+^ dimer to give intermediate complex [ArCu(ONa)_2_] **D** (stage 4), which undergoes reductive elimination producing ArONa (stage 5). This, on subsequent aqueous workup, led to the formation of ArOH.

## 7 Conclusions and Perspectives

Building on the pioneering methods reported by Fagnou et al.^80^ in 2006 and Daugulis et al.^155^ in 2007, a significant number of reports concerning direct C–H arylation of (poly)fluoroarenes have been reported, providing shorter protocols compared to preactivated routes using (poly)fluoroaryl boronates;^99^ zinc,^100,101^ magnesium,^102^ or lithium reagents;^103,104^ silanes;^105^ etc. These methods enable the C–H bond of (poly)fluoroarenes to be converted to aryl, heteroaryl, allyl, carbonyl, alkynyl, alkyl, olefin, amine, and phenol. As such, this provides an alternative process to conventional coupling methods. Even though the methodology for direct conversion of C–H bonds of fluoroarenes is not as broad as that via preactivated organometallic reagents, further developments are to be expected. Thus, the next challenges will be:

1) direct conversion of the C–H bond of fluorinated arenes to ethers, carboxylic acids, esters, nitro, or cyano groups for which no procedure currently exists;
2) reports on transformations of the C–H *ortho* to fluorine toward carbonylation, alkynylation, amination, amidation, and hydroxylation need further development as, currently, these transformations are limited to one or two methods;
3) in contrast to C–H borylation, allylation, and olefination, some of the existing methods for direct C–H functionalization of (poly)fluoroarenes are limited to arenes containing a large number of fluorine atoms (for example, the direct C–H carbonylation, alkynylation, alkylation, amidation, amination, and hydroxylation gave no reaction or low yields if the arene substrate has three or fewer fluorine atoms. Similarly, with the exception of the report by Hartwig (Section 4.1.1.1), most direct C–H arylations reported in the past decade, (see Section 4.1) did not employ fluorinated benzene substrates containing three or fewer fluorine substituents);
4) many of the transformations still require the use of a metal, and in a number of cases, a precious metal catalyst, and future work will likely focus on using greener, more sustainable methodologies to generate this remarkably useful family of compounds; and
5) more implementation using these direct transformations to synthesize more valuable compounds is desirable, for example, Chang et al. (see Section 4.6.4) gave examples of direct C–H alkylation of (poly)fluoroarenes to create fluorinated building units for the syntheses of lidoflazine, indatraline, and nafenopin derivatives.

In conclusion, this review demonstrates the widespread occurrence of *ortho* to C–F selectivity in cases where there is a choice, such as 1,3-difluorobenzene or 1,2,4-trifluorobenzene. However, there is still a lack of appreciation of the role of M–C bond dissociation energies in determining this selectivity and an overemphasis on C–H acidity (Section 2). Metal–carbon bonds greatly amplify the difference in bond energies relative to H–C bonds for arenes with two *ortho* fluorines over one or zero *ortho* fluorines. The discovery of the role of Ag(I) in C–H activation has prompted a rethink of reaction mechanisms with Ag(I) additives, but there remains a lack of curiosity about silver chemistry among those proposing reaction mechanisms. These discoveries bring Ag(I) into line with the established ability of its congeners Cu(I) and Au(I) summarized in this review.
